# Predicting synonymous codon usage and optimizing the heterologous gene for expression in *E. coli*

**DOI:** 10.1038/s41598-017-10546-0

**Published:** 2017-08-30

**Authors:** Jian Tian, Yaru Yan, Qingxia Yue, Xiaoqing Liu, Xiaoyu Chu, Ningfeng Wu, Yunliu Fan

**Affiliations:** 1 Biotechnology Research Institute, Chinese Academy of Agricultural Sciences, Beijing, 100081 China; 2 College of Food Science and Technology, Agricultural University of Hebei, Baoding, HeBei Provice 071001 China; 3 Institute of Microbial Biotechnology, Jinan University, Guangzhou, Guangdong Province 510632 China

## Abstract

Of the 20 common amino acids, 18 are encoded by multiple synonymous codons. These synonymous codons are not redundant; in fact, all of codons contribute substantially to protein expression, structure and function. In this study, the codon usage pattern of genes in the *E. coli* was learned from the sequenced genomes of *E. coli*. A machine learning based method, Presyncodon was proposed to predict synonymous codon selection in *E. coli* based on the learned codon usage patterns of the residue in the context of the specific fragment. The predicting results indicate that Presycoden could be used to predict synonymous codon selection of the gene in the *E. coli* with the high accuracy. Two reporter genes (*egfp* and *mApple*) were designed with a combination of low- and high-frequency-usage codons by the method. The fluorescence intensity of eGFP and mApple expressed by the (*egfp* and *mApple*) designed by this method was about 2.3- or 1.7- folds greater than that from the genes with only high-frequency-usage codons in *E. coli*. Therefore, both low- and high-frequency-usage codons make positive contributions to the functional expression of the heterologous proteins. This method could be used to design synthetic genes for heterologous gene expression in biotechnology.

## Introduction

In naturally occurring genes, 61 codons code for the 20 common amino acids. The role of synonymous codons is unclear, as they do not alter the encoded amino acid sequence^1^. Therefore, it was initially thought that they would not affect cellular function, organismal fitness or evolution^2, 3^. However, several studies have found that synonymous codon selection in a gene could affect the expression^4–6^, structure and function of the encoded protein^7–9^. Therefore, it is useful to know the rules governing synonymous codon selection of the target gene, as such knowledge could enable us to design the heterogenous gene with the most efficient expression in the expression host.

The synonymous sequences contain varying ratios of low-frequency-usage (i.e., more slowly translated) to high-frequency-usage codons, which could control the translation speed of a protein^10, 11^. If a structural element within a protein is not translated with the appropriate speed, it can affect the folding the synthesized protein fragment and the assembly the structural elements of the protein^7^. Several studies have examined the overall translational rate of various protein structural elements^12–15^. For example, high-frequency-usage codons are mainly associated with α-helices. However, lower-frequency-usage codons are more likely to be associated with β-strands, random coils, structural domain boundaries and trans-membrane helices^7, 12, 14, 16^. The translation speed decreases on transition from coil to helix or strand^13^. As a result, the synonymous codons could affect the kinetics of translation and regulate the timing of protein synthesis at the local or global scales^10, 17^. In addition to the synonymous codon usage, many studies found that the codon pair usage, also known as codon context could affect the protein expression in the host^18–20^. Therefore, to express a target gene efficiently in a heterologous expression system, the gene should be designed based on the codon usage pattern of the host strain of the expression system and the codon selection constraints of the target gene^21–24^.

Many methods, including COOL^25^, Gene Designer^26^, Gene composer^27^, JCat^28^, COStar^29^ and OPTIMIZER^30^, have been proposed to design heterologous genes which are expected to be efficiently expressed in the host organism. Based on our knowledge, these methods are prone to select the high-frequency-usage codons of the host for the heterologous gene and neglect the contribution of the low-frequency-usage codons (rare codons) to the expression of the target gene. These approaches have been successfully used for the heterologous production of some proteins, especially the proteins also encoded by the “preferred” codons in the native host^31, 32^. However, in some cases, the high levels of protein expressed with high speed translation from the N- to C-terminal have led to the formation of insoluble products or degradation by protease enzymes, due to the incorrect folding^33, 34^.

The low expression or formation of insoluble aggregates may be attributable to differences in synonymous codon bias between the expression and natural hosts^35^. Another recent approach to encode a target gene sequence in the heterologous host is to “match” the codon usage bias inherent in the native host and is referred to as “codon harmonization”^14, 35, 36^. This codon harmonization approach was successfully applied to express several proteins in *E. coli* ^35–37^. However, if we did not know the codon usage bias of the native host, such as the gene cloned from the metagenome, this method could not be used. In addition, it is difficult to perfectly “match” the codon usage bias of the native host to the expression host. Therefore, a method should be developed to design the heterogenous gene with the appropriate synonymous codons in the expression host.

In this study, data from bacterial genomes in GenBank were used to analyze the rules of the synonymous codon selection in *Escherichia coli*. The codon usage pattern of a residue within a specific fragment was learned from all *E. coli* genomes in the GenBank, and this information was stored in index files. Based on those index files, a machine-learning method named Presyncodon was developed to predict synonymous codon (low- or high-frequency-usage codon) selection in a gene. The two reporter genes, encoding enhanced green fluorescent protein (*egfp*) and red fluorescent protein (*mApple*), were designed with the method. Expression of the designed genes yielded a higher fluorescence intensity compared with the genes in which low-frequency-usage codons had been replaced by high-frequency-usage codons. This result revealed that both low- and high-frequency-usage codons make positive contributions to the solubility of expressed recombinant proteins. In addition, this study will help us to understand codon selection rules and design genes that are more amenable to heterologous expression.

## Results

### Codon Usage Patterns of Different Bacterial Species

The sequences of 346 genomes of bacterial subspecies were collected from the NCBI database (Table S1), including five subspecies from 69 bacterial species and one subspecies from one species (*Bacteroides fragilis* NCTC 9343) selected as the out-group^38^. This dataset allowed us to easily evaluate the clustering results, as the five subspecies should be clustered into one group. The selected bacteria represented 38 families, 47 genera and 70 species. An evolutionary tree based on the 16 S rDNA of these subspecies was constructed. The 16 S rDNA tree in the Newick format is shown in Fig. S1. As shown in Figs S2A and S3, the five subspecies from each of the species were clustered into one group except for those from two closely related genera, *Bacillus* (*B. anthracis*, *B. cereus* and *B. thuringiensis*) and *Mycobacterium* (*M. canettii*, *M. bovis* and *M. tuberculosis*), which cross-clustered into one large group. In addition, species within the same genus and family clustered together.

**Figure 1:**
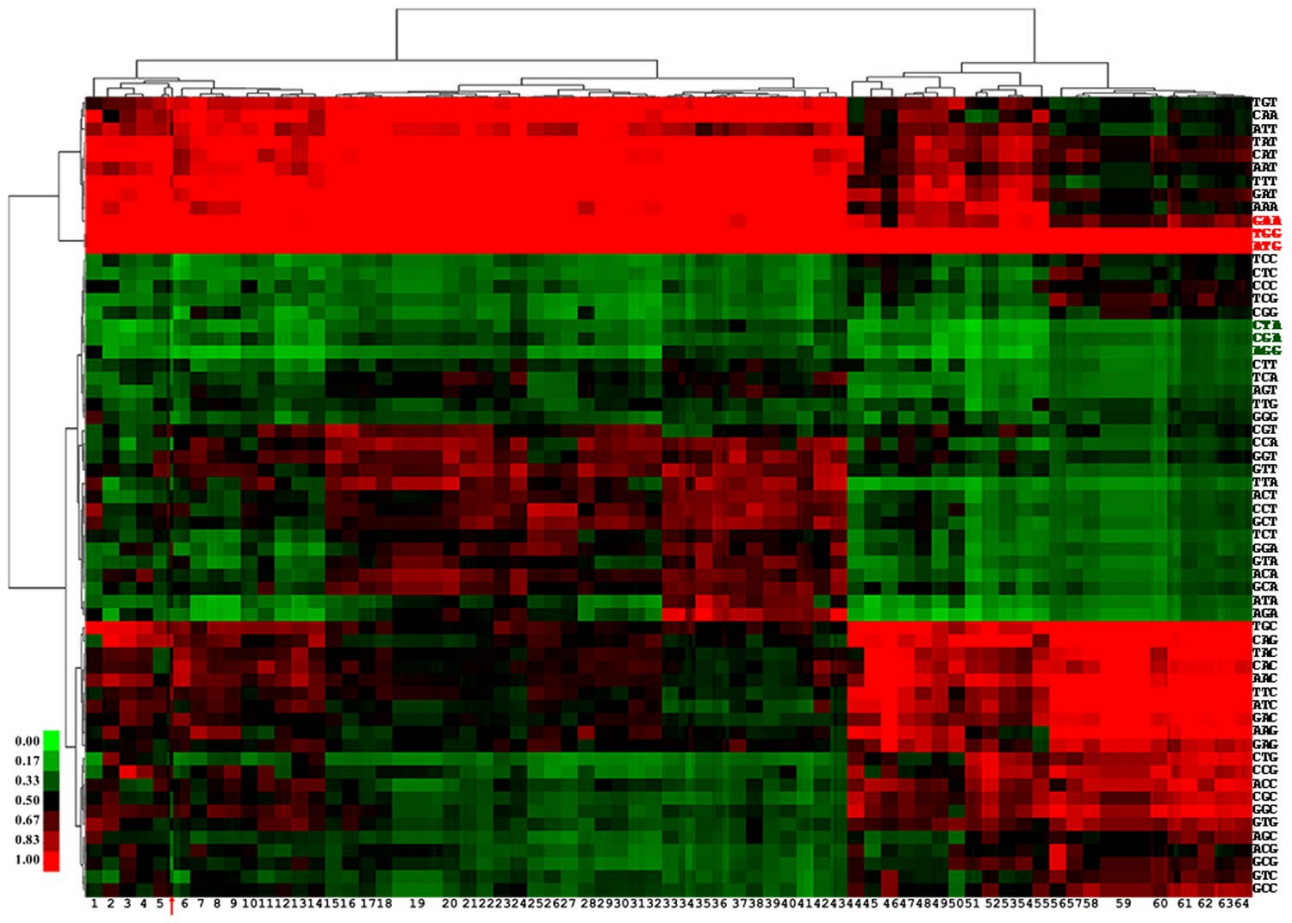
Clustering results of the codon usage pattern of different species. The row and column represent the codon usage pattern and the different bacterial subspecies. The species between species id 5 and 6 (red arrow) is Bacteroides fragilis NCTC 9343. The numbers from 1 to 64 refer to the bacterial genera. 1 Helicobacter pylori, 2 Acetobacter pasteurianus, 3 Bacillus amyloliquefaciens, 4 Bacillus subtilis, 5 Zymomonas mobilis, 6 Alteromonas macleodii, 7 Lactobacillus plantarum, 8 Lactobacillus casei, 9 Lactobacillus rhamnosus, 10 Coxiella burnetii, 11 Mannheimia haemolytica, 12 Shewanella baltica, 13 Vibrio cholerae, 14 Yersinia pestis, 15 Acinetobacter baumannii, 16 Haemophilus influenzae, 17 Enterococcus faecalis, 18 Listeria monocytogenes, 19 Bacillus anthracis, Bacillus cereus or Bacillus thuringiensis, 20 Staphylococcus aureus, 21 Lactococcus lactis, 22 Streptococcus agalactiae, 23 Legionella pneumophila, 24 Mycoplasma hyopneumoniae, 25 Chlamydia trachomatis, 26 Chlamydophila pneumoniae, 27 Chlamydophila psittaci, 28 Lactobacillus reuteri, 29 Streptococcus dysgalactiae, 30 Streptococcus pyogenes, 31 Streptococcus pneumoniae, 32 Streptococcus suis, 33 Borrelia burgdorferi, 34 Prochlorococcus marinus, 35 Clostridium botulinum, 36 Candidatus Kinetoplastibacterium, 37 Francisella tularensis, 38 Campylobacter jejuni, 39 Rickettsia prowazekii, 40 Rickettsia rickettsii, 41 Wolbachia endosymbiont, 42 Mycoplasma gallisepticum, 43 Mycoplasma hyorhinis, 44 Brucella melitensis, 45 Corynebacterium glutamicum, 46 Propionibacterium acnes, 47 Corynebacterium diphtheria, 48 Corynebacterium pseudotuberculosis, 49 Xylella fastidiosa, 50 Treponema pallidum, 51 Enterobacter cloacae, 52 Klebsiella pneumoniae, 53 *Escherichia coli*, 54 Salmonella enterica, 55 Neisseria meningitidis, 56 Burkholderia pseudomallei, 57 Bifidobacterium animalis, 58 Bifidobacterium longum, 59 Mycobacterium bovis, Mycobacterium canettii or Mycobacterium tuberculosis, 60 Rhodopseudomonas palustris, 61 Pseudomonas fluorescens or Pseudomonas aeruginosa, 62 Ralstonia solanacearum, 63 Pseudomonas putida, 64 Pseudomonas stutzeri.

The codon usage pattern of each subspecies was calculated, normalized and clustered (Fig. 1), and an evolutionary tree based on the codon usage patterns was constructed (Figs S2B and S4). The tree of those selected bacterial genomes in the Newick format is shown in Fig. S5. Nearly all of the subspecies within each species clustered into one group based on the codon usage patterns. Most of the species showed a higher usage frequency for the codons ATG, GAT and GAA than for other codons. However, the usage frequency for the codons CTA, AGG and CGA in these bacteria was lower than for other codons. Except for the six codons ATG, GAT, GAA, CTA, AGG and CGA, there was considerable deviation in codon usage patterns between bacteria belonging to different taxa. Bacterial strains within the same species had similar codon usage patterns. However, as shown in Figs S4 and S5, species within the same genus (*Bacillus*, *Lactobacillus*, *Mycoplasma* or *Corynebacterium*) showed different codon usage patterns. For example, as shown in Fig. S4, the codon usage pattern of *Bacillus amyloliquefaciens* was very similar to that of *B. subtilis* but differed greatly from those of *B. anthracis*, *B. cereus* and *B. thuringiensis*. Therefore, if a target gene is isolated from a different species or genus from the host used in a heterologous expression system, the gene may need to be optimized for the efficient expression in the host strain.

### Codon Usage Pattern of the Middle Amino Acid in short peptides

**Figure 2:**
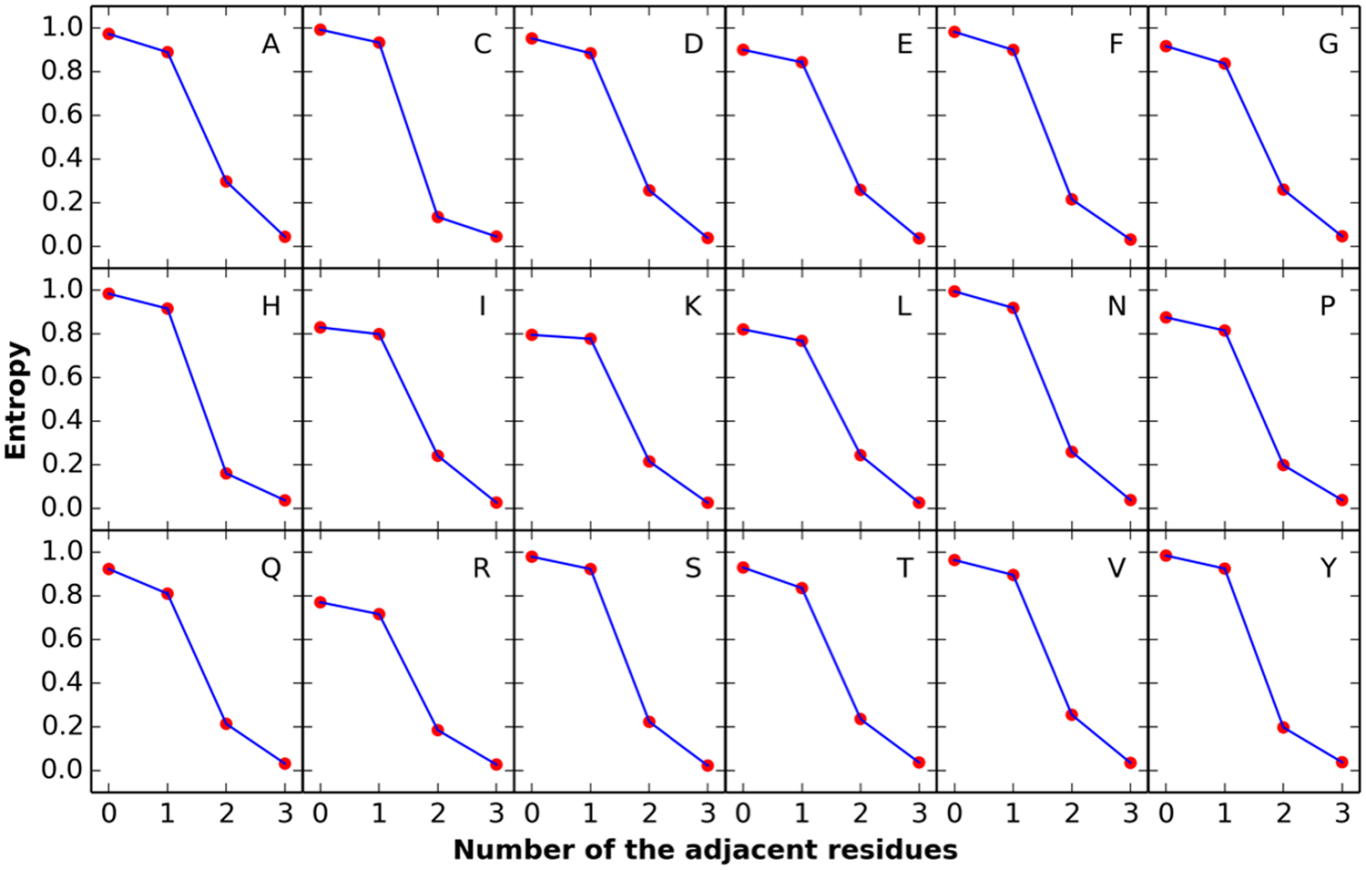
The entropy of the codon usage pattern of the middle amino acid with the different amino acid neighbors in *E. coli*. The x-axis represents the different number of the adjacent amino acids. The y-axis represents the average entropy of all codon usage pattern of the middle amino acid with corresponding adjacent amino acids. The data were calculated by the 65 genomes of *E. coli* (Table S1).

**Figure 3:**
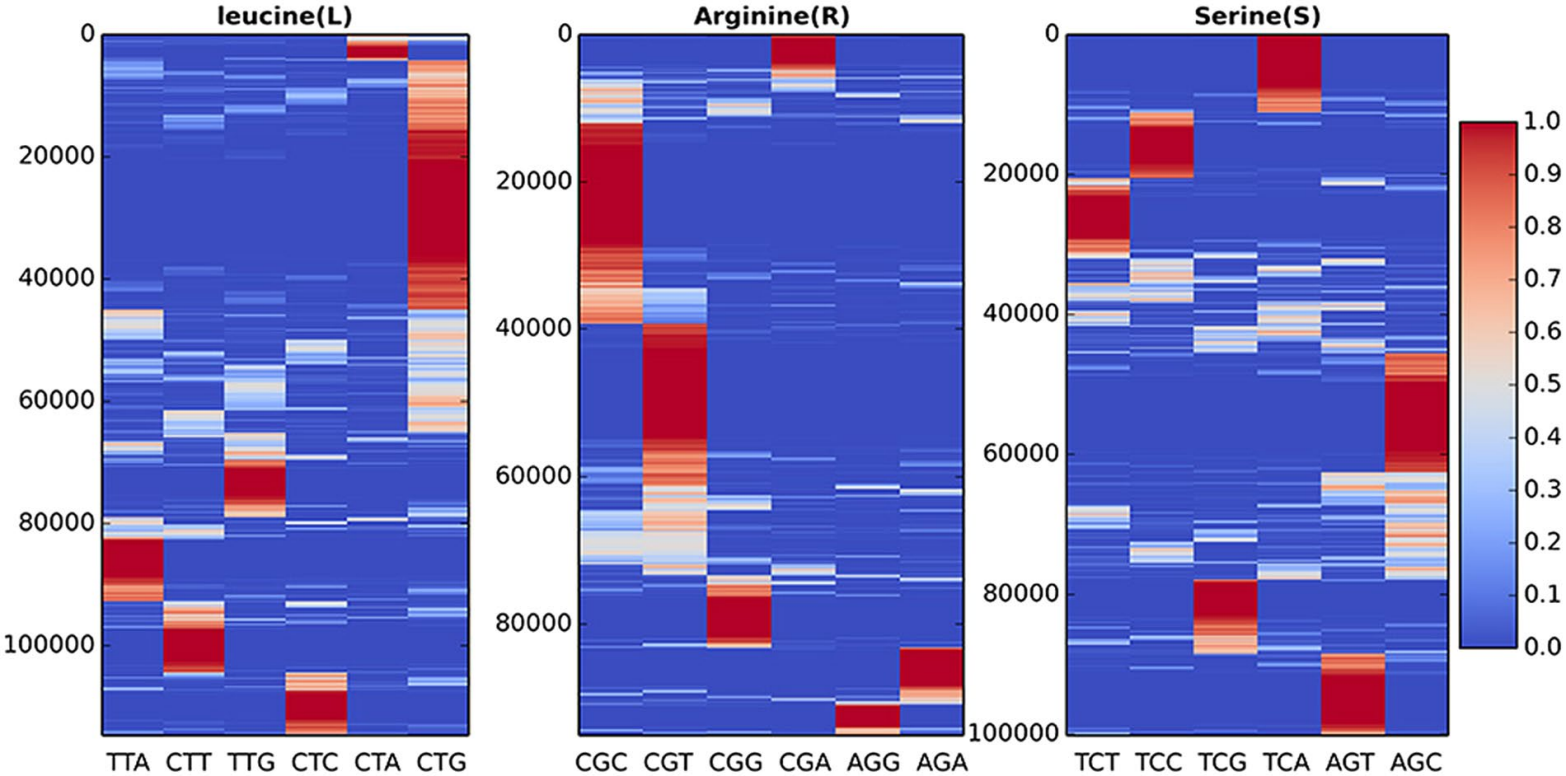
The codon usage patters of Leucine (L), Arginine (R) and Serine (S) in the specific fragment of *E. coli*. All genes of *E. coli* were divided into five-codon windows. The same amino acid fragments were merged and the codon usage bias of the middle amino acid (L, R and S) in the fragment was calculated. Each row represents the codon usage bias of the middle amino acids (L, R and S) in an amino acid fragment with five residues. Each column represents the codons to code the target amino acid. The color from blue to red represents the codon usage frequency of the codon.

All of the protein sequences encoded by the 65 genomes of *E. coli* (Table S1) were split into window sizes of one, three, five or seven amino acids. The same amino acid fragments from all of the genomes were merged, and the codon usage distribution of the middle amino acid in the fragment was calculated. The codon usage entropy of each amino acid was calculated, which represents the uncertainty of the codon selection of the amino acid. If the entropy of an amino acid is 1, the synonymous codon would be randomly selected. If the entropy is 0, however, only one specific codon can be selected for the amino acid. As shown in Fig. 2, the middle amino acid in the fragments with five or seven residues is likely to be coded by one specific codon, as the entropies for these fragments were significantly lower than those for fragments with one or three residues. As shown in Fig. 3, the codon of the middle residue in some fragments was determined by the peptide-dependent selection of the specific codon. For example, the rarest codon among the 61 codons in *E. coli* is AGG, which accounted for only 1.4‰ of the codons for arginine in the 65 *E. coli* genomes. There were 132 fragments containing the sequence GRRVA in the translated genes from the 65 *E. coli* genomes, and all of the 132 codons for the middle residue (arginine) were AGG. Therefore, the middle amino acid within a short peptide with length greater and equal than five amino acids usually used the specific codon by the peptide to code the residue. And the codon usage pattern of the short peptide will be used as the input vector of Presnycodon.

### Codon Prediction with the Machine-Learning Method

The *E. coli* genome dataset (Table S1) contains 65 genomes, 64 of which were selected to create a codon selection index (CSI) file. To eliminate the over-fit effect of the model, one of which (*E. coli* K12_MG1655) was excluded in the construction of the CSI file was used to evaluate performance of the method and. All of the proteins encoded by the 64 genomes of *E. coli* were split into window sizes of three, five or seven amino acids. The same amino acid fragments from all of the genomes were merged into one CSI vector, which contained the codon usage distribution for the middle amino acid and the average codon usage for each amino acid in the fragment. Thus, 8000, 1,686,761 and 4,366,175 CSI vectors were generated for the three-, five- and seven-amino acid *E. coli* fragments, respectively.

All of the translated protein sequences from *E. coli* K12_MG1655 were split into window sizes of three, five or seven amino acids and searched against the corresponding CSI files for *E. coli*. The entirely matched index record was used to generate the input vector to predict the codon selection of the middle amino acid. As there are 18 amino acids coded by multiple codons in nature, 18 classifiers for each window size were constructed to predict the codon selection of the target amino acid. A ten-fold cross validation was carried out to evaluate the performance of each classifier. As shown in Fig. S7, if every codon was predicted as the high-frequency-usage codon, the median prediction accuracy for each amino acid was ~54.3%. However, if the window size was five or seven amino acids, the median prediction accuracy of the 18 classifiers increased to 80.53 and 97.54%, respectively, as shown in Fig. S7.

**Figure 4:**
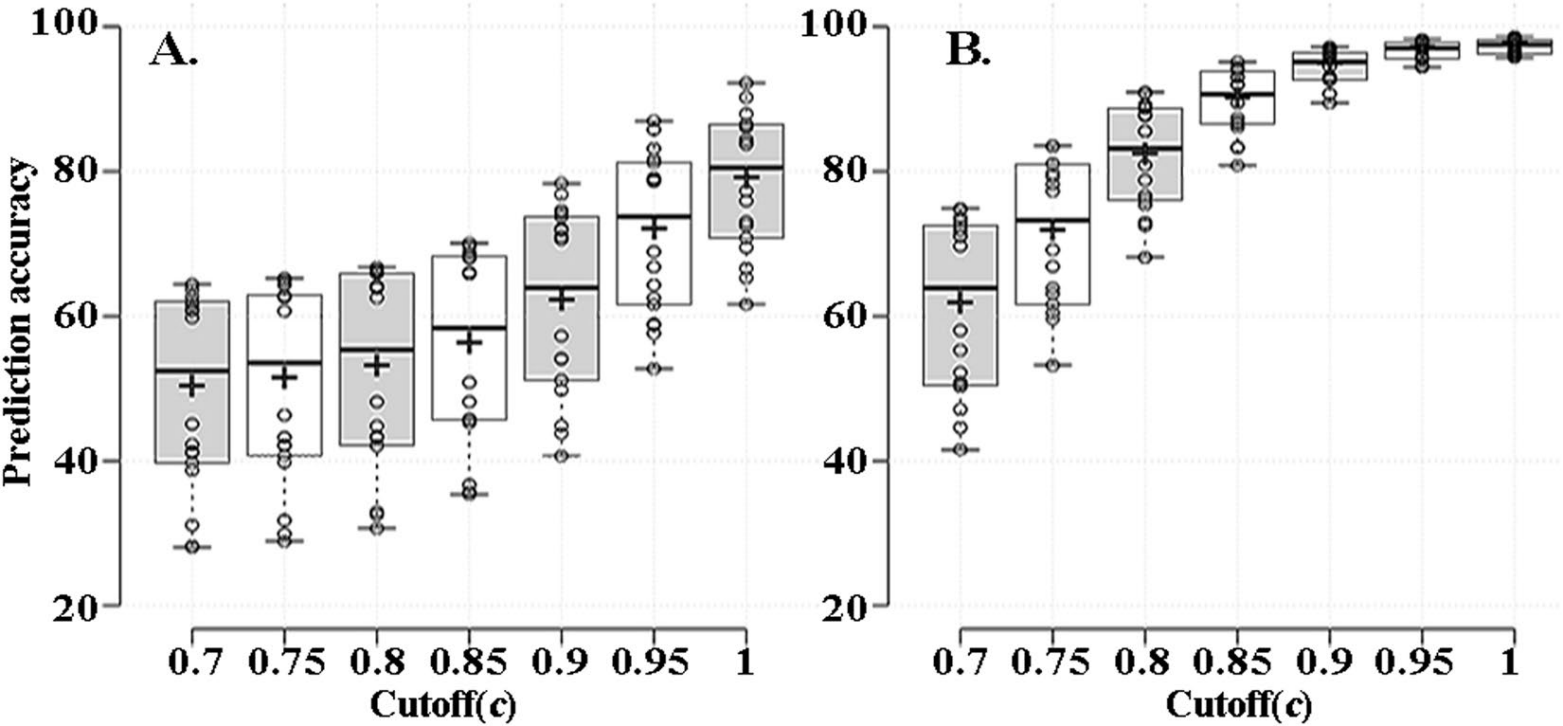
The prediction performance of the 18 classifiers for the 18 amino acids with different matched cutoff and window size (**A**) five amino acids, (**B**) seven amino acids) in *E. coli*. The x-axis is the matched percent and the y-axis is the prediction accuracy of the 18 classifiers. Each open circle represents the prediction accuracy with one of the 18 classifiers. The horizontal divisions (from top to bottom) in each box are the upper whisker, 3rd quartile, median, 1st quartile and lower whisker, respectively. The cross line in each box is the mean prediction accuracy of all 18 classifiers. All of the results were calculated based on a ten-fold cross validation.

Thus, if a window size of five or seven amino acids was selected, the classifier could obtain high accuracy. However, it was based on only 1,686,761 and 4,366,175 *E. coli* CSI values for the five- and seven-amino acid windows, which is only 52.7 and 0.3% of all of the possible values for five (20^5^ **= **3,200,000) and seven (20^7^ **= **1,280,000,000) amino acid windows. Here, we didn’t consider the longer window size than 7 amino acids, as the long window size contained more possible amino acid fragments. If the fragments being assessed were not in the index file, the target codon would not be predicted. To predict codon usage for the most fragments possible, the matched percent (*p, p = s/m*) for each fragment was assessed, which is the percent of matching between a calculated matched score (*s*) and expected maximal score (*m*) of the target fragment, as shown in Fig. S6. For a given cutoff (*c*), if the matched percent of multiple fragments from the CSI files was greater than the cutoff, the coding vector for the target codon is just the arithmetic average of all of the matched record vectors. The evaluation results for the different cutoffs are shown in Fig. 4 and Table S2. The classifier achieved high accuracy when the *c* was greater than 0.9 for a window size of five amino acids or greater than 0.8 for a window size of seven amino acids, which the AUCs (Area Under the receiver operating characteristic Curve) of most of those classifiers were great than 0.7 and 0.8, respectively.

The numbers of the fragments of five and seven amino acids in all 346 bacterial genomes were 2,758,946 and 66,114,871, respectively. If the cutoff (*c*) was set as 0.8, 99.3 and 63.8% of the five- and seven-amino acid fragments, respectively, could be predicted by the method. Therefore, based on this idea, most of the codons in a heterologous gene could be predicted by the method with the appropriate cutoff (*c*) and window size.

For the aim to predict the codon selection of a target gene, all predicting models with different window sizes (5 and 7 amino acids) and cutoff *c* (0.8, 0.85, 0.9, 0.95 and 1) were constructed. The models with window size of seven amino acids and big cutoff *c* have priority over the models with window size of five amino acids and small cutoff *c*, respectively. Based on this process, each amino acid was predicted the codon usage tendency by only one predicting model which the long window size and big cutoff *c* should be selected with priority. As a result, the gene sequence of a target protein was predicted from the amino acid sequence by the method except the first and last two codons of the gene. As the first 30 codons of a gene usually selected as the codons with the low-frequency-usage codons^5, 39^, the first two codons of the gene were selected as the low-frequency-usage codons. The last two codons usually select the high-frequency-usage codons, as they didn’t affect the expression of the target genes.

### Design of a Codon-Optimized Reporter Gene

**Figure 5:**
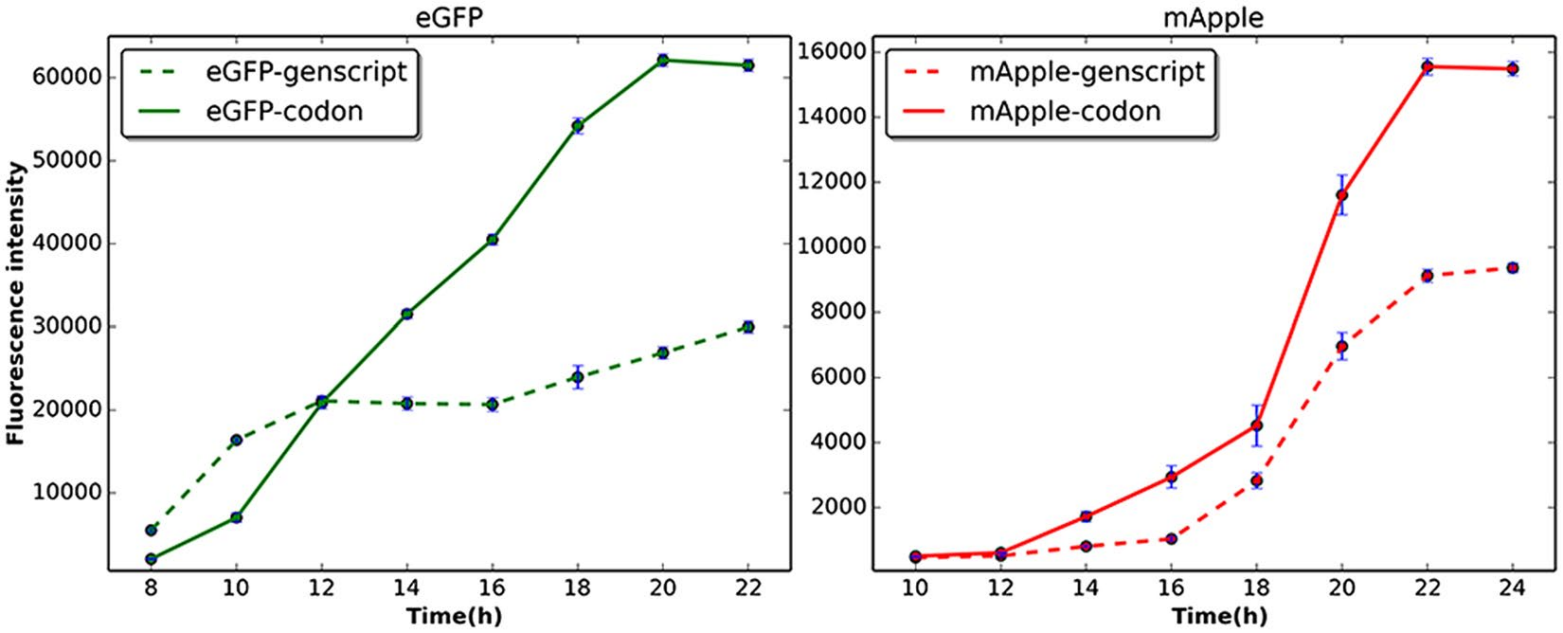
Fluorescence intensity of *E. coli* containing the reporter genes (egfp or mApple). The reporter genes (egfp-codon and mApple-codon) were designed based on the model in this study. The genes (egfp-genscript and mApple-genscript) were designed, in which most of the low-frequency-usage codons were changed to the high-frequency-usage codons of *E. coli* using GenScript software. The strain harboring the corresponding expression plasmid was grown in the auto-induction medium containing 50 μg/mL kanamycin. Data are averages of ten independent experiments. The error bars represent the standard error.

The two reporter genes (*egfp-codon* and *mApple-codon*) were designed using the classifiers described above. In addition, another two control genes (*egfp-genscript* and *mApple-genscript*) were designed, which mainly used the high-frequency-usage codons of *E. coli* designed by the GenScript software. The sequences of those genes are shown in Figs S8 and S9. The four genes (*egfp-codon*, *mApple-codon, egfp-genscript* and *mApple-genscript*) were expressed in the same *E. coli* expression system. The fluorescence intensity of eGFP and mApple expressed from eGFP-codon and mApple-codon was about 2.3- or 1.7- folds greater than that from eGFP-genscript and mApple-genscript (Fig. 5). In addition, as shown in SDS-PAGE (Fig. S10), the amount of the expressed proteins eGFP and mApple from eGFP-codon and mApple-codon was also higher than that from eGFP-genscript and mApple-genscript. As shown in the codon usage table for the four genes in Table S3, there are several low-frequency-usage codons in the designed genes (*egfp-codon* and *mApple-codon*), such as CTA, CGA and AGA. The designed reporter genes (*egfp-codon* and *mApple-codon*) are a combination of low- and high-frequency-usage codons. Therefore, the low-frequency-usage codons also make a positive contribution to the expression of the reporter genes, indicating that our method could be used to optimize target gene expression without changing the amino acid sequence of the resulting protein.

## Discussion

In this study, we proposed a machine learning based method, namely Presyncodon, to design the heterogenous gene with the optimal codon usage for expression. The studies on the two reporter genes (*egfp* and *mApple*) revealed that the low-frequency-usage codons (rare codons) also have the important roles for the functional and soluble expression of the target gene. Here, we also calculated the relation between the strength of relative codon bias (RCBS) and the protein abundance of the *E. coli* genes. As shown in Fig. S11, both the high and low abundant proteins in the cell contains the low- and high-frequency-usage codons. Therefore, the optimal codon for a heterogenous gene should be the appropriate combination between the low- and high-frequency-usage codons. The software Presyncodon was developed to find this interesting combination among synonymous codons for a heterogenous gene.

Therefore, Presyncodon is different from the other software programs such as Gene Designer^26^, Gene composer^27^ and OPTIMIZER^30^ which optimized the heterologous gene with the “preferred” synonymous codons of the expression host. In addition, it was also different to the “codon harmonization” method^35, 36^. As a result, Presyncodon did not need to know the information of the codon usage bias of the native host, and it could design any proteins based on the learned knowledge from the *E. coli* genome.

The key of Presyncodon is the codon usage pattern of a specific fragment in the CSI files, but only one residue, which was encouraged by the Google’s statistical machine translation (SMT) model. The parameters of the SMT model are derived from the analysis of bilingual text corpora. But the codon usage pattern of a specific fragment was learned from the entire genome database of *E. coli*. Then the predictive models for the expression host *E. coli* were constructed with the learned codon usage patterns. The modes for other important expression hosts, such as *Bacillus subtilis*, and *Pichia pastoris* will be developed in the future.

## Materials and Methods

### Plasmids and Bacterial Strains

*E. coli* BL21 (DE3) and plasmid pET-30a (+) were used as the expression host and the expression vector for the expression of recombinant proteins. The designed genes (*egfp-codon*, *mApple-codon*, *egfp-genscript* and *mApple-genscript*) were synthesized by GenScript Corporation (Nanjing, China) and inserted into the plasmid pET-30a(+) with the restriction enzymes (*Bam*HI and *Hin*dIII).

### Genome Dataset

The dataset of bacterial genomes with full annotation was downloaded from NCBI (ftp://ftp.ncbi.nlm.nih.gov/genomes/Bacteria/) on December 8, 2014. All of the selected subspecies are shown in Table S1.

### Phylogenetic Analyses

The 346 genomes in Table S1 were used to carry out the phylogenetic analyses. Those bacterial species had at least five sequenced subspecies except the bacterium *Bacteroides fragilis* NCTC 9343 which was selected as the out-group^38^. The 16 S rDNA sequences of the selected subspecies were collected from the Ribosomal Database Project^40^. All of the 16 S rDNA sequences were aligned with Clustalw^41^. The aligned 16 S rDNA sequences were exported in PHYLIP format for analysis using the PHYLIP set of programs, version 3.696^42^. Similarity matrices of the 16 S rDNA sequences were constructed with the F84 nucleotide substitution model^43, 44^ using the Dnadist program in PHYLIP. Phylogenetic trees were constructed with the neighbor-joining method with the Neighbor program in PHYLIP. The reliability of the neighbor-joining tree was estimated by bootstrap analysis using 1000 replication datasets generated by the program Seqboot in PHYLIP. For the codon usage trees, 2000 genes were randomly selected from the target genome file, and the Euclidean distances of the codon usage of the subspecies were calculated to construct the corresponding similarity matrices among all of the different subspecies. The 1000 similarity matrices were created by the random selection method. Then, the program Consense in PHYLIP read all of the constructed trees and generated a consensus tree. Trees were drawn and analyzed with the Dendroscope program, version 3.0^45^.

### Data Clustering and Visualization

All of the data in this study were clustered using the open-source software Cluster, version 3.0^46^, and the clustered data were visualized with TreeView, version 1.1.6r4^47^.

### Construction and Evaluation of the Codon Prediction Model

The *E. coli* genome dataset (Table S1) contains 65 genomes, 64 of which were selected to create a codon selection index (CSI), and one of which (*E. coli* K12_MG1655) was used to evaluate performance of the method. All proteins encoded by the 64 genomes of *E. coli* were split into window sizes of three, five or seven amino acids. The same amino acid fragments from all the genomes were merged into one CSI vector, which contained the codon usage distribution of the middle amino acid and the average codon usage for each amino acid in the fragment. The genome of *E. coli* MG1655 was used to train and evaluate the performance of the model. Every gene in the *E. coli* MG1655 was split into three-, five- or seven-codon windows. Here, the size of the codon window was defined as *w*. Every short nucleotide sequence was also translated as a short peptide, and then all of the short peptides were searched against the CSI file with the corresponding codon window size. As we wanted to predict the codon selection of the middle amino acid in the peptide, the middle amino acid of the matched peptide and input short peptide must be the same. The matched score (*s*) between the input peptide and the peptide in the CSI file could be calculated with a BLOSUM62 matrix^48^. The expected maximal score (*m*) of the input short peptide is the sum of the corresponding diagonal scores in BLOSUM62 matrix of the amino acids in the input peptide. A cutoff (*c*) could be defined to select the appropriate peptide in the CSI file. Therefore, if the percent (*p, p = s*/*m*) of the matched score (*s*) to the expected maximal score (*m*) is greater than cutoff *c*, the matched peptide in the CSI file would be selected to update the corresponding input vector. The final input vector of the short peptide is the arithmetic mean of all the possible matched peptides, and the weight of the matched peptide is the matched score(*s*). Therefore, if an appropriate cutoff *c* and window size *w* were defined, every codon in the gene except the first and last *w* codons could be represented by one vector, and all of the vector were collected as the input dataset to predict the codon selection in *E. coli*. As two (methionine and tryptophan) of the 20 amino acids are coded by only one codon, 18 models for each cutoff *c* and window size *w* of the *E. coli* MG1655 genomes were constructed to predict the codon selection with a random forest classifier^49^. The number of trees of the key parameter of the classifier for random forests is 1000. The average overall accuracy and the AUC (Area Under the receiver operating characteristic Curve) for each amino acid model with different parameters were used to evaluate the performance of the method, which was calculated based on a ten-fold cross validation. The AUC was calculated with the package of pROC^50^.

### Protein Expression and Purification

The reporter genes (*egfp-codon*, *mApple-codon*, *egfp-genscript* and *mApple-genscript*) were cloned into a pET30a(+) expression vector and overexpressed in *E. coli* strain BL21(DE3) pLys. Ten single colonies of the transformed *E. coli* carrying the reporter gene were cultured in liquid Luria-Bertani medium containing 50 μg/mL kanamycin at 30 °C overnight and then inoculated to fresh auto-induction medium (2:100 dilution) and incubated again at 30 °C with shaking at 750 rpm in incubator 1000 (Heidolph, Germany)^51^. The fluorescence intensity was measured at two-hour intervals using a SpectraMax M2 instrument (Molecular Devices, USA). The excitation and emission wavelengths were 484 and 507 nm and 568 and 592 nm, for eGFP and mApple, respectively^52^. The values shown are the averages of ten independent experiments.

### Relative codon bias

The strength of relative codon bias (RCBS) was calculated based on the equation in the references^53, 54^. The protein abundance data of the *E. coli* was retrieved from paxdB^55^.

### Method Availability

For non-commercial purposes, the code of the software Presyncodon can be downloaded from http://www.mobioinfor.cn/presyncodon.

### Data Availability

All data generated or analyzed during this study are included in this published article (and its Supplementary Information files).

## Electronic supplementary material

Supplementary Information 
