# Supplementary material for: Predicting synonymous codon usage and optimizing the heterologous gene for expression in E. coli
Source: Sci Rep. 2017 Aug 30;7:9926. doi: 10.1038/s41598-017-10546-0 (PMC5577221; doi:10.1038/s41598-017-10546-0)
Supplement: Supplementary file 1 — Supplementary Information [file 41598_2017_10546_MOESM1_ESM.pdf]

Predicting synonymous codon usage and optimizing the heterologous gene for expression in *E. coli*

Jian Tian[1], Yaru Yan[2,1], Qingxia Yue[3,1], Xiaoqing Liu[1], Xiaoyu Chu[1], Ningfeng Wu[1]*, Yunliu Fan[1]

1. Biotechnology Research Institute, Chinese Academy of Agricultural Sciences, Beijing 100081, China
2. College of Food Science and Technology, Agricultural University of Hebei , Baoding, HeBei Provice, 071001, China
3. Institute of Microbial Biotechnology, Jinan University, Guangzhou, Guangdong Province, 510632, China

*Corresponding author:

Ningfeng Wu, Tel.: 86-10-82109864. Fax: 86-10-82109844.

E-mail: wuningfeng@caas.cn

((((((((((((((((((((Streptococcaceae_Streptococcus_agalactiae_09mas018883_uid208674:1000.0,Streptococcaceae_Streptococcus_agalactiae_2603V_R_uid57943:1000.0)72:0.072,(Streptococcaceae_Streptococcus_agalactiae_GD201008_001_uid175780:1000.0,Strept
ococcaceae_Streptococcus_agalactiae_A909_uid57935:1000.0)63:0.063)61:0.061,Streptococcaceae_Streptococcus_agalactiae_ILRI005_uid208676:1000.0)100:0.1,(((((Streptococcaceae_Streptococcus_pyogenes_MGAS9429_uid58569:1000.0,Streptococcaceae_Strept
ococcus_pyogenes_A20_uid178106:1000.0)20:0.02,Streptococcaceae_Streptococcus_pyogenes_HSC5_uid212978:1000.0)26:0.026,Streptococcaceae_Streptococcus_pyogenes_Alab49_uid162171:1000.0)71:0.071,Streptococcaceae_Streptococcus_pyogenes_NZ131_ui
d59035:1000.0)100:0.1,((((Streptococcaceae_Streptococcus_dysgalactiae_AC_2713_uid178644:1000.0,Streptococcaceae_Streptococcus_dysgalactiae_ATCC_12394_uid161979:1000.0)82:0.082,Streptococcaceae_Streptococcus_dysgalactiae_RE378_uid176684:1000.
0)68:0.068,Streptococcaceae_Streptococcus_dysgalactiae_167_uid222822:1000.0)72:0.072,Streptococcaceae_Streptococcus_dysgalactiae_GGS_124_uid59103:1000.0)100:0.1)93:0.093)100:0.1,(Streptococcaceae_Streptococcus_suis_D12_uid162127:1000.0,(((Strepto
coccaceae_Streptococcus_suis_98HAH33_uid58665:1000.0,Streptococcaceae_Streptococcus_suis_A7_uid162111:1000.0)25:0.025,Streptococcaceae_Streptococcus_suis_05ZYH33_uid58663:1000.0)32:0.032,Streptococcaceae_Streptococcus_suis_BM407_uid59321:1
000.0)93:0.093)100:0.1)91:0.091,(((Streptococcaceae_Streptococcus_pneumoniae_A026_uid226114:1000.0,(Streptococcaceae_Streptococcus_pneumoniae_Taiwan19F_14_uid59119:1000.0,Streptococcaceae_Streptococcus_pneumoniae_TCH8431_19A_uid49735:100
0.0)38:0.038)82:0.082,Streptococcaceae_Streptococcus_pneumoniae_ATCC_700669_uid59287:1000.0)88:0.088,Streptococcaceae_Streptococcus_pneumoniae_TIGR4_uid57857:1000.0)100:0.1)100:0.1,(((Streptococcaceae_Lactococcus_lactis_Il1403_uid57671:1000.
0,(Streptococcaceae_Lactococcus_lactis_KF147_uid42831:1000.0,Streptococcaceae_Lactococcus_lactis_KLDS_4_0325_uid225028:1000.0)26:0.026)30:0.03,Streptococcaceae_Lactococcus_lactis_CV56_uid160253:1000.0)78:0.078,Streptococcaceae_Lactococcus_lac
tis_IO_1_uid192185:1000.0)100:0.1)100:0.1,(((((Enterococcaceae_Enterococcus_faecalis_OG1RF_uid54927:1000.0,(Enterococcaceae_Enterococcus_faecalis_62_uid159663:1000.0,Enterococcaceae_Enterococcus_faecalis_V583_uid57669:1000.0)56:0.056)91:0.091,
Enterococcaceae_Enterococcus_faecalis_D32_uid171261:1000.0)64:0.064,Enterococcaceae_Enterococcus_faecalis_Symbioflor_1_uid183342:1000.0)100:0.1,((((((((Bacillaceae_Bacillus_cereus_B4264_uid58757:1000.0,(Bacillaceae_Bacillus_cereus_03BB102_uid59
299:1000.0,((Bacillaceae_Bacillus_thuringiensis_HD_771_uid173374:1000.0,Bacillaceae_Bacillus_thuringiensis_Bt407_uid177931:1000.0)69:0.069,(Bacillaceae_Bacillus_thuringiensis_MC28_uid176369:1000.0,Bacillaceae_Bacillus_thuringiensis_BMB171_uid4913
5:1000.0)45:0.045)37:0.037)35:0.035)55:0.055,Bacillaceae_Bacillus_cereus_AH187_uid58753:1000.0)49:0.049,Bacillaceae_Bacillus_cereus_ATCC_10987_uid57673:1000.0)39:0.039,(((Bacillaceae_Bacillus_anthracis_H9401_uid162021:1000.0,(Bacillaceae_Bacillu
s_anthracis_CDC_684_uid59303:1000.0,Bacillaceae_Bacillus_cereus_biovar_anthracis_CI_uid50615:1000.0)35:0.035)33:0.033,(Bacillaceae_Bacillus_anthracis_A0248_uid59385:1000.0,Bacillaceae_Bacillus_anthracis_Ames_Ancestor_uid58083:1000.0)96:0.096)29:
0.029,Bacillaceae_Bacillus_thuringiensis_Al_Hakam_uid58795:1000.0)29:0.029)43:0.043,Bacillaceae_Bacillus_anthracis_Sterne_uid58091:1000.0)100:0.1,(((((Bacillaceae_Bacillus_subtilis_168_uid57675:1000.0,Bacillaceae_Bacillus_subtilis_BSn5_uid62463:1000.0
)53:0.053,Bacillaceae_Bacillus_subtilis_BSP1_uid184010:1000.0)62:0.062,Bacillaceae_Bacillus_subtilis_PY79_uid229877:1000.0)69:0.069,Bacillaceae_Bacillus_subtilis_natto_BEST195_uid183001:1000.0)85:0.085,(Bacillaceae_Bacillus_amyloliquefaciens_DSM_7
_uid53535:1000.0,((Bacillaceae_Bacillus_amyloliquefaciens_CC178_uid226115:1000.0,Bacillaceae_Bacillus_amyloliquefaciens_IT_45_uid181617:1000.0)97:0.097,(Bacillaceae_Bacillus_amyloliquefaciens_plantarum_AS43_3_uid183682:1000.0,Bacillaceae_Bacillu
s_amyloliquefaciens_FZB42_uid58271:1000.0)84:0.084)99:0.099)79:0.079)100:0.1)78:0.078,((Staphylococcaceae_Staphylococcus_aureus_55_2053_uid55909:1000.0,((Staphylococcaceae_Staphylococcus_aureus_08BA02176_uid175257:1000.0,Staphylococcaceae_S
taphylococcus_aureus_6850_uid217772:1000.0)40:0.04,Staphylococcaceae_Staphylococcus_aureus_04_02981_uid161969:1000.0)40:0.04)79:0.079,Staphylococcaceae_Staphylococcus_aureus_11819_97_uid159981:1000.0)100:0.1)100:0.1,(((Listeriaceae_Listeria_mo
nocytogenes_10403S_uid54461:1000.0,(Listeriaceae_Listeria_monocytogenes_07PF0776_uid162185:1000.0,Listeriaceae_Listeria_monocytogenes_Clip80459_uid59317:1000.0)24:0.024)43:0.043,Listeriaceae_Listeria_monocytogenes_ATCC_19117_uid175109:1000
.0)78:0.078,Listeriaceae_Listeria_monocytogenes_Finland_1998_uid54443:1000.0)100:0.1)90:0.09)98:0.098,((((((Lactobacillaceae_Lactobacillus_reuteri_TD1_uid213089:1000.0,Lactobacillaceae_Lactobacillus_reuteri_JCM_1112_uid58875:1000.0)61:0.061,Lactoba
cillaceae_Lactobacillus_reuteri_I5007_uid208677:1000.0)50:0.05,Lactobacillaceae_Lactobacillus_reuteri_SD2112_uid55357:1000.0)86:0.086,Lactobacillaceae_Lactobacillus_reuteri_DSM_20016_uid58471:1000.0)100:0.1,((Lactobacillaceae_Lactobacillus_rhamnosu
s_GG_uid59313:1000.0,(((Lactobacillaceae_Lactobacillus_rhamnosus_LOCK900_uid210957:1000.0,Lactobacillaceae_Lactobacillus_rhamnosus_Lc_705_uid59315:1000.0)45:0.045,Lactobacillaceae_Lactobacillus_rhamnosus_ATCC_8530_uid162169:1000.0)78:0.07
8,Lactobacillaceae_Lactobacillus_rhamnosus_LOCK908_uid210958:1000.0)79:0.079)100:0.1,(((Lactobacillaceae_Lactobacillus_casei_Zhang_uid50673:1000.0,(Lactobacillaceae_Lactobacillus_casei_W56_uid178736:1000.0,Lactobacillaceae_Lactobacillus_casei_AT
CC_334_uid57985:1000.0)90:0.09)53:0.053,Lactobacillaceae_Lactobacillus_casei_LOCK919_uid210959:1000.0)53:0.053,Lactobacillaceae_Lactobacillus_casei_BL23_uid59237:1000.0)100:0.1)100:0.1)93:0.093,(Lactobacillaceae_Lactobacillus_plantarum_ZJ316_ui
d188689:1000.0,((Lactobacillaceae_Lactobacillus_plantarum_JDM1_uid59361:1000.0,(Lactobacillaceae_Lactobacillus_plantarum_P8_uid203333:1000.0,Lactobacillaceae_Lactobacillus_plantarum_ST_III_uid53537:1000.0)99:0.099)74:0.074,Lactobacillaceae_Lactob
acillus_plantarum_WCFS1_uid62911:1000.0)64:0.064)100:0.1)100:0.1)68:0.068)100:0.1,((Clostridiaceae_Clostridium_botulinum_H04402_065_uid162091:1000.0,Clostridiaceae_Clostridium_botulinum_A_ATCC_3502_uid61579:1000.0)100:0.1,((Clostridiaceae_Cl
ostridium_botulinum_F_230613_uid159513:1000.0,Clostridiaceae_Clostridium_botulinum_F_Langeland_uid58929:1000.0)96:0.096,Clostridiaceae_Clostridium_botulinum_A_ATCC_19397_uid58927:1000.0)68:0.068)100:0.1)100:0.1,(((((Mycoplasmataceae_Mycopl
asma_hyopneumoniae_232_uid58205:1000.0,Mycoplasmataceae_Mycoplasma_hyopneumoniae_168_uid162053:1000.0)95:0.095,(Mycoplasmataceae_Mycoplasma_hyopneumoniae_7448_uid58039:1000.0,Mycoplasmataceae_Mycoplasma_hyopneumoniae_7422_uid
212968:1000.0)91:0.091)64:0.064,Mycoplasmataceae_Mycoplasma_hyopneumoniae_J_uid58059:1000.0)100:0.1,((Mycoplasmataceae_Mycoplasma_hyorhinis_DBS_1050_uid228933:1000.0,Mycoplasmataceae_Mycoplasma_hyorhinis_MCLD_uid162087:1000.0)98:
0.098,(Mycoplasmataceae_Mycoplasma_hyorhinis_GDL_1_uid87003:1000.0,(Mycoplasmataceae_Mycoplasma_hyorhinis_HUB_1_uid51695:1000.0,Mycoplasmataceae_Mycoplasma_hyorhinis_SK76_uid181997:1000.0)51:0.051)69:0.069)100:0.1)100:0.1,((Mycopla
smataceae_Mycoplasma_gallisepticum_CA06_uid172630:1000.0,Mycoplasmataceae_Mycoplasma_gallisepticum_R_low_uid57993:1000.0)63:0.063,(Mycoplasmataceae_Mycoplasma_gallisepticum_WI01_2001_043_13_2P_uid172628:1000.0,(Mycoplasmataceae_M
ycoplasma_gallisepticum_VA94_uid172624:1000.0,Mycoplasmataceae_Mycoplasma_gallisepticum_NC06_uid172629:1000.0)50:0.05)96:0.096)100:0.1)100:0.1)56:0.056,(((Prochlorococcaceae_Prochlorococcus_marinus_pastoris_CCMP1986_uid57761:1000.0,Proch
lorococcaceae_Prochlorococcus_marinus_AS9601_uid58307:1000.0)100:0.1,(Prochlorococcaceae_Prochlorococcus_marinus_CCMP1375_uid57995:1000.0,Prochlorococcaceae_Prochlorococcus_marinus_MIT_9211_uid58309:1000.0)64:0.064)56:0.056,Prochlorococ
caceae_Prochlorococcus_marinus_NATL2A_uid58359:1000.0)100:0.1)56:0.056,(((((((Corynebacteriaceae_Corynebacterium_glutamicum_R_uid58897:1000.0,(Corynebacteriaceae_Corynebacterium_glutamicum_SCgG1_uid207285:1000.0,Corynebacteriaceae_Coryn
ebacterium_glutamicum_SCgG2_uid207286:1000.0)85:0.085)77:0.077,Corynebacteriaceae_Corynebacterium_glutamicum_ATCC_13032_uid193708:1000.0)81:0.081,Corynebacteriaceae_Corynebacterium_glutamicum_MB001_uid214793:1000.0)100:0.1,(((Coryneb
acteriaceae_Corynebacterium_diphtheriae_VA01_uid84305:1000.0,Corynebacteriaceae_Corynebacterium_diphtheriae_INCA_402_uid83605:1000.0)100:0.1,((Corynebacteriaceae_Corynebacterium_diphtheriae_NCTC_13129_uid57691:1000.0,Corynebacteriaceae_Co
rynebacterium_diphtheriae_PW8_uid84303:1000.0)88:0.088,Corynebacteriaceae_Corynebacterium_diphtheriae_CDCE_8392_uid84295:1000.0)100:0.1)100:0.1,((Corynebacteriaceae_Corynebacterium_pseudotuberculosis_316_uid89381:1000.0,((Corynebacteriaceae_
Corynebacterium_pseudotuberculosis_1002_uid159677:1000.0,Corynebacteriaceae_Corynebacterium_pseudotuberculosis_267_uid162175:1000.0)41:0.041,Corynebacteriaceae_Corynebacterium_pseudotuberculosis_C231_uid159675:1000.0)96:0.096)90:0.09,Coryneb
acteriaceae_Corynebacterium_pseudotuberculosis_CIP_52_97_uid159667:1000.0)100:0.1)100:0.1)82:0.082,(((((((((Mycobacteriaceae_Mycobacterium_bovis_BCG_Mexico_uid86889:1000.0,Mycobacteriaceae_Mycobacterium_tuberculosis_CTRI_2_uid161997:1000.
0)11:0.011,(Mycobacteriaceae_Mycobacterium_canettii_CIPT_140070010_uid184828:1000.0,Mycobacteriaceae_Mycobacterium_bovis_BCG_Tokyo_172_uid59281:1000.0)11:0.011)10:0.010,((Mycobacteriaceae_Mycobacterium_canettii_CIPT_140070008_uid1848
32:1000.0,Mycobacteriaceae_Mycobacterium_bovis_BCG_Pasteur_1173P2_uid58781:1000.0)12:0.012,Mycobacteriaceae_Mycobacterium_tuberculosis_Beijing_NITR203_uid197218:1000.0)10:0.010)10:0.010,(Mycobacteriaceae_Mycobacterium_bovis_BCG_Korea
_1168P_uid189029:1000.0,Mycobacteriaceae_Mycobacterium_tuberculosis_CCDC5079_uid161943:1000.0)12:0.012)11:0.011,Mycobacteriaceae_Mycobacterium_tuberculosis_Erdman__ATCC_35801_uid193763:1000.0)15:0.015,Mycobacteriaceae_Mycobacterium_
tuberculosis_EAI5_NITR206_uid202218:1000.0)55:0.055,Mycobacteriaceae_Mycobacterium_bovis_AF2122_97_uid57695:1000.0)63:0.063,(Mycobacteriaceae_Mycobacterium_canettii_CIPT_140010059_uid70731:1000.0,Mycobacteriaceae_Mycobacterium_canettii
_CIPT_140060008_uid184829:1000.0)72:0.072)78:0.078,Mycobacteriaceae_Mycobacterium_canettii_CIPT_140070017_uid184830:1000.0)100:0.1)100:0.1,((Propionibacteriaceae_Propionibacterium_acnes_SK137_uid48071:1000.0,Propionibacteriaceae_Propionibact
erium_acnes_HL096PA1_uid198524:1000.0)72:0.072,(Propionibacteriaceae_Propionibacterium_acnes_6609_uid162137:1000.0,(Propionibacteriaceae_Propionibacterium_acnes_ATCC_11828_uid162177:1000.0,Propionibacteriaceae_Propionibacterium_acnes_KPA1
71202_uid58101:1000.0)52:0.052)60:0.06)100:0.1)100:0.1,((Bifidobacteriaceae_Bifidobacterium_animalis_ATCC_25527_uid162513:1000.0,(((Bifidobacteriaceae_Bifidobacterium_animalis_ATCC_27673_uid222803:1000.0,Bifidobacteriaceae_Bifidobacterium_ani
malis_CNCM_I_2494_uid158869:1000.0)41:0.041,Bifidobacteriaceae_Bifidobacterium_animalis_DSM_10140_uid59357:1000.0)100:0.1,Bifidobacteriaceae_Bifidobacterium_animalis_AD011_uid58911:1000.0)100:0.1)100:0.1,((Bifidobacteriaceae_Bifidobacterium_
longum_BBMN68_uid60163:1000.0,((Bifidobacteriaceae_Bifidobacterium_longum_infantis_157F_uid62693:1000.0,Bifidobacteriaceae_Bifidobacterium_longum_KACC_91563_uid158861:1000.0)25:0.025,Bifidobacteriaceae_Bifidobacterium_longum_F8_uid19718
4:1000.0)30:0.03)82:0.082,Bifidobacteriaceae_Bifidobacterium_longum_DJO10A_uid58833:1000.0)100:0.1)100:0.1)100:0.1)67:0.067,((((((((Chlamydiaceae_Chlamydophila_pneumoniae_TW_183_uid57997:1000.0,Chlamydiaceae_Chlamydophila_pneumoniae_AR3
9_uid57809:1000.0)51:0.051,Chlamydiaceae_Chlamydophila_pneumoniae_J138_uid57829:1000.0)61:0.061,Chlamydiaceae_Chlamydophila_pneumoniae_CWL029_uid57811:1000.0)100:0.1,Chlamydiaceae_Chlamydophila_pneumoniae_LPCoLN_uid159529:1000.0)
100:0.1,(((Chlamydiaceae_Chlamydophila_psittaci_C19_98_uid159523:1000.0,(Chlamydiaceae_Chlamydophila_psittaci_6BC_uid63621:1000.0,Chlamydiaceae_Chlamydophila_psittaci_01DC11_uid159527:1000.0)27:0.027)33:0.033,Chlamydiaceae_Chlamydophila_
psittaci_08DC60_uid159525:1000.0)98:0.098,Chlamydiaceae_Chlamydophila_psittaci_Mat116_uid189026:1000.0)100:0.1)92:0.092,((Chlamydiaceae_Chlamydia_trachomatis_434_Bu_uid61633:1000.0,(Chlamydiaceae_Chlamydia_trachomatis_Sweden2_uid161995:
1000.0,Chlamydiaceae_Chlamydia_trachomatis_IU824_uid193712:1000.0)100:0.1)88:0.088,(Chlamydiaceae_Chlamydia_trachomatis_B_Jali20_OT_uid59351:1000.0,Chlamydiaceae_Chlamydia_trachomatis_A2497_uid159993:1000.0)100:0.1)100:0.1)100:0.1,((Spiro
chaetaceae_Treponema_pallidum_Mexico_A_uid176920:1000.0,((Spirochaetaceae_Treponema_pallidum_pertenue_SamoaD_uid87069:1000.0,(Spirochaetaceae_Treponema_pallidum_SS14_uid58977:1000.0,Spirochaetaceae_Treponema_pallidum_Chicago_uid15954
3:1000.0)19:0.019)21:0.021,Spirochaetaceae_Treponema_pallidum_Fribourg_Blanc_uid201428:1000.0)30:0.03)100:0.1,((Spirochaetaceae_Borrelia_burgdorferi_ZS7_uid59429:1000.0,((Spirochaetaceae_Borrelia_burgdorferi_B31_uid57581:1000.0,Spirochaetaceae_B
orrelia_burgdorferi_JD1_uid161197:1000.0)52:0.052,Spirochaetaceae_Borrelia_burgdorferi_CA382_uid214794:1000.0)83:0.083)61:0.061,Spirochaetaceae_Borrelia_burgdorferi_N40_uid161241:1000.0)100:0.1)100:0.1)49:0.049,Bacteroidaceae_Bacteroides_fragilis_
NCTC_9343_uid57639:1000.0)29:0.029)50:0.05,(((Helicobacteraceae_Helicobacter_pylori_HPAG1_uid58517:1000.0,(Helicobacteraceae_Helicobacter_pylori_India7_uid161149:1000.0,(Helicobacteraceae_Helicobacter_pylori_2017_uid161151:1000.0,Helicobactera
ceae_Helicobacter_pylori_Gambia94_24_uid159493:1000.0)100:0.1)48:0.048)77:0.077,Helicobacteraceae_Helicobacter_pylori_26695_uid57787:1000.0)100:0.1,(Campylobacteraceae_Campylobacter_jejuni_doylei_269_97_uid58671:1000.0,(((Campylobacteraceae_C
ampylobacter_jejuni_81116_uid58771:1000.0,Campylobacteraceae_Campylobacter_jejuni_4031_uid222817:1000.0)66:0.066,Campylobacteraceae_Campylobacter_jejuni_81_176_uid58503:1000.0)58:0.058,Campylobacteraceae_Campylobacter_jejuni_00_2544_uid2
19326:1000.0)42:0.042)100:0.1)100:0.1)100:0.1,((((((((((((Enterobacteriaceae_Salmonella_enterica_serovar_Javiana_CFSAN001992_uid190101:1000.0,Enterobacteriaceae_Salmonella_enterica_serovar_Newport_SL254_uid58831:1000.0)67:0.067,((Enterobacteriacea
e_Salmonella_enterica_arizonae_serovar_62_z4_z23_uid58191:1000.0,Enterobacteriaceae_Salmonella_enterica_serovar_Heidelberg_SL476_uid58973:1000.0)71:0.071,Enterobacteriaceae_Salmonella_enterica_serovar_Agona_24249_uid230614:1000.0)100:0.1)100:0
.1,((((Enterobacteriaceae_Enterobacter_cloacae_EcWSU1_uid80739:1000.0,Enterobacteriaceae_Enterobacter_cloacae_ENHKU01_uid172463:1000.0)100:0.1,(Enterobacteriaceae_Enterobacter_cloacae_dissolvens_SDM_uid168997:1000.0,Enterobacteriaceae_Enterob
acter_cloacae_ATCC_13047_uid48363:1000.0)83:0.083)88:0.088,Enterobacteriaceae_Enterobacter_cloacae_NCTC_9394_uid197202:1000.0)65:0.065,((((Enterobacteriaceae_Klebsiella_pneumoniae_1084_uid174151:1000.0,Enterobacteriaceae_Klebsiella_pneumonia
e_JM45_uid215235:1000.0)71:0.071,Enterobacteriaceae_Klebsiella_pneumoniae_HS11286_uid84387:1000.0)58:0.058,Enterobacteriaceae_Klebsiella_pneumoniae_CG43_uid223021:1000.0)67:0.067,Enterobacteriaceae_Klebsiella_pneumoniae_KCTC_2242_uid1621
47:1000.0)100:0.1)77:0.077)65:0.065,(((Enterobacteriaceae_Escherichia_coli_BL21_Gold_DE3_pLysS_AG_uid59245:1000.0,Enterobacteriaceae_Escherichia_coli_B_REL606_uid58803:1000.0)100:0.1,Enterobacteriaceae_Escherichia_coli_K_12_substr_MG1655_ui
d57779:1000.0)70:0.07,(Enterobacteriaceae_Escherichia_coli_ATCC_8739_uid58783:1000.0,Enterobacteriaceae_Escherichia_coli_ETEC_H10407_uid161993:1000.0)100:0.1)100:0.1)100:0.1,(((Enterobacteriaceae_Yersinia_pestis_CO92_uid57621:1000.0,(Enterobact
eriaceae_Yersinia_pestis_biovar_Medievalis_Harbin_35_uid158537:1000.0,Enterobacteriaceae_Yersinia_pestis_D106004_uid158071:1000.0)98:0.098)65:0.065,Enterobacteriaceae_Yersinia_pestis_A1122_uid158119:1000.0)92:0.092,Enterobacteriaceae_Yersinia_pes
tis_Angola_uid58485:1000.0)100:0.1)100:0.1,((((Vibrionaceae_Vibrio_cholerae_IEC224_uid89389:1000.0,Vibrionaceae_Vibrio_cholerae_LMA3984_4_uid159541:1000.0)73:0.073,Vibrionaceae_Vibrio_cholerae_O1_biovar_El_Tor_N16961_uid57623:1000.0)52:0.0
52,Vibrionaceae_Vibrio_cholerae_O395_uid58425:1000.0)68:0.068,Vibrionaceae_Vibrio_cholerae_MJ_1236_uid59387:1000.0)100:0.1)100:0.1,(((Shewanellaceae_Shewanella_baltica_BA175_uid52601:1000.0,Shewanellaceae_Shewanella_baltica_OS117_uid162025
:1000.0)100:0.1,(Shewanellaceae_Shewanella_baltica_OS195_uid58261:1000.0,Shewanellaceae_Shewanella_baltica_OS185_uid58743:1000.0)77:0.077)95:0.095,Shewanellaceae_Shewanella_baltica_OS155_uid58259:1000.0)100:0.1)76:0.076,((((Pasteurellaceae_Ma
nnheimia_haemolytica_D153_uid212303:1000.0,Pasteurellaceae_Mannheimia_haemolytica_USDA_ARS_USMARC_183_uid195458:1000.0)21:0.021,(Pasteurellaceae_Mannheimia_haemolytica_USMARC_2286_uid213228:1000.0,Pasteurellaceae_Mannheimia_hae
molytica_M42548_uid198769:1000.0)21:0.021)31:0.031,Pasteurellaceae_Mannheimia_haemolytica_D174_uid212305:1000.0)100:0.1,((((Pasteurellaceae_Haemophilus_influenzae_PittGG_uid58593:1000.0,Pasteurellaceae_Haemophilus_influenzae_86_028NP_uid58
093:1000.0)75:0.075,Pasteurellaceae_Haemophilus_influenzae_10810_uid86647:1000.0)100:0.1,Pasteurellaceae_Haemophilus_influenzae_F3047_uid62097:1000.0)100:0.1,Pasteurellaceae_Haemophilus_influenzae_KR494_uid219323:1000.0)100:0.1)100:0.1)90:0.09
,((Alteromonadaceae_Alteromonas_macleodii_Black_Sea_11_uid176365:1000.0,Alteromonadaceae_Alteromonas_macleodii_Balearic_Sea_AD45_uid176366:1000.0)100:0.1,((Alteromonadaceae_Alteromonas_macleodii_Deep_ecotype_uid58251:1000.0,Alteromonad
aceae_Alteromonas_macleodii_English_Channel_615_uid210781:1000.0)95:0.095,Alteromonadaceae_Alteromonas_macleodii_Ionian_Sea_U4_uid210780:1000.0)82:0.082)100:0.1)100:0.1,(((((((((Pseudomonadaceae_Pseudomonas_putida_KT2440_uid57843:1000.0,
Pseudomonadaceae_Pseudomonas_putida_BIRD_1_uid162055:1000.0)57:0.057,Pseudomonadaceae_Pseudomonas_putida_H8234_uid208673:1000.0)100:0.1,(Pseudomonadaceae_Pseudomonas_putida_NBRC_14164_uid208670:1000.0,Pseudomonadaceae_Pseudom
onas_putida_W619_uid58651:1000.0)83:0.083)100:0.1,Pseudomonadaceae_Pseudomonas_fluorescens_Pf0_1_uid57591:1000.0)52:0.052,(Pseudomonadaceae_Pseudomonas_fluorescens_F113_uid87037:1000.0,((Pseudomonadaceae_Pseudomonas_fluorescens_Pf_5_
uid57937:1000.0,Pseudomonadaceae_Pseudomonas_fluorescens_A506_uid165185:1000.0)100:0.1,Pseudomonadaceae_Pseudomonas_fluorescens_SBW25_uid158693:1000.0)73:0.073)98:0.098)43:0.043,(((((Pseudomonadaceae_Pseudomonas_aeruginosa_B136_33_u
id196598:1000.0,Pseudomonadaceae_Pseudomonas_aeruginosa_c7447m_uid219358:1000.0)31:0.031,Pseudomonadaceae_Pseudomonas_aeruginosa_LESB58_uid59275:1000.0)31:0.031,Pseudomonadaceae_Pseudomonas_aeruginosa_NCGM2_S1_uid162173:1000.0)
79:0.079,Pseudomonadaceae_Pseudomonas_aeruginosa_DK2_uid168996:1000.0)100:0.1,(((Pseudomonadaceae_Pseudomonas_stutzeri_RCH2_uid184342:1000.0,Pseudomonadaceae_Pseudomonas_stutzeri_CCUG_29243_uid168379:1000.0)51:0.051,Pseudomonadac
eae_Pseudomonas_stutzeri_DSM_10701_uid170940:1000.0)65:0.065,(Pseudomonadaceae_Pseudomonas_stutzeri_A1501_uid58641:1000.0,Pseudomonadaceae_Pseudomonas_stutzeri_ATCC_17588__LMG_11199_uid68749:1000.0)100:0.1)100:0.1)59:0.059)100:0.1,
((((Moraxellaceae_Acinetobacter_baumannii_1656_2_uid158677:1000.0,Moraxellaceae_Acinetobacter_baumannii_ATCC_17978_uid58731:1000.0)38:0.038,Moraxellaceae_Acinetobacter_baumannii_ACICU_uid58765:1000.0)81:0.081,Moraxellaceae_Acinetobacter
_baumannii_AYE_uid61637:1000.0)77:0.077,Moraxellaceae_Acinetobacter_baumannii_AB0057_uid59083:1000.0)100:0.1)72:0.072,(Legionellaceae_Legionella_pneumophila_Paris_uid58211:1000.0,(((Legionellaceae_Legionella_pneumophila_LPE509_uid193710:1
000.0,Legionellaceae_Legionella_pneumophila_Thunder_Bay_uid206517:1000.0)52:0.052,Legionellaceae_Legionella_pneumophila_Philadelphia_1_uid57609:1000.0)88:0.088,Legionellaceae_Legionella_pneumophila_Lorraine_uid170535:1000.0)82:0.082)100:0.1)5
6:0.056,((((Coxiellaceae_Coxiella_burnetii_Dugway_5J108_111_uid58629:1000.0,Coxiellaceae_Coxiella_burnetii_RSA_493_uid57631:1000.0)62:0.062,Coxiellaceae_Coxiella_burnetii_CbuG_Q212_uid58893:1000.0)77:0.077,Coxiellaceae_Coxiella_burnetii_RSA_3
31_uid58637:1000.0)70:0.07,Coxiellaceae_Coxiella_burnetii_CbuK_Q154_uid58895:1000.0)100:0.1)64:0.064)75:0.075,(((((((Burkholderiaceae_Ralstonia_solanacearum_FQY_4_f_uid194089:1000.0,Burkholderiaceae_Ralstonia_solanacearum_GMI1000_uid57593:1
000.0)100:0.1,Burkholderiaceae_Ralstonia_solanacearum_PSI07_uid50539:1000.0)55:0.055,(Burkholderiaceae_Ralstonia_solanacearum_CMR15_uid227773:1000.0,Burkholderiaceae_Ralstonia_solanacearum_CFBP2957_uid50545:1000.0)86:0.086)100:0.1,((Burkhol
deriaceae_Burkholderia_pseudomallei_MSHR346_uid55259:1000.0,(Burkholderiaceae_Burkholderia_pseudomallei_1106a_uid58515:1000.0,Burkholderiaceae_Burkholderia_pseudomallei_BPC006_uid174460:1000.0)44:0.044)64:0.064,(Burkholderiaceae_Burkholde
ria_pseudomallei_NCTC_13179_uid226109:1000.0,Burkholderiaceae_Burkholderia_pseudomallei_668_uid58389:1000.0)79:0.079)100:0.1)100:0.1,(((Alcaligenaceae_Candidatus_Kinetoplastibacterium_crithidii_uid189749:1000.0,Alcaligenaceae_Candidatus_Kinetop
lastibacterium_ATCC_30255_uid183630:1000.0)99:0.099,Alcaligenaceae_Candidatus_Kinetoplastibacterium_desouzaii_uid189750:1000.0)100:0.1,(Alcaligenaceae_Candidatus_Kinetoplastibacterium_TCC290E_uid189840:1000.0,Alcaligenaceae_Candidatus_Kineto
plastibacterium_blastocrithidii_uid183629:1000.0)100:0.1)100:0.1)100:0.1,((((Neisseriaceae_Neisseria_meningitidis_053442_uid58587:1000.0,Neisseriaceae_Neisseria_meningitidis_H44_76_uid162083:1000.0)90:0.09,Neisseriaceae_Neisseria_meningitidis_G2136_u
id162085:1000.0)53:0.053,Neisseriaceae_Neisseria_meningitidis_FAM18_uid57825:1000.0)69:0.069,Neisseriaceae_Neisseria_meningitidis_alpha710_uid161971:1000.0)100:0.1)100:0.1,(((Xanthomonadaceae_Xylella_fastidiosa_Temecula1_uid57869:1000.0,(Xantho
monadaceae_Xylella_fastidiosa_GB514_uid162023:1000.0,Xanthomonadaceae_Xylella_fastidiosa_M23_uid58809:1000.0)55:0.055)100:0.1,Xanthomonadaceae_Xylella_fastidiosa_M12_uid58763:1000.0)45:0.045,Xanthomonadaceae_Xylella_fastidiosa_9a5c_uid578
49:1000.0)100:0.1)92:0.092)65:0.065,(Francisellaceae_Francisella_tularensis_SCHU_S4_uid57589:1000.0,(((Francisellaceae_Francisella_tularensis_holarctica_F92_uid181998:1000.0,Francisellaceae_Francisella_tularensis_mediasiatica_FSC147_uid58939:1000.0)99:
0.099,Francisellaceae_Francisella_tularensis_FSC198_uid58693:1000.0)57:0.057,Francisellaceae_Francisella_tularensis_NE061598_uid161973:1000.0)100:0.1)100:0.1)100:0.1)100:0.1,((((Anaplasmataceae_Wolbachia_endosymbiont_Drosophila_simulans_wHa_uid1
98768:1000.0,Anaplasmataceae_Wolbachia_endosymbiont_Drosophila_melanogaster_uid57851:1000.0)100:0.1,Anaplasmataceae_Wolbachia_endosymbiont_TRS_of_Brugia_malayi_uid58107:1000.0)70:0.07,(Anaplasmataceae_Wolbachia_endosymbiont_Drosophila
_simulans_wNo_uid198767:1000.0,Anaplasmataceae_Wolbachia_endosymbiont_Culex_quinquefasciatus_Pel_uid61645:1000.0)100:0.1)100:0.1,((((Rickettsiaceae_Rickettsia_rickettsii_Sheila_Smith_uid58027:1000.0,Rickettsiaceae_Rickettsia_rickettsii_Hauke_uid86
659:1000.0)19:0.019,(Rickettsiaceae_Rickettsia_rickettsii_Brazil_uid88069:1000.0,Rickettsiaceae_Rickettsia_rickettsii_Arizona_uid86655:1000.0)21:0.021)31:0.031,Rickettsiaceae_Rickettsia_rickettsii_Colombia_uid86653:1000.0)100:0.1,(((Rickettsiaceae_Rickettsia
_prowazekii_Breinl_uid196851:1000.0,(Rickettsiaceae_Rickettsia_prowazekii_GvV257_uid158051:1000.0,Rickettsiaceae_Rickettsia_prowazekii_BuV67_CWPP_uid158063:1000.0)21:0.021)20:0.02,Rickettsiaceae_Rickettsia_prowazekii_Dachau_uid158057:1000.0)
31:0.031,Rickettsiaceae_Rickettsia_prowazekii_Chernikova_uid158053:1000.0)100:0.1)100:0.1)100:0.1)71:0.071,((((Bradyrhizobiaceae_Rhodopseudomonas_palustris_DX_1_uid43327:1000.0,(Bradyrhizobiaceae_Rhodopseudomonas_palustris_CGA009_uid62901:10
00.0,Bradyrhizobiaceae_Rhodopseudomonas_palustris_TIE_1_uid58995:1000.0)100:0.1)100:0.1,(Bradyrhizobiaceae_Rhodopseudomonas_palustris_BisA53_uid58445:1000.0,Bradyrhizobiaceae_Rhodopseudomonas_palustris_HaA2_uid58439:1000.0)99:0.099)100:0.
1,((Brucellaceae_Brucella_melitensis_bv_1_16M_uid57735:1000.0,Brucellaceae_Brucella_melitensis_NI_uid158853:1000.0)75:0.075,((Brucellaceae_Brucella_melitensis_M5_90_uid158855:1000.0,Brucellaceae_Brucella_melitensis_M28_uid158857:1000.0)38:0.038
,Brucellaceae_Brucella_melitensis_ATCC_23457_uid59241:1000.0)93:0.093)100:0.1)98:0.098,((((Sphingomonadaceae_Zymomonas_mobilis_ATCC_10988_uid55403:1000.0,Sphingomonadaceae_Zymomonas_mobilis_NCIMB_11163_uid41019:1000.0)55:0.055,Sp
hingomonadaceae_Zymomonas_mobilis_CP4_NRRL_B_14023_uid229874:1000.0)47:0.047,Sphingomonadaceae_Zymomonas_mobilis_ATCC_29191_uid170612:1000.0)100:0.1,Sphingomonadaceae_Zymomonas_mobilis_pomaceae_ATCC_29192_uid68445:1000.0
)100:0.1)100:0.1)100:0.1,Acetobacteraceae_Acetobacter_pasteurianus_386B_uid214433:1000.0)31:0.031,Acetobacteraceae_Acetobacter_pasteurianus_IFO_3283_07_uid158381:1000.0)21:0.021,Acetobacteraceae_Acetobacter_pasteurianus_IFO_3283_03_uid158373:
1000.0)21:0.021,Acetobacteraceae_Acetobacter_pasteurianus_IFO_3283_01_uid59279:1000.0)100:0.1,Acetobacteraceae_Acetobacter_pasteurianus_IFO_3283_12_uid158379:1000.0);}

Figure S1. The 16S rDNA tree of the bacterial with the Newick format.

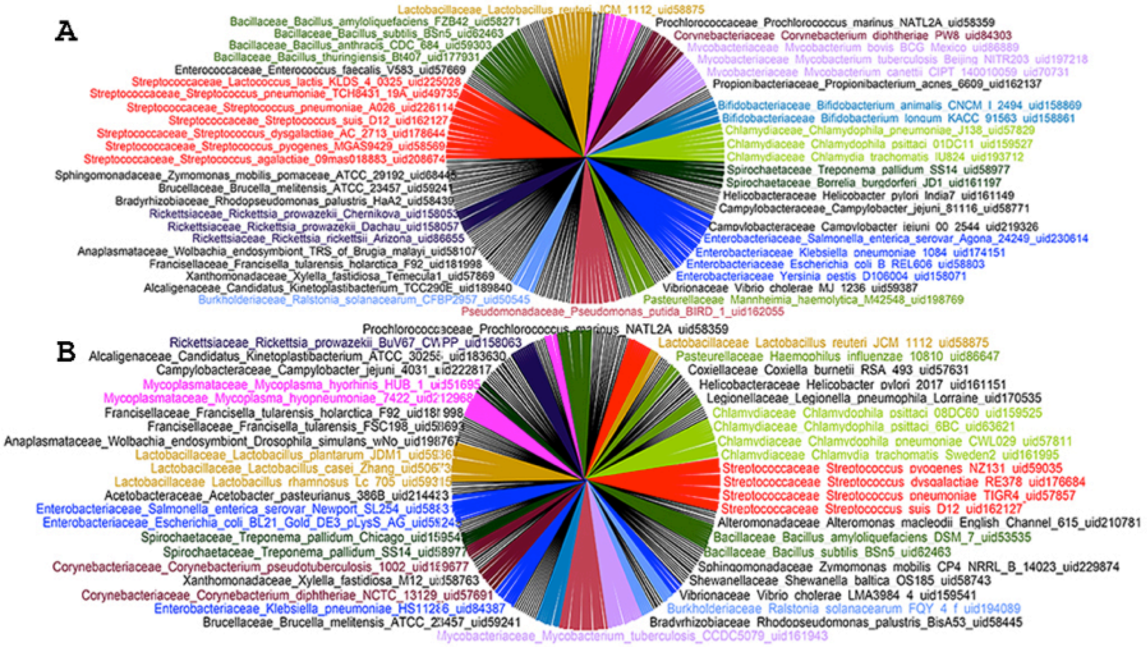

Figure S2. The evolutionary trees of the selected bacterial genomes. Panel A: phylogenetic trees based on the 16S rDNA; Panel B:Codon usage pattern. The bacterial family with at least 2 genus are shown in different colors, *Spirochaetaceae*, Deep Fir, *Enterobacteriaceae*, Blue Ribbon, *Bifidobacteriaceae*, Lochmara, *Rickettsiaceae*, Tolopea, *Bacillaceae*, Verdun Green, *Pasteurellaceae*, Limeade, *Burkholderiaceae*, Malibu, *Corynebacteriaceae*, Siren, *Chlamydiaceae*, Pistachio, *Pseudomonadaceae*, Fuzzy Wuzzy Brown, *Lactobacillaceae*, Buddha Gold, *Mycobacteriaceae*, Mauve, *Streptococcaceae*, Red, *Mycoplasmataceae*, Magenta. And the color of other families was shown in black.

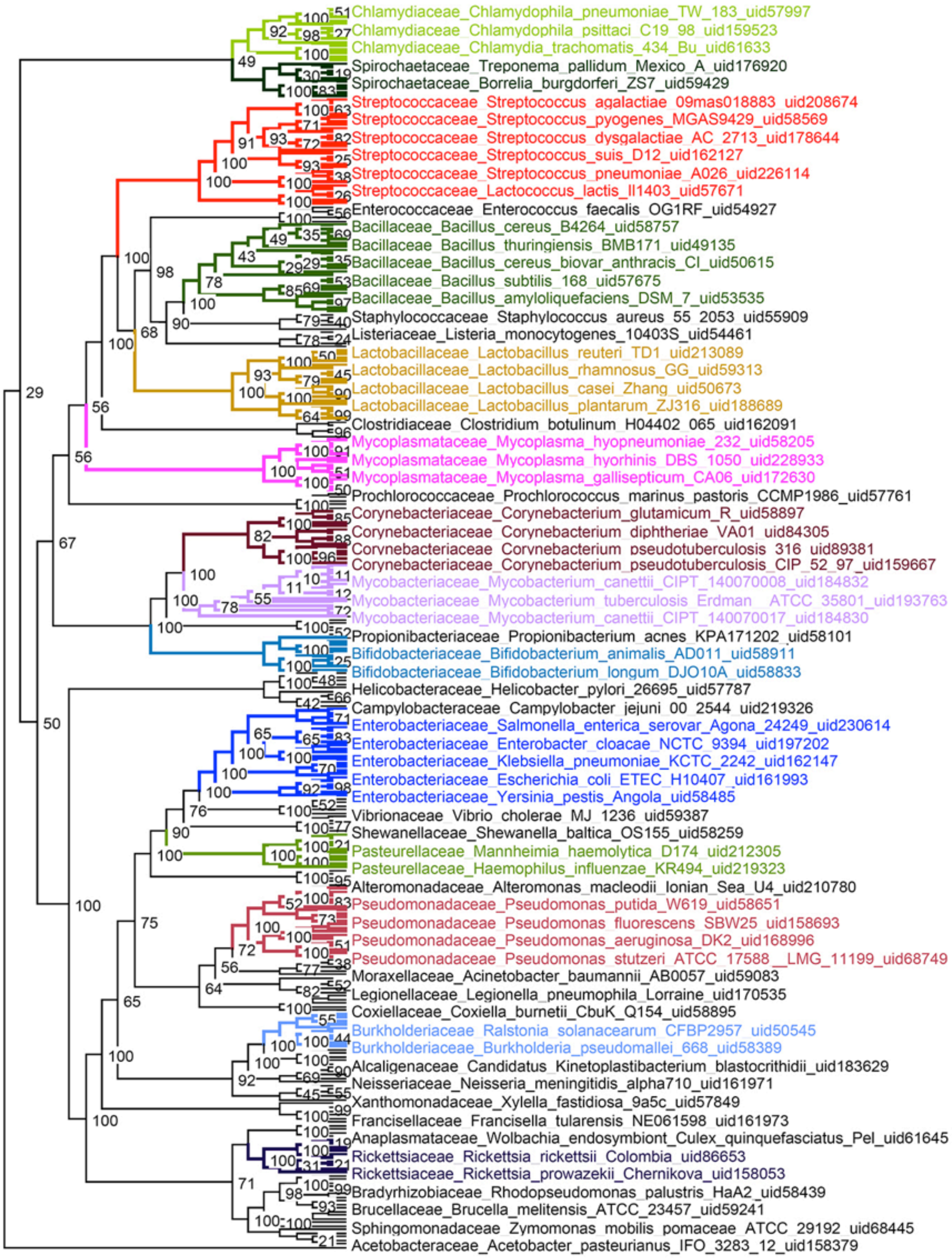

Figure S3. The evolutionary trees of the selected bacterial genomes based on the 16S rDNA. The color scheme was same to the figure S2.

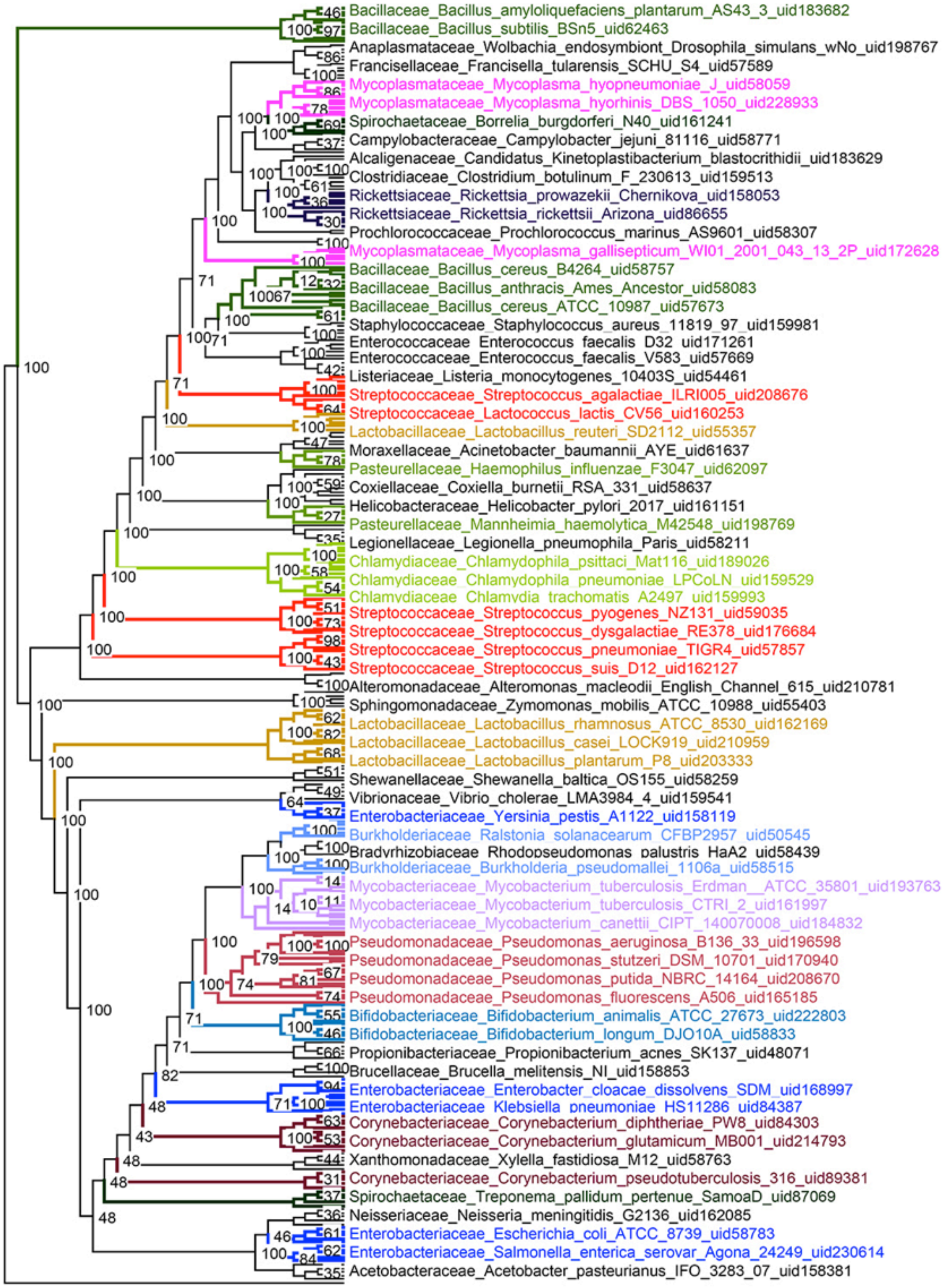

Figure S4. The evolutionary trees of the selected bacterial genomes based on the Codon usage pattern. The color scheme was same to the figure S2.

(((((((((((Lactobacillaceae_Lactobacillus_rhamnosus_Lc_705_uid59315:1000.0,(Lactobacillaceae_Lactobacillus_rhamnosus_LOCK908_uid210958:1000.0,(Lactobacillaceae_Lactobacillus_rhamnosus_LOCK900_uid210957:1000.0,Lactobacillaceae_Lactobacillus_rhamnosus_ATCC_8530_uid162169:1000.0)52:0.052)62:0.062)53:0.053,Lactobacillaceae_Lactobacillus_rhamnosus_GG_uid59313:1000.0)100:0.1,((((Lactobacillaceae_Lactobacillus_casei_Zhang_uid50673:1000.0,Lactobacillaceae_Lactobacillus_casei_ATCC_334_uid57985:1000.0)92:0.092,Lactobacillaceae_Lactobacillus_casei_BL23_uid59237:1000.0)82:0.082,Lactobacillaceae_Lactobacillus_casei_LOCK919_uid210959:1000.0)93:0.093,Lactobacillaceae_Lactobacillus_casei_W56_uid178736:1000.0)100:0.1)100:0.1,((((Lactobacillaceae_Lactobacillus_plantarum_JDM1_uid59361:1000.0,Lactobacillaceae_Lactobacillus_plantarum_ST_III_uid53537:1000.0)77:0.077,Lactobacillaceae_Lactobacillus_plantarum_ZJ316_uid188689:1000.0)68:0.068,Lactobacillaceae_Lactobacillus_plantarum_P8_uid203333:1000.0)55:0.055,Lactobacillaceae_Lactobacillus_plantarum_WCFS1_uid62911:1000.0)100:0.1)100:0.1,(((((((((((((((((Anaplasmataceae_Wolbachia_endosymbiont_Drosophila_simulans_wNo_uid198767:1000.0,Anaplasmataceae_Wolbachia_endosymbiont_Culex_quinquefasciatus_Pel_uid61645:1000.0)100:0.1,(Anaplasmataceae_Wolbachia_endosymbiont_TRS_of_Brugia_malayi_uid58107:1000.0,(Anaplasmataceae_Wolbachia_endosymbiont_Drosophila_simulans_wHa_uid198768:1000.0,Anaplasmataceae_Wolbachia_endosymbiont_Drosophila_melanogaster_uid57851:1000.0)100:0.1)86:0.086)100:0.1,(((Francisellaceae_Francisella_tularensis_SCHU_S4_uid57589:1000.0,Francisellaceae_Francisella_tularensis_FSC198_uid58693:1000.0)56:0.056,(Francisellaceae_Francisella_tularensis_holarctica_F92_uid181998:1000.0,Francisellaceae_Francisella_tularensis_NE061598_uid161973:1000.0)74:0.074)65:0.065,Francisellaceae_Francisella_tularensis_mediasiatica_FSC147_uid58939:1000.0)100:0.1)100:0.1,((((((Mycoplasmataceae_Mycoplasma_hyopneumoniae_J_uid58059:1000.0,((Mycoplasmataceae_Mycoplasma_hyopneumoniae_232_uid58205:1000.0,Mycoplasmataceae_Mycoplasma_hyopneumoniae_7448_uid58039:1000.0)69:0.069,Mycoplasmataceae_Mycoplasma_hyopneumoniae_7422_uid212968:1000.0)86:0.086)91:0.091,Mycoplasmataceae_Mycoplasma_hyopneumoniae_168_uid162053:1000.0)100:0.1,((((Mycoplasmataceae_Mycoplasma_hyorhinis_DBS_1050_uid228933:1000.0,Mycoplasmataceae_Mycoplasma_hyorhinis_MCLD_uid162087:1000.0)86:0.086,Mycoplasmataceae_Mycoplasma_hyorhinis_GDL_1_uid87003:1000.0)93:0.093,Mycoplasmataceae_Mycoplasma_hyorhinis_SK76_uid181997:1000.0)78:0.078,Mycoplasmataceae_Mycoplasma_hyorhinis_HUB_1_uid51695:1000.0)100:0.1)100:0.1,((((Spirochaetaceae_Borrelia_burgdorferi_N40_uid161241:1000.0,Spirochaetaceae_Borrelia_burgdorferi_ZS7_uid59429:1000.0)91:0.091,Spirochaetaceae_Borrelia_burgdorferi_B31_uid57581:1000.0)69:0.069,Spirochaetaceae_Borrelia_burgdorferi_JD1_uid161197:1000.0)100:0.1,Spirochaetaceae_Borrelia_burgdorferi_CA382_uid214794:1000.0)100:0.1)100:0.1,((((Campylobacteraceae_Campylobacter_jejuni_81116_uid58771:1000.0,Campylobacteraceae_Campylobacter_jejuni_4031_uid222817:1000.0)58:0.058,Campylobacteraceae_Campylobacter_jejuni_00_2544_uid219326:1000.0)37:0.037,Campylobacteraceae_Campylobacter_jejuni_81_176_uid58503:1000.0)100:0.1,Campylobacteraceae_Campylobacter_jejuni_doylei_269_97_uid58671:1000.0)100:0.1)100:0.1,((((Alcaligenaceae_Candidatus_Kinetoplastibacterium_blastocrithidii_uid183629:1000.0,(Alcaligenaceae_Candidatus_Kinetoplastibacterium_TCC290E_uid189840:1000.0,((Alcaligenaceae_Candidatus_Kinetoplastibacterium_crithidii_uid189749:1000.0,Alcaligenaceae_Candidatus_Kinetoplastibacterium_ATCC_30255_uid183630:1000.0)100:0.1,Alcaligenaceae_Candidatus_Kinetoplastibacterium_desouzaii_uid189750:1000.0)100:0.1)100:0.1)100:0.1,(Clostridiaceae_Clostridium_botulinum_F_230613_uid159513:1000.0,((Clostridiaceae_Clostridium_botulinum_H04402_065_uid162091:1000.0,(Clostridiaceae_Clostridium_botulinum_A_ATCC_3502_uid61579:1000.0,Clostridiaceae_Clostridium_botulinum_A_ATCC_19397_uid58927:1000.0)48:0.048)49:0.049,Clostridiaceae_Clostridium_botulinum_F_Langeland_uid58929:1000.0)61:0.061)100:0.1)100:0.1,(((((Rickettsiaceae_Rickettsia_prowazekii_Chernikova_uid158053:1000.0,Rickettsiaceae_Rickettsia_prowazekii_GvV257_uid158051:1000.0)34:0.034,Rickettsiaceae_Rickettsia_prowazekii_BuV67_CWPP_uid158063:1000.0)30:0.03,Rickettsiaceae_Rickettsia_prowazekii_Dachau_uid158057:1000.0)36:0.036,Rickettsiaceae_Rickettsia_prowazekii_Breinl_uid196851:1000.0)100:0.1,(Rickettsiaceae_Rickettsia_rickettsii_Arizona_uid86655:1000.0,((Rickettsiaceae_Rickettsia_rickettsii_Hauke_uid86659:1000.0,Rickettsiaceae_Rickettsia_rickettsii_Sheila_Smith_uid58027:1000.0)21:0.021,(Rickettsiaceae_Rickettsia_rickettsii_Colombia_uid86653:1000.0,Rickettsiaceae_Rickettsia_rickettsii_Brazil_uid88069:1000.0)26:0.026)30:0.03)100:0.1)100:0.1)100:0.1,(Prochlorococcaceae_Prochlorococcus_marinus_AS9601_uid58307:1000.0,Prochlorococcaceae_Prochlorococcus_marinus_pastoris_CCMP1986_uid57761:1000.0)100:0.1)100:0.1)100:0.1)100:0.1,((Prochlorococcaceae_Prochlorococcus_marinus_MIT_9211_uid58309:1000.0,Prochlorococcaceae_Prochlorococcus_marinus_CCMP1375_uid57995:1000.0)100:0.1,Prochlorococcaceae_Prochlorococcus_marinus_NATL2A_uid58359:1000.0)100:0.1)100:0.1,((((Mycoplasmataceae_Mycoplasma_gallisepticum_WI01_2001_043_13_2P_uid172628:1000.0,Mycoplasmataceae_Mycoplasma_gallisepticum_CA06_uid172630:1000.0)52:0.052,Mycoplasmataceae_Mycoplasma_gallisepticum_NC06_uid172629:1000.0)60:0.06,Mycoplasmataceae_Mycoplasma_gallisepticum_R_low_uid57993:1000.0)62:0.062,Mycoplasmataceae_Mycoplasma_gallisepticum_VA94_uid172624:1000.0)100:0.1)100:0.1,(((((Bacillaceae_Bacillus_cereus_B4264_uid58757:1000.0,((((Bacillaceae_Bacillus_thuringiensis_Al_Hakam_uid58795:1000.0,Bacillaceae_Bacillus_anthracis_H9401_uid162021:1000.0)17:0.017,(((Bacillaceae_Bacillus_anthracis_A0248_uid59385:1000.0,Bacillaceae_Bacillus_anthracis_Sterne_uid58091:1000.0)45:0.045,Bacillaceae_Bacillus_anthracis_Ames_Ancestor_uid58083:1000.0)32:0.032,Bacillaceae_Bacillus_anthracis_CDC_684_uid59303:1000.0)14:0.014)12:0.012,(Bacillaceae_Bacillus_cereus_03BB102_uid59299:1000.0,Bacillaceae_Bacillus_cereus_biovar_anthracis_CI_uid50615:1000.0)20:0.02)13:0.013,(Bacillaceae_Bacillus_cereus_AH187_uid58753:1000.0,Bacillaceae_Bacillus_cereus_ATCC_10987_uid57673:1000.0)26:0.026)67:0.067)52:0.052,Bacillaceae_Bacillus_thuringiensis_BMB171_uid49135:1000.0)100:0.1,(Bacillaceae_Bacillus_thuringiensis_Bt407_uid177931:1000.0,(Bacillaceae_Bacillus_thuringiensis_HD_771_uid173374:1000.0,Bacillaceae_Bacillus_thuringiensis_MC28_uid176369:1000.0)89:0.089)61:0.061)100:0.1,(((Staphylococcaceae_Staphylococcus_aureus_11819_97_uid159981:1000.0,Staphylococcaceae_Staphylococcus_aureus_04_02981_uid161969:1000.0)30:0.03,(Staphylococcaceae_Staphylococcus_aureus_6850_uid217772:1000.0,Staphylococcaceae_Staphylococcus_aureus_55_2053_uid55909:1000.0)38:0.038)32:0.032,Staphylococcaceae_Staphylococcus_aureus_08BA02176_uid175257:1000.0)100:0.1)100:0.1,((((Enterococcaceae_Enterococcus_faecalis_D32_uid171261:1000.0,(Enterococcaceae_Enterococcus_faecalis_Symbioflor_1_uid183342:1000.0,Enterococcaceae_Enterococcus_faecalis_OG1RF_uid54927:1000.0)100:0.1)96:0.096,Enterococcaceae_Enterococcus_faecalis_62_uid159663:1000.0)100:0.1,Enterococcaceae_Enterococcus_faecalis_V583_uid57669:1000.0)100:0.1,(Listeriaceae_Listeria_monocytogenes_ATCC_19117_uid175109:1000.0,((Listeriaceae_Listeria_monocytogenes_07PF0776_uid162185:1000.0,Listeriaceae_Listeria_monocytogenes_Clip80459_uid59317:1000.0)49:0.049,(Listeriaceae_Listeria_monocytogenes_Finland_1998_uid54443:1000.0,Listeriaceae_Listeria_monocytogenes_10403S_uid54461:1000.0)34:0.034)42:0.042)100:0.1)71:0.071)71:0.071)71:0.071,((((Streptococcaceae_Streptococcus_agalactiae_09mas018883_uid208674:1000.0,Streptococcaceae_Streptococcus_agalactiae_2603V_R_uid57943:1000.0)32:0.032,(Streptococcaceae_Streptococcus_agalactiae_GD201008_001_uid175780:1000.0,Streptococcaceae_Streptococcus_agalactiae_A909_uid57935:1000.0)47:0.047)62:0.062,Streptococcaceae_Streptococcus_agalactiae_ILRI005_uid208676:1000.0)100:0.1,(Streptococcaceae_Lactococcus_lactis_KLDS_4_0325_uid225028:1000.0,(Streptococcaceae_Lactococcus_lactis_KF147_uid42831:1000.0,((Streptococcaceae_Lactococcus_lactis_IO_1_uid192185:1000.0,Streptococcaceae_Lactococcus_lactis_Il1403_uid57671:1000.0)81:0.081,Streptococcaceae_Lactococcus_lactis_CV56_uid160253:1000.0)64:0.064)64:0.064)100:0.1)100:0.1)100:0.1,(((Lactobacillaceae_Lactobacillus_reuteri_I5007_uid208677:1000.0,(Lactobacillaceae_Lactobacillus_reuteri_JCM_1112_uid58875:1000.0,Lactobacillaceae_Lactobacillus_reuteri_DSM_20016_uid58471:1000.0)98:0.098)93:0.093,Lactobacillaceae_Lactobacillus_reuteri_TD1_uid213089:1000.0)100:0.1,Lactobacillaceae_Lactobacillus_reuteri_SD2112_uid55357:1000.0)100:0.1)71:0.071,(((((Moraxellaceae_Acinetobacter_baumannii_ATCC_17978_uid58731:1000.0,Moraxellaceae_Acinetobacter_baumannii_AB0057_uid59083:1000.0)58:0.058,Moraxellaceae_Acinetobacter_baumannii_1656_2_uid158677:1000.0)39:0.039,Moraxellaceae_Acinetobacter_baumannii_ACICU_uid58765:1000.0)47:0.047,Moraxellaceae_Acinetobacter_baumannii_AYE_uid61637:1000.0)100:0.1,((Pasteurellaceae_Haemophilus_influenzae_10810_uid86647:1000.0,((Pasteurellaceae_Haemophilus_influenzae_KR494_uid219323:1000.0,Pasteurellaceae_Haemophilus_influenzae_PittGG_uid58593:1000.0)81:0.081,Pasteurellaceae_Haemophilus_influenzae_86_028NP_uid58093:1000.0)78:0.078)77:0.077,Pasteurellaceae_Haemophilus_influenzae_F3047_uid62097:1000.0)100:0.1)100:0.1)100:0.1,(((Coxiellaceae_Coxiella_burnetii_Dugway_5J108_111_uid58629:1000.0,(Coxiellaceae_Coxiella_burnetii_CbuK_Q154_uid58895:1000.0,((Coxiellaceae_Coxiella_burnetii_RSA_493_uid57631:1000.0,Coxiellaceae_Coxiella_burnetii_CbuG_Q212_uid58893:1000.0)59:0.059,Coxiellaceae_Coxiella_burnetii_RSA_331_uid58637:1000.0)59:0.059)100:0.1)100:0.1,(((Helicobacteraceae_Helicobacter_pylori_HPAG1_uid58517:1000.0,Helicobacteraceae_Helicobacter_pylori_26695_uid57787:1000.0)55:0.055,Helicobacteraceae_Helicobacter_pylori_India7_uid161149:1000.0)66:0.066,(Helicobacteraceae_Helicobacter_pylori_Gambia94_24_uid159493:1000.0,Helicobacteraceae_Helicobacter_pylori_2017_uid161151:1000.0)99:0.099)100:0.1)100:0.1,((Pasteurellaceae_Mannheimia_haemolytica_D153_uid212303:1000.0,((Pasteurellaceae_Mannheimia_haemolytica_D174_uid212305:1000.0,Pasteurellaceae_Mannheimia_haemolytica_USDA_ARS_USMARC_183_uid195458:1000.0)29:0.029,Pasteurellaceae_Mannheimia_haemolytica_USMARC_2286_uid213228:1000.0)27:0.027)47:0.047,Pasteurellaceae_Mannheimia_haemolytica_M42548_uid198769:1000.0)100:0.1)100:0.1)100:0.1,(Legionellaceae_Legionella_pneumophila_Lorraine_uid170535:1000.0,(Legionellaceae_Legionella_pneumophila_LPE509_uid193710:1000.0,((Legionellaceae_Legionella_pneumophila_Thunder_Bay_uid206517:1000.0,Legionellaceae_Legionella_pneumophila_Philadelphia_1_uid57609:1000.0)49:0.049,Legionellaceae_Legionella_pneumophila_Paris_uid58211:1000.0)35:0.035)44:0.044)100:0.1)100:0.1,((((Chlamydiaceae_Chlamydophila_psittaci_01DC11_uid159527:1000.0,Chlamydiaceae_Chlamydophila_psittaci_08DC60_uid159525:1000.0)29:0.029,(Chlamydiaceae_Chlamydophila_psittaci_6BC_uid63621:1000.0,Chlamydiaceae_Chlamydophila_psittaci_C19_98_uid159523:1000.0)33:0.033)40:0.04,Chlamydiaceae_Chlamydophila_psittaci_Mat116_uid189026:1000.0)100:0.1,(((((Chlamydiaceae_Chlamydophila_pneumoniae_TW_183_uid57997:1000.0,Chlamydiaceae_Chlamydophila_pneumoniae_J138_uid57829:1000.0)42:0.042,Chlamydiaceae_Chlamydophila_pneumoniae_AR39_uid57809:1000.0)32:0.032,Chlamydiaceae_Chlamydophila_pneumoniae_CWL029_uid57811:1000.0)58:0.058,Chlamydiaceae_Chlamydophila_pneumoniae_LPCoLN_uid159529:1000.0)100:0.1,(((Chlamydiaceae_Chlamydia_trachomatis_B_Jali20_OT_uid59351:1000.0,Chlamydiaceae_Chlamydia_trachomatis_434_Bu_uid61633:1000.0)30:0.03,(Chlamydiaceae_Chlamydia_trachomatis_IU824_uid193712:1000.0,Chlamydiaceae_Chlamydia_trachomatis_Sweden2_uid161995:1000.0)32:0.032)54:0.054,Chlamydiaceae_Chlamydia_trachomatis_A2497_uid159993:1000.0)100:0.1)100:0.1)100:0.1)100:0.1,(((Streptococcaceae_Streptococcus_pyogenes_HSC5_uid212978:1000.0,(Streptococcaceae_Streptococcus_pyogenes_A20_uid178106:1000.0,(Streptococcaceae_Streptococcus_pyogenes_Alab49_uid162171:1000.0,Streptococcaceae_Streptococcus_pyogenes_NZ131_uid59035:1000.0)43:0.043)51:0.051)66:0.066,Streptococcaceae_Streptococcus_pyogenes_MGAS9429_uid58569:1000.0)100:0.1,((((Streptococcaceae_Streptococcus_dysgalactiae_GGS_124_uid59103:1000.0,Streptococcaceae_Streptococcus_dysgalactiae_167_uid222822:1000.0)60:0.06,Streptococcaceae_Streptococcus_dysgalactiae_ATCC_12394_uid161979:1000.0)73:0.073,Streptococcaceae_Streptococcus_dysgalactiae_RE378_uid176684:1000.0)47:0.047,Streptococcaceae_Streptococcus_dysgalactiae_AC_2713_uid178644:1000.0)100:0.1)100:0.1)100:0.1,((((Streptococcaceae_Streptococcus_pneumoniae_TCH8431_19A_uid49735:1000.0,(Streptococcaceae_Streptococcus_pneumoniae_A026_uid226114:1000.0,Streptococcaceae_Streptococcus_pneumoniae_Taiwan19F_14_uid59119:1000.0)78:0.078)98:0.098,Streptococcaceae_Streptococcus_pneumoniae_TIGR4_uid57857:1000.0)75:0.075,Streptococcaceae_Streptococcus_pneumoniae_ATCC_700669_uid59287:1000.0)100:0.1,(((Streptococcaceae_Streptococcus_suis_05ZYH33_uid58663:1000.0,Streptococcaceae_Streptococcus_suis_98HAH33_uid58665:1000.0)41:0.041,(Streptococcaceae_Streptococcus_suis_A7_uid162111:1000.0,Streptococcaceae_Streptococcus_suis_D12_uid162127:1000.0)37:0.037)43:0.043,Streptococcaceae_Streptococcus_suis_BM407_uid59321:1000.0)100:0.1)100:0.1)100:0.1,((Alteromonadaceae_Alteromonas_macleodii_Black_Sea_11_uid176365:1000.0,Alteromonadaceae_Alteromonas_macleodii_Balearic_Sea_AD45_uid176366:1000.0)100:0.1,(Alteromonadaceae_Alteromonas_macleodii_Ionian_Sea_U4_uid210780:1000.0,(Alteromonadaceae_Alteromonas_macleodii_English_Channel_615_uid210781:1000.0,Alteromonadaceae_Alteromonas_macleodii_Deep_ecotype_uid58251:1000.0)92:0.092)100:0.1)100:0.1)100:0.1,(((((Bacillaceae_Bacillus_amyloliquefaciens_plantarum_AS43_3_uid183682:1000.0,(Bacillaceae_Bacillus_amyloliquefaciens_IT_45_uid181617:1000.0,Bacillaceae_Bacillus_amyloliquefaciens_CC178_uid226115:1000.0)40:0.04)46:0.046,Bacillaceae_Bacillus_amyloliquefaciens_FZB42_uid58271:1000.0)100:0.1,Bacillaceae_Bacillus_amyloliquefaciens_DSM_7_uid53535:1000.0)100:0.1,(((Bacillaceae_Bacillus_subtilis_BSn5_uid62463:1000.0,(Bacillaceae_Bacillus_subtilis_PY79_uid229877:1000.0,Bacillaceae_Bacillus_subtilis_BSP1_uid184010:1000.0)59:0.059)97:0.097,Bacillaceae_Bacillus_subtilis_natto_BEST195_uid183001:1000.0)85:0.085,Bacillaceae_Bacillus_subtilis_168_uid57675:1000.0)100:0.1)100:0.1,Bacteroidaceae_Bacteroides_fragilis_NCTC_9343_uid57639:1000.0)100:0.1)100:0.1,((Sphingomonadaceae_Zymomonas_mobilis_CP4_NRRL_B_14023_uid229874:1000.0,(Sphingomonadaceae_Zymomonas_mobilis_NCIMB_11163_uid41019:1000.0,(Sphingomonadaceae_Zymomonas_mobilis_ATCC_29191_uid170612:1000.0,Sphingomonadaceae_Zymomonas_mobilis_ATCC_10988_uid55403:1000.0)79:0.079)59:0.059)100:0.1,Sphingomonadaceae_Zymomonas_mobilis_pomaceae_ATCC_29192_uid68445:1000.0)100:0.1)100:0.1)100:0.1,((((Shewanellaceae_Shewanella_baltica_BA175_uid52601:1000.0,Shewanellaceae_Shewanella_baltica_OS185_uid58743:1000.0)58:0.058,Shewanellaceae_Shewanella_baltica_OS195_uid58261:1000.0)51:0.051,Shewanellaceae_Shewanella_baltica_OS155_uid58259:1000.0)84:0.084,Shewanellaceae_Shewanella_baltica_OS117_uid162025:1000.0)100:0.1)100:0.1,(((Vibrionaceae_Vibrio_cholerae_IEC224_uid89389:1000.0,(Vibrionaceae_Vibrio_cholerae_O395_uid58425:1000.0,(Vibrionaceae_Vibrio_cholerae_O1_biovar_El_Tor_N16961_uid57623:1000.0,Vibrionaceae_Vibrio_cholerae_LMA3984_4_uid159541:1000.0)52:0.052)49:0.049)81:0.081,Vibrionaceae_Vibrio_cholerae_MJ_1236_uid59387:1000.0)100:0.1,(Enterobacteriaceae_Yersinia_pestis_biovar_Medievalis_Harbin_35_uid158537:1000.0,(((Enterobacteriaceae_Yersinia_pestis_D106004_uid158071:1000.0,Enterobacteriaceae_Yersinia_pestis_CO92_uid57621:1000.0)41:0.041,Enterobacteriaceae_Yersinia_pestis_A1122_uid158119:1000.0)37:0.037,Enterobacteriaceae_Yersinia_pestis_Angola_uid58485:1000.0)59:0.059)100:0.1)64:0.064)100:0.1,(((((((((((((((Burkholderiaceae_Ralstonia_solanacearum_FQY_4_f_uid194089:1000.0,Burkholderiaceae_Ralstonia_solanacearum_GMI1000_uid57593:1000.0)84:0.084,Burkholderiaceae_Ralstonia_solanacearum_CMR15_uid227773:1000.0)100:0.1,Burkholderiaceae_Ralstonia_solanacearum_CFBP2957_uid50545:1000.0)100:0.1,Burkholderiaceae_Ralstonia_solanacearum_PSI07_uid50539:1000.0)100:0.1,(((Bradyrhizobiaceae_Rhodopseudomonas_palustris_CGA009_uid62901:1000.0,Bradyrhizobiaceae_Rhodopseudomonas_palustris_TIE_1_uid58995:1000.0)87:0.087,(Bradyrhizobiaceae_Rhodopseudomonas_palustris_DX_1_uid43327:1000.0,Bradyrhizobiaceae_Rhodopseudomonas_palustris_HaA2_uid58439:1000.0)100:0.1)100:0.1,Bradyrhizobiaceae_Rhodopseudomonas_palustris_BisA53_uid58445:1000.0)100:0.1)100:0.1,((((Burkholderiaceae_Burkholderia_pseudomallei_BPC006_uid174460:1000.0,Burkholderiaceae_Burkholderia_pseudomallei_668_uid58389:1000.0)56:0.056,Burkholderiaceae_Burkholderia_pseudomallei_1106a_uid58515:1000.0)100:0.1,Burkholderiaceae_Burkholderia_pseudomallei_NCTC_13179_uid226109:1000.0)100:0.1,Burkholderiaceae_Burkholderia_pseudomallei_MSHR346_uid55259:1000.0)100:0.1)100:0.1,((((Mycobacteriaceae_Mycobacterium_canettii_CIPT_140060008_uid184829:1000.0,(Mycobacteriaceae_Mycobacterium_canettii_CIPT_140070017_uid184830:1000.0,Mycobacteriaceae_Mycobacterium_tuberculosis_Erdman__ATCC_35801_uid193763:1000.0)26:0.026)14:0.014,(((Mycobacteriaceae_Mycobacterium_tuberculosis_EAI5_NITR206_uid202218:1000.0,Mycobacteriaceae_Mycobacterium_bovis_BCG_Pasteur_1173P2_uid58781:1000.0)14:0.014,((Mycobacteriaceae_Mycobacterium_canettii_CIPT_140010059_uid70731:1000.0,Mycobacteriaceae_Mycobacterium_tuberculosis_CCDC5079_uid161943:1000.0)20:0.02,Mycobacteriaceae_Mycobacterium_tuberculosis_CTRI_2_uid161997:1000.0)11:0.011)10:0.010,((Mycobacteriaceae_Mycobacterium_bovis_BCG_Korea_1168P_uid189029:1000.0,Mycobacteriaceae_Mycobacterium_bovis_AF2122_97_uid57695:1000.0)14:0.014,(Mycobacteriaceae_Mycobacterium_bovis_BCG_Mexico_uid86889:1000.0,Mycobacteriaceae_Mycobacterium_bovis_BCG_Tokyo_172_uid59281:1000.0)17:0.017)10:0.010)10:0.010)11:0.011,(Mycobacteriaceae_Mycobacterium_canettii_CIPT_140070008_uid184832:1000.0,Mycobacteriaceae_Mycobacterium_canettii_CIPT_140070010_uid184828:1000.0)17:0.017)14:0.014,Mycobacteriaceae_Mycobacterium_tuberculosis_Beijing_NITR203_uid197218:1000.0)100:0.1)100:0.1,(((((((Pseudomonadaceae_Pseudomonas_aeruginosa_LESB58_uid59275:1000.0,(Pseudomonadaceae_Pseudomonas_aeruginosa_NCGM2_S1_uid162173:1000.0,((Pseudomonadaceae_Pseudomonas_aeruginosa_B136_33_uid196598:1000.0,Pseudomonadaceae_Pseudomonas_aeruginosa_c7447m_uid219358:1000.0)97:0.097,Pseudomonadaceae_Pseudomonas_aeruginosa_DK2_uid168996:1000.0)100:0.1)65:0.065)100:0.1,(Pseudomonadaceae_Pseudomonas_stutzeri_ATCC_17588__LMG_11199_uid68749:1000.0,Pseudomonadaceae_Pseudomonas_stutzeri_A1501_uid58641:1000.0)100:0.1)100:0.1,Pseudomonadaceae_Pseudomonas_stutzeri_DSM_10701_uid170940:1000.0)100:0.1,(Pseudomonadaceae_Pseudomonas_stutzeri_CCUG_29243_uid168379:1000.0,Pseudomonadaceae_Pseudomonas_stutzeri_RCH2_uid184342:1000.0)81:0.081)79:0.079,((((Pseudomonadaceae_Pseudomonas_putida_BIRD_1_uid162055:1000.0,(Pseudomonadaceae_Pseudomonas_fluorescens_Pf_5_uid57937:1000.0,Pseudomonadaceae_Pseudomonas_putida_NBRC_14164_uid208670:1000.0)75:0.075)67:0.067,Pseudomonadaceae_Pseudomonas_putida_KT2440_uid57843:1000.0)47:0.047,Pseudomonadaceae_Pseudomonas_putida_H8234_uid208673:1000.0)81:0.081,Pseudomonadaceae_Pseudomonas_putida_W619_uid58651:1000.0)81:0.081)100:0.1,((Pseudomonadaceae_Pseudomonas_fluorescens_SBW25_uid158693:1000.0,Pseudomonadaceae_Pseudomonas_fluorescens_A506_uid165185:1000.0)92:0.092,Pseudomonadaceae_Pseudomonas_fluorescens_F113_uid87037:1000.0)74:0.074)74:0.074,Pseudomonadaceae_Pseudomonas_fluorescens_Pf0_1_uid57591:1000.0)100:0.1)100:0.1,(((Bifidobacteriaceae_Bifidobacterium_animalis_ATCC_25527_uid162513:1000.0,((Bifidobacteriaceae_Bifidobacterium_animalis_AD011_uid58911:1000.0,Bifidobacteriaceae_Bifidobacterium_animalis_ATCC_27673_uid222803:1000.0)50:0.05,Bifidobacteriaceae_Bifidobacterium_animalis_DSM_10140_uid59357:1000.0)55:0.055)100:0.1,Bifidobacteriaceae_Bifidobacterium_animalis_CNCM_I_2494_uid158869:1000.0)100:0.1,((Bifidobacteriaceae_Bifidobacterium_longum_BBMN68_uid60163:1000.0,((Bifidobacteriaceae_Bifidobacterium_longum_infantis_157F_uid62693:1000.0,Bifidobacteriaceae_Bifidobacterium_longum_DJO10A_uid58833:1000.0)46:0.046,Bifidobacteriaceae_Bifidobacterium_longum_F8_uid197184:1000.0)46:0.046)91:0.091,Bifidobacteriaceae_Bifidobacterium_longum_KACC_91563_uid158861:1000.0)100:0.1)100:0.1)100:0.1,((Propionibacteriaceae_Propionibacterium_acnes_6609_uid162137:1000.0,((Propionibacteriaceae_Propionibacterium_acnes_HL096PA1_uid198524:1000.0,Propionibacteriaceae_Propionibacterium_acnes_SK137_uid48071:1000.0)46:0.046,Propionibacteriaceae_Propionibacterium_acnes_ATCC_11828_uid162177:1000.0)66:0.066)100:0.1,Propionibacteriaceae_Propionibacterium_acnes_KPA171202_uid58101:1000.0)100:0.1)71:0.071,((((Brucellaceae_Brucella_melitensis_M5_90_uid158855:1000.0,Brucellaceae_Brucella_melitensis_M28_uid158857:1000.0)56:0.056,Brucellaceae_Brucella_melitensis_NI_uid158853:1000.0)100:0.1,Brucellaceae_Brucella_melitensis_ATCC_23457_uid59241:1000.0)100:0.1,Brucellaceae_Brucella_melitensis_bv_1_16M_uid57735:1000.0)100:0.1)71:0.071,((((Enterobacteriaceae_Enterobacter_cloacae_ENHKU01_uid172463:1000.0,Enterobacteriaceae_Enterobacter_cloacae_NCTC_9394_uid197202:1000.0)54:0.054,Enterobacteriaceae_Enterobacter_cloacae_dissolvens_SDM_uid168997:1000.0)94:0.094,(((Enterobacteriaceae_Klebsiella_pneumoniae_KCTC_2242_uid162147:1000.0,(Enterobacteriaceae_Klebsiella_pneumoniae_CG43_uid223021:1000.0,Enterobacteriaceae_Klebsiella_pneumoniae_1084_uid174151:1000.0)98:0.098)83:0.083,Enterobacteriaceae_Klebsiella_pneumoniae_JM45_uid215235:1000.0)100:0.1,Enterobacteriaceae_Klebsiella_pneumoniae_HS11286_uid84387:1000.0)100:0.1)62:0.062,(Enterobacteriaceae_Enterobacter_cloacae_EcWSU1_uid80739:1000.0,Enterobacteriaceae_Enterobacter_cloacae_ATCC_13047_uid48363:1000.0)49:0.049)71:0.071)82:0.082,((((Corynebacteriaceae_Corynebacterium_diphtheriae_CDCE_8392_uid84295:1000.0,(Corynebacteriaceae_Corynebacterium_diphtheriae_PW8_uid84303:1000.0,Corynebacteriaceae_Corynebacterium_diphtheriae_INCA_402_uid83605:1000.0)46:0.046)63:0.063,Corynebacteriaceae_Corynebacterium_diphtheriae_VA01_uid84305:1000.0)64:0.064,Corynebacteriaceae_Corynebacterium_diphtheriae_NCTC_13129_uid57691:1000.0)100:0.1,(((Corynebacteriaceae_Corynebacterium_glutamicum_SCgG1_uid207285:1000.0,(Corynebacteriaceae_Corynebacterium_glutamicum_MB001_uid214793:1000.0,Corynebacteriaceae_Corynebacterium_glutamicum_R_uid58897:1000.0)90:0.09)53:0.053,Corynebacteriaceae_Corynebacterium_glutamicum_SCgG2_uid207286:1000.0)83:0.083,Corynebacteriaceae_Corynebacterium_glutamicum_ATCC_13032_uid193708:1000.0)100:0.1)100:0.1)48:0.048,((((Xanthomonadaceae_Xylella_fastidiosa_GB514_uid162023:1000.0,Xanthomonadaceae_Xylella_fastidiosa_M12_uid58763:1000.0)60:0.06,Xanthomonadaceae_Xylella_fastidiosa_Temecula1_uid57869:1000.0)44:0.044,Xanthomonadaceae_Xylella_fastidiosa_M23_uid58809:1000.0)49:0.049,Xanthomonadaceae_Xylella_fastidiosa_9a5c_uid57849:1000.0)100:0.1)43:0.043,(((Corynebacteriaceae_Corynebacterium_pseudotuberculosis_CIP_52_97_uid159667:1000.0,Corynebacteriaceae_Corynebacterium_pseudotuberculosis_316_uid89381:1000.0)41:0.041,(Corynebacteriaceae_Corynebacterium_pseudotuberculosis_1002_uid159677:1000.0,Corynebacteriaceae_Corynebacterium_pseudotuberculosis_267_uid162175:1000.0)23:0.023)31:0.031,Corynebacteriaceae_Corynebacterium_pseudotuberculosis_C231_uid159675:1000.0)100:0.1)48:0.048,((((Spirochaetaceae_Treponema_pallidum_Fribourg_Blanc_uid201428:1000.0,Spirochaetaceae_Treponema_pallidum_pertenue_SamoaD_uid87069:1000.0)45:0.045,Spirochaetaceae_Treponema_pallidum_Mexico_A_uid176920:1000.0)37:0.037,Spirochaetaceae_Treponema_pallidum_SS14_uid58977:1000.0)92:0.092,Spirochaetaceae_Treponema_pallidum_Chicago_uid159543:1000.0)100:0.1)48:0.048)48:0.048,(((((Neisseriaceae_Neisseria_meningitidis_alpha710_uid161971:1000.0,Neisseriaceae_Neisseria_meningitidis_G2136_uid162085:1000.0)37:0.037,Neisseriaceae_Neisseria_meningitidis_H44_76_uid162083:1000.0)36:0.036,Neisseriaceae_Neisseria_meningitidis_053442_uid58587:1000.0)48:0.048,Neisseriaceae_Neisseria_meningitidis_FAM18_uid57825:1000.0)100:0.1,(((((Enterobacteriaceae_Escherichia_coli_BL21_Gold_DE3_pLysS_AG_uid59245:1000.0,Enterobacteriaceae_Escherichia_coli_ATCC_8739_uid58783:1000.0)40:0.04,Enterobacteriaceae_Escherichia_coli_K_12_substr_MG1655_uid57779:1000.0)61:0.061,Enterobacteriaceae_Escherichia_coli_B_REL606_uid58803:1000.0)100:0.1,Enterobacteriaceae_Escherichia_coli_ETEC_H10407_uid161993:1000.0)100:0.1,(((Enterobacteriaceae_Salmonella_enterica_serovar_Newport_SL254_uid58831:1000.0,(Enterobacteriaceae_Salmonella_enterica_serovar_Agona_24249_uid230614:1000.0,Enterobacteriaceae_Salmonella_enterica_serovar_Javiana_CFSAN001992_uid190101:1000.0)75:0.075)62:0.062,Enterobacteriaceae_Salmonella_enterica_serovar_Heidelberg_SL476_uid58973:1000.0)100:0.1,Enterobacteriaceae_Salmonella_enterica_arizonae_serovar_62_z4_z23_uid58191:1000.0)84:0.084)48:0.048)46:0.046)100:0.1,Acetobacteraceae_Acetobacter_pasteurianus_386B_uid214433:1000.0)100:0.1,(Acetobacteraceae_Acetobacter_pasteurianus_IFO_3283_07_uid158381:1000.0,(Acetobacteraceae_Acetobacter_pasteurianus_IFO_3283_12_uid158379:1000.0,Acetobacteraceae_Acetobacter_pasteurianus_IFO_3283_03_uid158373:1000.0)29:0.029)35:0.035)100:0.1,Acetobacteraceae_Acetobacter_pasteurianus_IFO_3283_01_uid59279:1000.0);}

Figure S5. The tree with the Newick format based on the codon usage of the different bacterial genomes.

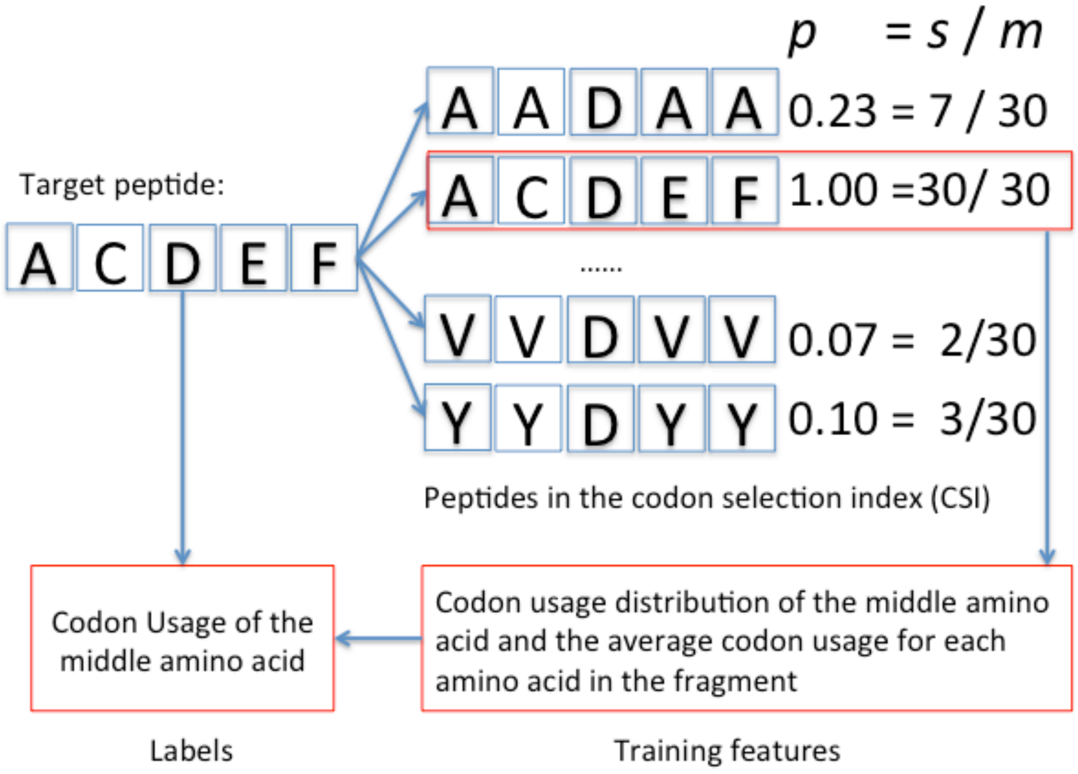

Figure S6. An illustration for preparing the training labels and features for the target peptide (ACDEF). The matched percent (p, $p = s/m$) for each fragment was assessed, which is the percent of matching between a calculated matched score (s) and expected maximal score (m) of the target fragment.

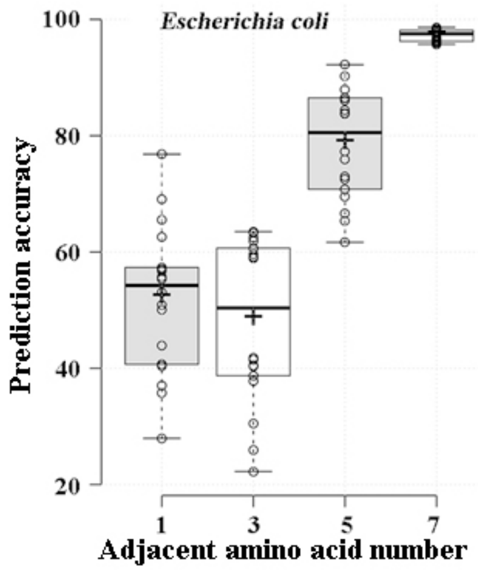

Figure S7. The prediction accuracy with different adjacent residue number. The x-axis represents the different adjacent amino acid number and the y-axis is the prediction accuracy of the 18 classifiers. Each point is one of the 18 classifiers. The line in the box is the upper whisker, 3rd quartile, Median, 1st quartile and the Lower whisker, respectively. The cross in the figure is the mean of the prediction accuracy of the 18 classifiers. All of the results were calculated based on a ten-fold cross validation.

```
egfp-genscript ATGGTTAGCAAAGGCGAGGAACTGTTCACCGGTGTGGTTCCGATCCTGGTGGAGCTGGAC
egfp-codon     ATGGTTTCCAAAGGCGAGGAACTCTTTACCGGTGTGGTCCCCATTCTGGTCGAGCTGGAC
consensus      ******  *************** ** *********** ** ** ***** *********
               1.......10........20........30........40........50........60

egfp-genscript GGCGATGTTAACGGTCACAAGTTTAGCGTGAGCGGCGAGGGCGAAGGTGACGCGACCTAC
egfp-codon     GGCGACGTCAATGGTCATAAATTCTCAGTTTCCGGCGAAGGTGAAGGCGACGCAACTTAC
consensus      ***** ** ** ***** **.**    **   ******.** ***** *****.** ***
               ........70........80........90.......100.......110.......120

egfp-genscript GGCAAGCTGACCCTGAAATTCATTTGCACCACCGGTAAACTGCCGGTGCCGTGGCCGACC
egfp-codon     GGAAAATTGACACTAAAGTTTATTTGTACCACAGGGAAATTGCCAGTTCCATGGCCGACG
consensus      ** **. **** **.**.** ***** ***** ** *** ****.** **.******** 
               .......130.......140.......150.......160.......170.......180

egfp-genscript CTGGTTACCACCCTGACCTACGGTGTTCAGTGCTTTAGCCGTTATCCGGACCACATGAAG
egfp-codon     CTGGTGACAACCCTAACCTATGGTGTTCAATGCTTTTCCCGCTATCCGGATCACATGAAG
consensus      ***** ** *****.***** ********.******  *** ******** *********
               .......190.......200.......210.......220.......230.......240

egfp-genscript CAACACGATTTCTTTAAAAGCGCGATGCCGGAGGGCTACGTGCAGGAACGTACCATCTTC
egfp-codon     CAACACGACTTCTTCAAATCAGCTATGCCAGAAGGGTACGTTCAGGAAAGAACGATCTTC
consensus      ******** ***** ***   ** *****.**.** ***** ****** * ** ******
               .......250.......260.......270.......280.......290.......300

egfp-genscript TTTAAGGACGATGGTAACTATAAAACCCGTGCGGAAGTGAAGTTCGAAGGCGACACCCTG
egfp-codon     TTCAAGGACGATGGTAATTACAAAACCCGTGCTGAAGTCAAGTTCGAGGGCGATACGCTG
consensus      ** ************** ** *********** ***** ********.***** ** ***
               .......310.......320.......330.......340.......350.......360

egfp-genscript GTTAACCGTATCGAGCTGAAGGGTATTGACTTTAAAGAAGATGGCAACATTCTGGGTCAC
egfp-codon     GTTAACCGGATTGAACTTAAGGGAATCGATTTTAAAGAGGACGGTAATATCCTCGGGCAT
consensus      ******** ** **.** ***** ** ** ********.** ** ** ** ** ** ** 
               .......370.......380.......390.......400.......410.......420

egfp-genscript AAGCTGGAGTACAACTATAACAGCCACAACGTGTACATCATGGCGGATAAGCAGAAAAAC
egfp-codon     AAACTGGAGTATAATTACAACTCTCATAATGTCTATATCATGGCTGACAAGCAAAAAAAT
consensus      **.******** ** ** ***   ** ** ** ** ******** ** *****.***** 
               .......430.......440.......450.......460.......470.......480

egfp-genscript GGCATCAAGGTTAACTTCAAGATCCGTCACAACATTGAAGACGGTAGCGTGCAACTGGCG
egfp-codon     GGCATCAAAGTTAATTTTAAAATTCGCCATAATATTGAAGATGGCAGCGTACAGCTGGCC
consensus      ********.***** ** **.** ** ** ** ******** ** *****.**.***** 
               .......490.......500.......510.......520.......530.......540

egfp-genscript GATCACTACCAGCAAAACACCCCGATCGGTGACGGTCCGGTTCTGCTGCCGGATAACCAC
egfp-codon     GATCATTACCAACAAAATACGCCCATTGGCGATGGGCCCGTGCTATTACCCGATAACCAT
consensus      ***** *****.***** ** ** ** ** ** ** ** ** **. *.** ******** 
               .......550.......560.......570.......580.......590.......600

egfp-genscript TATCTGAGCACCCAAAGCGCGCTGAGCAAGGACCCGAACGAGAAACGTGATCACATGGTG
egfp-codon     TATTTATCAACCCAATCCGCCTTGAGTAAAGATCCCAACGAAAAAAGAGACCATATGGTG
consensus      *** *.   ******  ***  **** **.** ** *****.*** * ** ** ******
               .......610.......620.......630.......640.......650.......660

egfp-genscript CTGCTGGAATTCGTTACCGCGGCGGGCATTACCCTGGGTATGGATGAACTGTATAAA
egfp-codon     CTGCTGGAATTCGTGACAGCAGCTGGAATTACCCTCGGCATGGATGAACTATATAAA
consensus      ************** ** **.** ** ******** ** ***********.******
               .......670.......680.......690.......700.......710.......
```

Figure S8. Sequence alignment of the *egfp* genes (*egfp-codon* and *egfp-genscript*).

```
mApple-genscript ATGGTTAGCAAGGGCGAGGAAAACAACATGGCGATCATTAAGGAGTTCATGCGTTTTAAA
mApple-codon     ATGGTTTCCAAAGGAGAAGAAAATAATATGGCAATCATTAAAGAATTTATGCGATTCAAA
consensus        ******  ***.** **.***** ** *****.********.**.** ***** ** ***
                 1.......10........20........30........40........50........60

mApple-genscript GTGCACATGGAAGGCAGCGTTAACGGTCACGAGTTTGAAATCGAGGGTGAAGGCGAGGGT
mApple-codon     GTTCATATGGAAGGCAGTGTTAACGGCCACGAGTTTGAAATCGAAGGCGAAGGGGAAGGA
consensus        ** ** *********** ******** *****************.** ***** **.**
                 ........70........80........90.......100.......110.......120

mApple-genscript CGTCCGTACGAGGCGTTCCAGACCGCGAAGCTGAAAGTGACCAAGGGTGGCCCGCTGCCG
mApple-codon     AGACCCTACGAAGCATTCCAGACAGCCAAGCTGAAAGTTACAAAAGGCGGCCCGCTGCCT
consensus         * ** *****.**.******** ** *********** ** **.** ***********
                 .......130.......140.......150.......160.......170.......180

mApple-genscript TTTGCGTGGGACATTCTGAGCCCGCAATTCATGTACGGCAGCAAGGTTTATATCAAACAC
mApple-codon     TTCGCCTGGGATATATTATCACCTCAATTTATGTACGGTTCAAAGGTTTATATCAAACAT
consensus        ** ** ***** **  *.   ** ***** ********    *****************
                 .......190.......200.......210.......220.......230.......240

mApple-genscript CCGGCGGACATTCCGGATTATTTCAAACTGAGCTTTCCGGAGGGTTTCCGTTGGGAACGT
mApple-codon     CCTGCGGACATACCGGACTATTTCAAATTAAGTTTCCCAGAAGGTTTTCGCTGGGAGCGC
consensus        ** ******** ***** ********* *.** ** **.**.***** ** *****.**
                 .......250.......260.......270.......280.......290.......300

mApple-genscript GTGATGAACTTTGAAGACGGTGGCATCATTCACGTTAACCAGGACAGCAGCCTGCAAGAT
mApple-codon     GTGATGAATTTTGAAGACGGCGGTATCATTCACGTTAATCAGGACTCCTCGCTTCAAGAT
consensus        ******** *********** ** ************** ******  *   ** ******
                 .......310.......320.......330.......340.......350.......360

mApple-genscript GGTGTGTTTATCTACAAGGTTAAACTGCGTGGCACCAACTTCCCGAGCGATGGTCCGGTG
mApple-codon     GGGGTGTTTATCTACAAGGTCAAGCTGCGCGGCACTAACTTCCCCTCTGACGGTCCAGTC
consensus        ** ***************** **.***** ***** ********    ** *****.**
                 .......370.......380.......390.......400.......410.......420

mApple-genscript ATGCAGAAGAAAACCATGGGCTGGGAGGCGAGCGAGGAACGTATGTATCCGGAGGACGGT
mApple-codon     ATGCAAAAAAAAACCATGGGCTGGGAAGCGTCTGAAGAGCGGATGTACCCGGAAGACGGT
consensus        *****.**.*****************.***   **.**.** ***** *****.******
                 .......430.......440.......450.......460.......470.......480

mApple-genscript GCGCTGAAAAGCGAAATTAAGAAACGTCTGAAGCTGAAAGATGGTGGCCACTACGCGGCG
mApple-codon     GCACTGAAAAGTGAAATCAAAAAAAGATTAAAGTTAAAAGATGGCGGTCATTATGCGGCC
consensus        **.******** ***** **.*** *  *.*** *.******** ** ** ** *****
                 .......490.......500.......510.......520.......530.......540

mApple-genscript GAAGTGAAGACCACCTATAAAGCGAAGAAACCGGTTCAGCTGCCGGGCGCGTACATCGTG
mApple-codon     GAGGTGAAAACGACCTACAAAGCAAAGAAGCCAGTGCAACTACCTGGTGCTTACATTGTT
consensus        **.*****.** ***** *****.*****.**.** **.**.** ** ** ***** **
                 .......550.......560.......570.......580.......590.......600

mApple-genscript GACATTAAGCTGGATATCGTTAGCCACAACGAGGACTACACCATTGTTGAACAATATGAG
mApple-codon     GACATCAAGCTGGACATCGTGAGTCACAATGAAGATTACACCATTGTTGAACAGTATGAA
consensus        ***** ******** ***** ** ***** **.** *****************.*****.
                 .......610.......620.......630.......640.......650.......660

mApple-genscript CGTGCGGAAGGTCGTCACAGCACCGGTGGCATGGATGAACTGTATAAA
mApple-codon     CGAGCTGAAGGACGTCACTCCACCGGCGGGATGGATGAACTATATAAA
consensus        ** ** ***** ******  ****** ** ***********.******
                 .......670.......680.......690.......700........
```

Figure S9. Sequence alignment of the *mApple* genes (*mApple-codon* and *mApple-genscript*).

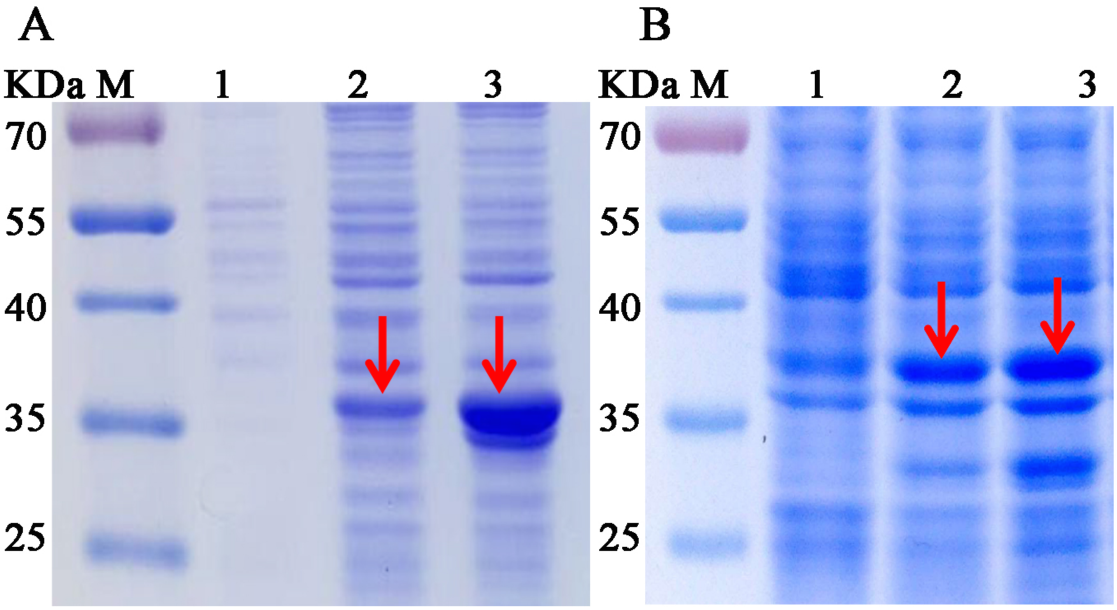

Figure S10. SDS-PAGE analysis of the expressed eGFP(A) and mApple(B). Lanes M and 1 represent the marker and lysate supernatant from *E.coli* BL21 containing the vector pET30a(+). In the panel A, the lanes 2 and 3 represent the lysate supernatant from *E.coli* BL21 containing the vector eGFP-genscript, eGFP-codon, which was inoculated at the auto-induction medium at 30°C for about 20 hours. In the panel B, the lanes 2 and 3 represent the lysate supernatant from *E.coli* BL21 containing the vector mApple-genscript, mApple –codon, which was also inoculated at the auto-induction medium at 30°C for about 20 hours.

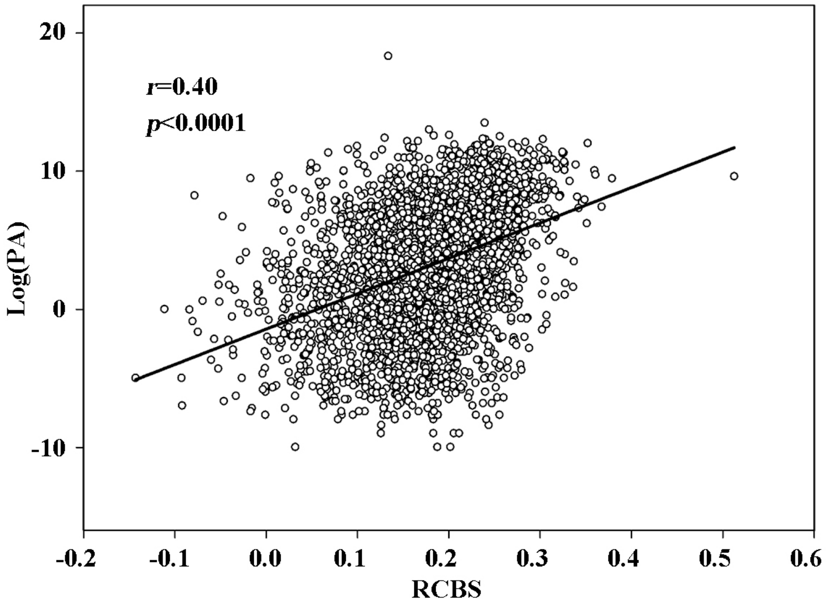

Figure S11. Dot plots of log(Protein abundance, PA) vs. relative codon bias (RCBS) and the corresponding Spearman rank correlations between Log(pA) and RCBS. The Protein abundance data of E.coli were downloaded from the PaxDb database.

Table S1. The selected Bacterium names and its NCBI uids.

Number	Bacterium name and its uid in NCBI
1	Acetobacter_pasteurianus_386B_uid214433
2	Acetobacter_pasteurianus_IFO_3283_01_uid59279
3	Acetobacter_pasteurianus_IFO_3283_03_uid158373
4	Acetobacter_pasteurianus_IFO_3283_07_uid158381
5	Acetobacter_pasteurianus_IFO_3283_12_uid158379
6	Acinetobacter_baumannii_1656_2_uid158677
7	Acinetobacter_baumannii_AB0057_uid59083
8	Acinetobacter_baumannii_ACICU_uid58765
9	Acinetobacter_baumannii_ATCC_17978_uid58731
10	Acinetobacter_baumannii_AYE_uid61637
11	Alteromonas_macleodii_Balearic_Sea_AD45_uid176366
12	Alteromonas_macleodii_Black_Sea_11_uid176365
13	Alteromonas_macleodii_Deep_ecotype_uid58251
14	Alteromonas_macleodii_English_Channel_615_uid210781
15	Alteromonas_macleodii_Ionian_Sea_U4_uid210780
16	Bacillus_amyloliquefaciens_CC178_uid226115
17	Bacillus_amyloliquefaciens_DSM_7_uid53535
18	Bacillus_amyloliquefaciens_FZB42_uid58271
19	Bacillus_amyloliquefaciens_IT_45_uid181617
20	Bacillus_amyloliquefaciens_plantarum_AS43_3_uid183682
21	Bacillus_anthracis_A0248_uid59385
22	Bacillus_anthracis_Ames_Ancestor_uid58083
23	Bacillus_anthracis_CDC_684_uid59303
24	Bacillus_anthracis_H9401_uid162021
25	Bacillus_anthracis_Sterne_uid58091
26	Bacillus_cereus_03BB102_uid59299
27	Bacillus_cereus_AH187_uid58753
28	Bacillus_cereus_ATCC_10987_uid57673
29	Bacillus_cereus_B4264_uid58757
30	Bacillus_cereus_biovar_anthracis_CI_uid50615
31	Bacillus_subtilis_168_uid57675
32	Bacillus_subtilis_BSn5_uid62463
33	Bacillus_subtilis_BSP1_uid184010
34	Bacillus_subtilis_natto_BEST195_uid183001
35	Bacillus_subtilis_PY79_uid229877
36	Bacillus_thuringiensis_Al_Hakam_uid58795
37	Bacillus_thuringiensis_BMB171_uid49135
38	Bacillus_thuringiensis_Bt407_uid177931
39	Bacillus_thuringiensis_HD_771_uid173374

**40**	Bacillus_thuringiensis_MC28_uid176369
**41**	Bacteroides_fragilis_NCTC_9343_uid57639
**42**	Bifidobacterium_animalis_ATCC_25527_uid162513
**43**	Bifidobacterium_animalis_lactis_AD011_uid58911
**44**	Bifidobacterium_animalis_lactis_ATCC_27673_uid222803
**45**	Bifidobacterium_animalis_lactis_CNCM_I_2494_uid158869
**46**	Bifidobacterium_animalis_lactis_DSM_10140_uid59357
**47**	Bifidobacterium_longum_BBMN68_uid60163
**48**	Bifidobacterium_longum_DJO10A_uid58833
**49**	Bifidobacterium_longum_F8_uid197184
**50**	Bifidobacterium_longum_infantis_157F_uid62693
**51**	Bifidobacterium_longum_KACC_91563_uid158861
**52**	Borrelia_burgdorferi_B31_uid57581
**53**	Borrelia_burgdorferi_CA382_uid214794
**54**	Borrelia_burgdorferi_JD1_uid161197
**55**	Borrelia_burgdorferi_N40_uid161241
**56**	Borrelia_burgdorferi_ZS7_uid59429
**57**	Brucella_melitensis_ATCC_23457_uid59241
**58**	Brucella_melitensis_bv_1_16M_uid57735
**59**	Brucella_melitensis_M28_uid158857
**60**	Brucella_melitensis_M5_90_uid158855
**61**	Brucella_melitensis_NI_uid158853
**62**	Burkholderia_pseudomallei_1106a_uid58515
**63**	Burkholderia_pseudomallei_668_uid58389
**64**	Burkholderia_pseudomallei_BPC006_uid174460
**65**	Burkholderia_pseudomallei_MSHR346_uid55259
**66**	Burkholderia_pseudomallei_NCTC_13179_uid226109
**67**	Campylobacter_jejuni_00_2544_uid219326
**68**	Campylobacter_jejuni_4031_uid222817
**69**	Campylobacter_jejuni_81_176_uid58503
**70**	Campylobacter_jejuni_81116_uid58771
**71**	Campylobacter_jejuni_doylei_269_97_uid58671
**72**	Candidatus_Kinetoplastibacterium_blastocrithidii_ex_Strigomonas_culicis_uid183629
**73**	Candidatus_Kinetoplastibacterium_crithidii_ex_Angomonas_deanei_ATCC_30255_uid183630
**74**	Candidatus_Kinetoplastibacterium_crithidii_TCC036E_uid189749
**75**	Candidatus_Kinetoplastibacterium_desouzaii_TCC079E_uid189750
**76**	Candidatus_Kinetoplastibacterium_oncopeltii_TCC290E_uid189840
**77**	Chlamydia_trachomatis_434_Bu_uid61633
**78**	Chlamydia_trachomatis_A2497_uid159993

**79**	Chlamydia_trachomatis_B_Jali20_OT_uid59351
**80**	Chlamydia_trachomatis_IU824_uid193712
**81**	Chlamydia_trachomatis_Sweden2_uid161995
**82**	Chlamydophila_pneumoniae_AR39_uid57809
**83**	Chlamydophila_pneumoniae_CWL029_uid57811
**84**	Chlamydophila_pneumoniae_J138_uid57829
**85**	Chlamydophila_pneumoniae_LPCoLN_uid159529
**86**	Chlamydophila_pneumoniae_TW_183_uid57997
**87**	Chlamydophila_psittaci_01DC11_uid159527
**88**	Chlamydophila_psittaci_08DC60_uid159525
**89**	Chlamydophila_psittaci_6BC_uid63621
**90**	Chlamydophila_psittaci_C19_98_uid159523
**91**	Chlamydophila_psittaci_Mat116_uid189026
**92**	Clostridium_botulinum_A_ATCC_19397_uid58927
**93**	Clostridium_botulinum_A_ATCC_3502_uid61579
**94**	Clostridium_botulinum_F_230613_uid159513
**95**	Clostridium_botulinum_F_Langeland_uid58929
**96**	Clostridium_botulinum_H04402_065_uid162091
**97**	Corynebacterium_diphtheriae_CDCE_8392_uid84295
**98**	Corynebacterium_diphtheriae_INCA_402_uid83605
**99**	Corynebacterium_diphtheriae_NCTC_13129_uid57691
**100**	Corynebacterium_diphtheriae_PW8_uid84303
**101**	Corynebacterium_diphtheriae_VA01_uid84305
**102**	Corynebacterium_glutamicum_ATCC_13032_uid193708
**103**	Corynebacterium_glutamicum_MB001_uid214793
**104**	Corynebacterium_glutamicum_R_uid58897
**105**	Corynebacterium_glutamicum_SCgG1_uid207285
**106**	Corynebacterium_glutamicum_SCgG2_uid207286
**107**	Corynebacterium_pseudotuberculosis_1002_uid159677
**108**	Corynebacterium_pseudotuberculosis_267_uid162175
**109**	Corynebacterium_pseudotuberculosis_316_uid89381
**110**	Corynebacterium_pseudotuberculosis_C231_uid159675
**111**	Corynebacterium_pseudotuberculosis_CIP_52_97_uid159667
**112**	Coxiella_burnetii_CbuG_Q212_uid58893
**113**	Coxiella_burnetii_CbuK_Q154_uid58895
**114**	Coxiella_burnetii_Dugway_5J108_111_uid58629
**115**	Coxiella_burnetii_RSA_331_uid58637
**116**	Coxiella_burnetii_RSA_493_uid57631
**117**	Enterobacter_cloacae_ATCC_13047_uid48363
**118**	Enterobacter_cloacae_dissolvens_SDM_uid168997
**119**	Enterobacter_cloacae_EcWSU1_uid80739

**120**	Enterobacter_cloacae_ENHKU01_uid172463
**121**	Enterobacter_cloacae_NCTC_9394_uid197202
**122**	Enterococcus_faecalis_62_uid159663
**123**	Enterococcus_faecalis_D32_uid171261
**124**	Enterococcus_faecalis_OG1RF_uid54927
**125**	Enterococcus_faecalis_Symbioflor_1_uid183342
**126**	Enterococcus_faecalis_V583_uid57669
**127**	Escherichia_coli_ATCC_8739_uid58783
**128**	Escherichia_coli_B_REL606_uid58803
**129**	Escherichia_coli_BL21_Gold_DE3_pLysS_AG_uid59245
**130**	Escherichia_coli_ETEC_H10407_uid161993
**131**	Escherichia_coli_K_12_substr_MG1655_uid57779
**132**	Francisella_tularensis_FSC198_uid58693
**133**	Francisella_tularensis_holarctica_F92_uid181998
**134**	Francisella_tularensis_mediasiatica_FSC147_uid58939
**135**	Francisella_tularensis_NE061598_uid161973
**136**	Francisella_tularensis_SCHU_S4_uid57589
**137**	Haemophilus_influenzae_10810_uid86647
**138**	Haemophilus_influenzae_86_028NP_uid58093
**139**	Haemophilus_influenzae_F3047_uid62097
**140**	Haemophilus_influenzae_KR494_uid219323
**141**	Haemophilus_influenzae_PittGG_uid58593
**142**	Helicobacter_pylori_2017_uid161151
**143**	Helicobacter_pylori_26695_uid57787
**144**	Helicobacter_pylori_Gambia94_24_uid159493
**145**	Helicobacter_pylori_HPAG1_uid58517
**146**	Helicobacter_pylori_India7_uid161149
**147**	Klebsiella_pneumoniae_1084_uid174151
**148**	Klebsiella_pneumoniae_CG43_uid223021
**149**	Klebsiella_pneumoniae_HS11286_uid84387
**150**	Klebsiella_pneumoniae_JM45_uid215235
**151**	Klebsiella_pneumoniae_KCTC_2242_uid162147
**152**	Lactobacillus_casei_ATCC_334_uid57985
**153**	Lactobacillus_casei_BL23_uid59237
**154**	Lactobacillus_casei_LOCK919_uid210959
**155**	Lactobacillus_casei_W56_uid178736
**156**	Lactobacillus_casei_Zhang_uid50673
**157**	Lactobacillus_plantarum_JDM1_uid59361
**158**	Lactobacillus_plantarum_P8_uid203333
**159**	Lactobacillus_plantarum_ST_III_uid53537
**160**	Lactobacillus_plantarum_WCFS1_uid62911

**161**	Lactobacillus_plantarum_ZJ316_uid188689
**162**	Lactobacillus_reuteri_DSM_20016_uid58471
**163**	Lactobacillus_reuteri_I5007_uid208677
**164**	Lactobacillus_reuteri_JCM_1112_uid58875
**165**	Lactobacillus_reuteri_SD2112_uid55357
**166**	Lactobacillus_reuteri_TD1_uid213089
**167**	Lactobacillus_rhamnosus_ATCC_8530_uid162169
**168**	Lactobacillus_rhamnosus_GG_uid59313
**169**	Lactobacillus_rhamnosus_Lc_705_uid59315
**170**	Lactobacillus_rhamnosus_LOCK900_uid210957
**171**	Lactobacillus_rhamnosus_LOCK908_uid210958
**172**	Lactococcus_lactis_CV56_uid160253
**173**	Lactococcus_lactis_Il1403_uid57671
**174**	Lactococcus_lactis_IO_1_uid192185
**175**	Lactococcus_lactis_KF147_uid42831
**176**	Lactococcus_lactis_KLDS_4_0325_uid225028
**177**	Legionella_pneumophila_Lorraine_uid170535
**178**	Legionella_pneumophila_LPE509_uid193710
**179**	Legionella_pneumophila_Paris_uid58211
**180**	Legionella_pneumophila_Philadelphia_1_uid57609
**181**	Legionella_pneumophila_Thunder_Bay_uid206517
**182**	Listeria_monocytogenes_07PF0776_uid162185
**183**	Listeria_monocytogenes_10403S_uid54461
**184**	Listeria_monocytogenes_ATCC_19117_uid175109
**185**	Listeria_monocytogenes_Clip80459_uid59317
**186**	Listeria_monocytogenes_Finland_1998_uid54443
**187**	Mannheimia_haemolytica_D153_uid212303
**188**	Mannheimia_haemolytica_D174_uid212305
**189**	Mannheimia_haemolytica_M42548_uid198769
**190**	Mannheimia_haemolytica_USDA_ARS_USMARC_183_uid195458
**191**	Mannheimia_haemolytica_USMARC_2286_uid213228
**192**	Mycobacterium_bovis_AF2122_97_uid57695
**193**	Mycobacterium_bovis_BCG_Korea_1168P_uid189029
**194**	Mycobacterium_bovis_BCG_Mexico_uid86889
**195**	Mycobacterium_bovis_BCG_Pasteur_1173P2_uid58781
**196**	Mycobacterium_bovis_BCG_Tokyo_172_uid59281
**197**	Mycobacterium_canettii_CIPT_140010059_uid70731
**198**	Mycobacterium_canettii_CIPT_140060008_uid184829
**199**	Mycobacterium_canettii_CIPT_140070008_uid184832
**200**	Mycobacterium_canettii_CIPT_140070010_uid184828
**201**	Mycobacterium_canettii_CIPT_140070017_uid184830

**202**	Mycobacterium_tuberculosis_Beijing_NITR203_uid197218
**203**	Mycobacterium_tuberculosis_CCDC5079_uid161943
**204**	Mycobacterium_tuberculosis_CTRI_2_uid161997
**205**	Mycobacterium_tuberculosis_EAI5_NITR206_uid202218
**206**	Mycobacterium_tuberculosis_Erdman__ATCC_35801_uid193763
**207**	Mycoplasma_gallisepticum_CA06_2006_052_5_2P_uid172630
**208**	Mycoplasma_gallisepticum_NC06_2006_080_5_2P_uid172629
**209**	Mycoplasma_gallisepticum_R_low_uid57993
**210**	Mycoplasma_gallisepticum_VA94_7994_1_7P_uid172624
**211**	Mycoplasma_gallisepticum_WI01_2001_043_13_2P_uid172628
**212**	Mycoplasma_hyopneumoniae_168_uid162053
**213**	Mycoplasma_hyopneumoniae_232_uid58205
**214**	Mycoplasma_hyopneumoniae_7422_uid212968
**215**	Mycoplasma_hyopneumoniae_7448_uid58039
**216**	Mycoplasma_hyopneumoniae_J_uid58059
**217**	Mycoplasma_hyorhinis_DBS_1050_uid228933
**218**	Mycoplasma_hyorhinis_GDL_1_uid87003
**219**	Mycoplasma_hyorhinis_HUB_1_uid51695
**220**	Mycoplasma_hyorhinis_MCLD_uid162087
**221**	Mycoplasma_hyorhinis_SK76_uid181997
**222**	Neisseria_meningitidis_053442_uid58587
**223**	Neisseria_meningitidis_alpha710_uid161971
**224**	Neisseria_meningitidis_FAM18_uid57825
**225**	Neisseria_meningitidis_G2136_uid162085
**226**	Neisseria_meningitidis_H44_76_uid162083
**227**	Prochlorococcus_marinus_AS9601_uid58307
**228**	Prochlorococcus_marinus_CCMP1375_uid57995
**229**	Prochlorococcus_marinus_MIT_9211_uid58309
**230**	Prochlorococcus_marinus_NATL2A_uid58359
**231**	Prochlorococcus_marinus_pastoris_CCMP1986_uid57761
**232**	Propionibacterium_acnes_6609_uid162137
**233**	Propionibacterium_acnes_ATCC_11828_uid162177
**234**	Propionibacterium_acnes_HL096PA1_uid198524
**235**	Propionibacterium_acnes_KPA171202_uid58101
**236**	Propionibacterium_acnes_SK137_uid48071
**237**	Pseudomonas_aeruginosa_B136_33_uid196598
**238**	Pseudomonas_aeruginosa_c7447m_uid219358
**239**	Pseudomonas_aeruginosa_DK2_uid168996
**240**	Pseudomonas_aeruginosa_LESB58_uid59275
**241**	Pseudomonas_aeruginosa_NCGM2_S1_uid162173
**242**	Pseudomonas_fluorescens_A506_uid165185

**243**	Pseudomonas_fluorescens_F113_uid87037
**244**	Pseudomonas_fluorescens_Pf_5_uid57937
**245**	Pseudomonas_fluorescens_Pf0_1_uid57591
**246**	Pseudomonas_fluorescens_SBW25_uid158693
**247**	Pseudomonas_putida_BIRD_1_uid162055
**248**	Pseudomonas_putida_H8234_uid208673
**249**	Pseudomonas_putida_KT2440_uid57843
**250**	Pseudomonas_putida_NBRC_14164_uid208670
**251**	Pseudomonas_putida_W619_uid58651
**252**	Pseudomonas_stutzeri_A1501_uid58641
**253**	Pseudomonas_stutzeri_ATCC_17588__LMG_11199_uid68749
**254**	Pseudomonas_stutzeri_CCUG_29243_uid168379
**255**	Pseudomonas_stutzeri_DSM_10701_uid170940
**256**	Pseudomonas_stutzeri_RCH2_uid184342
**257**	Ralstonia_solanacearum_CFBP2957_uid50545
**258**	Ralstonia_solanacearum_CMR15_uid227773
**259**	Ralstonia_solanacearum_FQY_4_f_uid194089
**260**	Ralstonia_solanacearum_GMI1000_uid57593
**261**	Ralstonia_solanacearum_PSI07_uid50539
**262**	Rhodopseudomonas_palustris_BisA53_uid58445
**263**	Rhodopseudomonas_palustris_CGA009_uid62901
**264**	Rhodopseudomonas_palustris_DX_1_uid43327
**265**	Rhodopseudomonas_palustris_HaA2_uid58439
**266**	Rhodopseudomonas_palustris_TIE_1_uid58995
**267**	Rickettsia_prowazekii_Breinl_uid196851
**268**	Rickettsia_prowazekii_BuV67_CWPP_uid158063
**269**	Rickettsia_prowazekii_Chernikova_uid158053
**270**	Rickettsia_prowazekii_Dachau_uid158057
**271**	Rickettsia_prowazekii_GvV257_uid158051
**272**	Rickettsia_rickettsii_Arizona_uid86655
**273**	Rickettsia_rickettsii_Brazil_uid88069
**274**	Rickettsia_rickettsii_Colombia_uid86653
**275**	Rickettsia_rickettsii_Hauke_uid86659
**276**	Rickettsia_rickettsii_Sheila_Smith_uid58027
**277**	Salmonella_enterica_arizonae_serovar_62_z4_z23_uid58191
**278**	Salmonella_enterica_serovar_Agona_24249_uid230614
**279**	Salmonella_enterica_serovar_Heidelberg_SL476_uid58973
**280**	Salmonella_enterica_serovar_Javiana_CFSAN001992_uid190101
**281**	Salmonella_enterica_serovar_Newport_SL254_uid58831
**282**	Shewanella_baltica_BA175_uid52601
**283**	Shewanella_baltica_OS117_uid162025

**284**	Shewanella_baltica_OS155_uid58259
**285**	Shewanella_baltica_OS185_uid58743
**286**	Shewanella_baltica_OS195_uid58261
**287**	Staphylococcus_aureus_04_02981_uid161969
**288**	Staphylococcus_aureus_08BA02176_uid175257
**289**	Staphylococcus_aureus_11819_97_uid159981
**290**	Staphylococcus_aureus_55_2053_uid55909
**291**	Staphylococcus_aureus_6850_uid217772
**292**	Streptococcus_agalactiae_09mas018883_uid208674
**293**	Streptococcus_agalactiae_2603V_R_uid57943
**294**	Streptococcus_agalactiae_A909_uid57935
**295**	Streptococcus_agalactiae_GD201008_001_uid175780
**296**	Streptococcus_agalactiae_ILRI005_uid208676
**297**	Streptococcus_dysgalactiae_equisimilis_167_uid222822
**298**	Streptococcus_dysgalactiae_equisimilis_AC_2713_uid178644
**299**	Streptococcus_dysgalactiae_equisimilis_ATCC_12394_uid161979
**300**	Streptococcus_dysgalactiae_equisimilis_GGS_124_uid59103
**301**	Streptococcus_dysgalactiae_equisimilis_RE378_uid176684
**302**	Streptococcus_pneumoniae_A026_uid226114
**303**	Streptococcus_pneumoniae_ATCC_700669_uid59287
**304**	Streptococcus_pneumoniae_Taiwan19F_14_uid59119
**305**	Streptococcus_pneumoniae_TCH8431_19A_uid49735
**306**	Streptococcus_pneumoniae_TIGR4_uid57857
**307**	Streptococcus_pyogenes_A20_uid178106
**308**	Streptococcus_pyogenes_Alab49_uid162171
**309**	Streptococcus_pyogenes_HSC5_uid212978
**310**	Streptococcus_pyogenes_MGAS9429_uid58569
**311**	Streptococcus_pyogenes_NZ131_uid59035
**312**	Streptococcus_suis_05ZYH33_uid58663
**313**	Streptococcus_suis_98HAH33_uid58665
**314**	Streptococcus_suis_A7_uid162111
**315**	Streptococcus_suis_BM407_uid59321
**316**	Streptococcus_suis_D12_uid162127
**317**	Treponema_pallidum_Chicago_uid159543
**318**	Treponema_pallidum_Fribourg_Blanc_uid201428
**319**	Treponema_pallidum_Mexico_A_uid176920
**320**	Treponema_pallidum_pertenue_SamoaD_uid87069
**321**	Treponema_pallidum_SS14_uid58977
**322**	Vibrio_cholerae_IEC224_uid89389
**323**	Vibrio_cholerae_LMA3984_4_uid159541
**324**	Vibrio_cholerae_MJ_1236_uid59387

**325**	Vibrio_cholerae_O1_biovar_El_Tor_N16961_uid57623
**326**	Vibrio_cholerae_O395_uid58425
**327**	Wolbachia_endosymbiont_of_Culex_quinquefasciatus_Pel_uid61645
**328**	Wolbachia_endosymbiont_of_Drosophila_melanogaster_uid57851
**329**	Wolbachia_endosymbiont_of_Drosophila_simulans_wHa_uid198768
**330**	Wolbachia_endosymbiont_of_Drosophila_simulans_wNo_uid198767
**331**	Wolbachia_endosymbiont_TRS_of_Brugia_malayi_uid58107
**332**	Xylella_fastidiosa_9a5c_uid57849
**333**	Xylella_fastidiosa_GB514_uid162023
**334**	Xylella_fastidiosa_M12_uid58763
**335**	Xylella_fastidiosa_M23_uid58809
**336**	Xylella_fastidiosa_Temecula1_uid57869
**337**	Yersinia_pestis_A1122_uid158119
**338**	Yersinia_pestis_Angola_uid58485
**339**	Yersinia_pestis_biovar_Medievalis_Harbin_35_uid158537
**340**	Yersinia_pestis_CO92_uid57621
**341**	Yersinia_pestis_D106004_uid158071
**342**	Zymomonas_mobilis_ATCC_10988_uid55403
**343**	Zymomonas_mobilis_ATCC_29191_uid170612
**344**	Zymomonas_mobilis_CP4_NRRL_B_14023_uid229874
**345**	Zymomonas_mobilis_NCIMB_11163_uid41019
**346**	Zymomonas_mobilis_pomaceae_ATCC_29192_uid68445
**347**	Bacillus_subtilis_6051_HGW_uid193706
**348**	Bacillus_subtilis_BAB_1_uid195461
**349**	Bacillus_subtilis_BSP1_uid184010
**350**	Bacillus_subtilis_BSn5_uid62463
**351**	Bacillus_subtilis_PY79_uid229877
**352**	Bacillus_subtilis_QB928_uid173926
**353**	Bacillus_subtilis_RO_NN_1_uid158879
**354**	Bacillus_subtilis_XF_1_uid189187
**355**	Bacillus_subtilis_natto_BEST195_uid183001
**356**	Bacillus_subtilis_spizizenii_TU_B_10_uid73967
**357**	Bacillus_subtilis_spizizenii_W23_uid51879
**358**	Escherichia_coli_042_uid161985
**359**	Escherichia_coli_536_uid58531
**360**	Escherichia_coli_55989_uid59383
**361**	Escherichia_coli_ABU_83972_uid161975
**362**	Escherichia_coli_APEC_O1_uid58623
**363**	Escherichia_coli_APEC_O78_uid187277
**364**	Escherichia_coli_ATCC_8739_uid58783
**365**	Escherichia_coli_BL21_DE3__uid161947

**366**	Escherichia_coli_BL21_DE3__uid161949
**367**	Escherichia_coli_BW2952_uid59391
**368**	Escherichia_coli_B_REL606_uid58803
**369**	Escherichia_coli_CFT073_uid57915
**370**	Escherichia_coli_DH1_uid161951
**371**	Escherichia_coli_DH1_uid162051
**372**	Escherichia_coli_E24377A_uid58395
**373**	Escherichia_coli_ED1a_uid59379
**374**	Escherichia_coli_ETEC_H10407_uid161993
**375**	Escherichia_coli_HS_uid58393
**376**	Escherichia_coli_IAI1_uid59377
**377**	Escherichia_coli_IAI39_uid59381
**378**	Escherichia_coli_IHE3034_uid162007
**379**	Escherichia_coli_JJ1886_uid226103
**380**	Escherichia_coli_KO11FL_uid162099
**381**	Escherichia_coli_KO11FL_uid52593
**382**	Escherichia_coli_K_12_substr__DH10B_uid58979
**383**	Escherichia_coli_K_12_substr__MDS42_uid193705
**384**	Escherichia_coli_K_12_substr__W3110_uid161931
**385**	Escherichia_coli_LF82_uid161965
**386**	Escherichia_coli_LY180_uid219461
**387**	Escherichia_coli_NA114_uid162139
**388**	Escherichia_coli_O103_H2_12009_uid41013
**389**	Escherichia_coli_O104_H4_2009EL_2050_uid175905
**390**	Escherichia_coli_O104_H4_2009EL_2071_uid176128
**391**	Escherichia_coli_O104_H4_2011C_3493_uid176127
**392**	Escherichia_coli_O111_H__11128_uid41023
**393**	Escherichia_coli_O127_H6_E2348_69_uid59343
**394**	Escherichia_coli_O157_H7_EC4115_uid59091
**395**	Escherichia_coli_O157_H7_EDL933_uid57831
**396**	Escherichia_coli_O157_H7_TW14359_uid59235
**397**	Escherichia_coli_O26_H11_11368_uid41021
**398**	Escherichia_coli_O55_H7_CB9615_uid46655
**399**	Escherichia_coli_O55_H7_RM12579_uid162153
**400**	Escherichia_coli_O7_K1_CE10_uid162115
**401**	Escherichia_coli_O83_H1_NRG_857C_uid161987
**402**	Escherichia_coli_P12b_uid162061
**403**	Escherichia_coli_PMV_1_uid219679
**404**	Escherichia_coli_S88_uid62979
**405**	Escherichia_coli_SE11_uid59425
**406**	Escherichia_coli_SE15_uid161939

**407**	Escherichia_coli_SMS_3_5_uid58919
**408**	Escherichia_coli_UM146_uid162043
**409**	Escherichia_coli_UMN026_uid62981
**410**	Escherichia_coli_UMNK88_uid161991
**411**	Escherichia_coli_UTI89_uid58541
**412**	Escherichia_coli_W_uid162011
**413**	Escherichia_coli_W_uid162101
**414**	Escherichia_coli_Xuzhou21_uid163995
**415**	Escherichia_coli_BL21_Gold_DE3_pLysS_AG__uid59245
**416**	Escherichia_coli_clone_D_i14__uid162049
**417**	Escherichia_coli_clone_D_i2__uid162047

All of the genomes were downloaded from NCBI (ftp://ftp.ncbi.nlm.nih.gov/genomes/Bacteria/).

Table S2. The prediction performance with different parameters

Amino Acid	*w*	*p*	Sum of the codon number	Overall Accuracy of the amino acid (%)	AUC(Area Under roc Curve)	ACC_Codon_1 (%)	ACC_Codon_2 (%)	ACC_Codon_3 (%)	ACC_Codon_4 (%)	ACC_Codon_5 (%)	ACC_Codon_6 (%)
**A**	5	0.7	125440	41.3±0.2	0.611±0.003	GCT 33.8±0.7	GCC 48.8±0.5	GCA 33.1±0.8	GCG 43.8±0.6		
**C**	5	0.7	15200	60.6±1.4	0.605±0.015	TGT 60.1±2.1	TGC 60.9±1.3				
**D**	5	0.7	67903	62.2±0.4	0.609±0.003	GAT 65.8±0.6	GAC 56.0±0.7				
**E**	5	0.7	75884	64.5±0.6	0.650±0.006	GAA 63.7±0.8	GAG 66.3±1.1				
**F**	5	0.7	51191	63.7±0.7	0.633±0.006	TTT 66.1±1.1	TTC 60.7±0.5				
**G**	5	0.7	97247	41.1±0.6	0.624±0.006	GGT 33.0±1.3	GGC 44.9±1.0	GGA 36.9±1.4	GGG 52.1±1.0		
**H**	5	0.7	29691	62.7±1.0	0.623±0.009	CAT 65.8±1.3	CAC 58.7±1.2				
**I**	5	0.7	79143	44.9±0.5	0.585±0.009	ATT 46.5±0.5	ATC 42.6±0.8	ATA 47.9±2.5			
**K**	5	0.7	56860	61.7±0.3	0.624±0.006	AAA 61.1±0.5	AAG 63.7±1.4				
**L**	5	0.7	140843	31.3±0.3	0.549±0.004	TTA 14.4±1.0	TTG 35.2±1.4	CTT 26.8±1.1	CTC 48.2±1.1	CTA 47.1±1.6	CTG 31.0±0.7
**N**	5	0.7	51223	59.9±0.9	0.600±0.009	AAT 60.9±2.0	AAC 59.0±1.9				

**P**	5	0.7	58470	38.5±0.9	0.602±0.004	CCT 37.1±0.9	CCC 39.0±1.6	CCA 36.1±1.6	CCG 39.7±1.3		
**Q**	5	0.7	58340	64.4±0.6	0.655±0.004	CAA 69.0±0.8	CAG 61.9±1.1				
**R**	5	0.7	72386	27.7±0.6	0.590±0.013	CGT 25.0±1.0	CGC 27.1±1.0	CGA 25.9±2.1	CGG 37.6±2.4	AGA 34.6±2.3	AGG 36.3±4.7
**S**	5	0.7	75493	28.3±0.5	0.587±0.005	TCT 25.2±0.9	TCC 36.6±1.1	TCA 21.7±1.6	TCG 41.5±1.2	AGT 26.4±1.5	AGC 21.9±0.8
**T**	5	0.7	70883	42.1±0.5	0.621±0.004	ACT 39.7±1.3	ACC 42.5±0.8	ACA 26.9±0.9	ACG 50.0±0.8		
**V**	5	0.7	93466	40.0±0.3	0.602±0.003	GTT 36.1±1.0	GTC 41.4±1.0	GTA 40.1±1.0	GTG 41.8±0.7		
**Y**	5	0.7	37372	61.4±0.8	0.610±0.008	TAT 64.0±1.0	TAC 57.9±1.5				
**A**	5	0.75	125440	42.1±0.4	0.616±0.003	GCT 34.6±0.7	GCC 49.3±0.6	GCA 35.1±0.6	GCG 44.1±0.6		
**C**	5	0.75	15200	62.8±1.2	0.629±0.012	TGT 63.3±2.0	TGC 62.4±1.2				
**D**	5	0.75	67903	62.9±0.4	0.618±0.004	GAT 65.9±0.6	GAC 57.8±0.8				
**E**	5	0.75	75884	65.3±0.6	0.658±0.006	GAA 64.6±0.7	GAG 67.0±1.0				
**F**	5	0.75	51191	64.7±0.6	0.643±0.006	TTT 66.6±1.0	TTC 62.1±0.8				
**G**	5	0.75	97247	42.2±0.7	0.629±0.005	GGT 34.6±1.2	GGC 45.8±1.1	GGA 38.3±1.7	GGG 52.0±1.3		

**H**	5	0.75	29691	64.0±1.0	0.635±0.009	CAT 66.8±1.9	CAC 60.3±1.5				
**I**	5	0.75	79143	46.5±0.4	0.599±0.007	ATT 48.6±0.8	ATC 43.5±0.7	ATA 49.9±2.3			
**K**	5	0.75	56860	63.2±0.6	0.635±0.006	AAA 62.9±0.6	AAG 64.3±0.9				
**L**	5	0.75	140843	32.2±0.4	0.555±0.005	TTA 17.0±1.5	TTG 35.2±1.3	CTT 28.4±1.1	CTC 48.9±0.7	CTA 49.4±2.2	CTG 31.5±0.8
**N**	5	0.75	51223	60.8±1.0	0.610±0.010	AAT 63.2±2.5	AAC 58.8±1.6				
**P**	5	0.75	58470	40.0±0.9	0.610±0.005	CCT 39.4±1.0	CCC 40.5±1.3	CCA 37.8±1.1	CCG 40.8±1.5		
**Q**	5	0.75	58340	65.4±0.7	0.666±0.006	CAA 70.3±1.0	CAG 62.8±1.1				
**R**	5	0.75	72386	29.2±0.8	0.600±0.012	CGT 25.9±1.3	CGC 28.7±1.0	CGA 32.5±2.5	CGG 37.9±2.3	AGA 36.4±2.0	AGG 40.2±5.0
**S**	5	0.75	75493	30.1±0.6	0.591±0.006	TCT 27.5±1.7	TCC 38.2±1.6	TCA 23.9±2.2	TCG 42.2±1.3	AGT 28.1±1.3	AGC 24.0±1.0
**T**	5	0.75	70883	43.1±0.3	0.630±0.004	ACT 41.5±1.4	ACC 42.5±0.5	ACA 30.6±0.9	ACG 51.0±0.9		
**V**	5	0.75	93466	40.6±0.3	0.605±0.003	GTT 36.0±0.6	GTC 43.0±0.9	GTA 41.5±1.5	GTG 42.1±0.7		
**Y**	5	0.75	37372	62.5±0.8	0.621±0.008	TAT 64.9±1.2	TAC 59.3±1.2				
**A**	5	0.8	125440	43.2±0.3	0.623±0.004	GCT 37.3±0.8	GCC 49.1±0.7	GCA 36.7±0.7	GCG 45.1±0.6		

**C**	5	0.8	15200	65.8±1.1	0.660±0.011	TGT 67.4±1.6	TGC 64.5±1.3				
**D**	5	0.8	67903	64.2±0.4	0.635±0.004	GAT 66.3±0.5	GAC 60.7±0.5				
**E**	5	0.8	75884	66.3±0.4	0.668±0.005	GAA 65.5±0.6	GAG 68.2±1.0				
**F**	5	0.8	51191	66.2±0.7	0.659±0.006	TTT 67.9±1.1	TTC 63.8±0.6				
**G**	5	0.8	97247	43.4±0.5	0.637±0.005	GGT 36.0±1.3	GGC 46.9±1.1	GGA 40.2±1.3	GGG 52.7±1.3		
**H**	5	0.8	29691	66.4±1.0	0.659±0.009	CAT 68.9±1.6	CAC 62.9±1.2				
**I**	5	0.8	79143	48.3±0.7	0.616±0.009	ATT 49.7±0.9	ATC 45.8±0.8	ATA 53.5±2.0			
**K**	5	0.8	56860	65.1±0.6	0.652±0.007	AAA 64.9±0.8	AAG 65.4±1.4				
**L**	5	0.8	140843	33.4±0.2	0.567±0.004	TTA 20.4±1.3	TTG 36.9±1.3	CTT 31.6±0.9	CTC 49.5±1.2	CTA 51.4±1.9	CTG 31.6±0.7
**N**	5	0.8	51223	62.5±0.7	0.627±0.007	AAT 64.3±1.7	AAC 61.1±1.5				
**P**	5	0.8	58470	42.0±0.4	0.621±0.004	CCT 43.2±1.9	CCC 44.7±1.4	CCA 39.9±1.2	CCG 41.7±0.9		
**Q**	5	0.8	58340	66.3±0.8	0.674±0.007	CAA 71.0±0.8	CAG 63.9±1.1				
**R**	5	0.8	72386	30.7±0.9	0.619±0.010	CGT 26.6±1.2	CGC 29.3±1.0	CGA 36.1±2.4	CGG 42.2±1.7	AGA 41.6±2.3	AGG 47.7±5.2

**S**	5	0.8	75493	32.6±0.5	0.601±0.005	TCT 30.9±1.4	TCC 39.2±1.5	TCA 29.3±1.9	TCG 43.8±0.8	AGT 31.0±1.3	AGC 26.2±0.9
**T**	5	0.8	70883	44.8±0.3	0.639±0.003	ACT 45.0±1.4	ACC 44.1±0.6	ACA 34.9±1.2	ACG 50.6±0.9		
**V**	5	0.8	93466	42.2±0.4	0.615±0.003	GTT 37.6±0.5	GTC 44.5±0.9	GTA 44.9±1.5	GTG 42.9±0.8		
**Y**	5	0.8	37372	64.9±0.8	0.646±0.008	TAT 67.0±1.0	TAC 62.1±1.1				
**A**	5	0.85	125440	45.5±0.3	0.637±0.003	GCT 41.9±0.9	GCC 51.2±0.6	GCA 40.0±0.7	GCG 46.1±0.8		
**C**	5	0.85	15200	71.0±1.2	0.710±0.013	TGT 71.1±2.1	TGC 71.0±1.1				
**D**	5	0.85	67903	66.0±0.3	0.658±0.003	GAT 66.8±0.5	GAC 64.8±0.7				
**E**	5	0.85	75884	68.6±0.6	0.689±0.007	GAA 68.1±0.7	GAG 69.7±0.9				
**F**	5	0.85	51191	68.5±0.8	0.685±0.008	TTT 69.0±1.3	TTC 67.9±1.0				
**G**	5	0.85	97247	45.8±0.5	0.651±0.006	GGT 39.5±1.1	GGC 47.5±0.8	GGA 46.9±1.4	GGG 54.7±1.5		
**H**	5	0.85	29691	69.8±0.7	0.696±0.007	CAT 70.9±1.4	CAC 68.2±1.3				
**I**	5	0.85	79143	51.7±0.5	0.651±0.009	ATT 52.1±0.6	ATC 49.9±1.0	ATA 59.9±2.6			
**K**	5	0.85	56859	68.7±0.6	0.688±0.003	AAA 68.7±1.0	AAG 68.8±1.1				

**L**	5	0.85	140843	36.0±0.4	0.587±0.003	TTA 26.9±1.2	TTG 39.0±1.1	CTT 37.1±1.3	CTC 51.5±0.8	CTA 56.6±1.7	CTG 32.7±0.5
**N**	5	0.85	51223	65.7±0.6	0.658±0.006	AAT 66.3±1.6	AAC 65.3±1.3				
**P**	5	0.85	58470	45.5±0.5	0.640±0.003	CCT 47.8±1.4	CCC 50.9±1.4	CCA 45.2±1.0	CCG 43.6±0.8		
**Q**	5	0.85	58340	68.7±0.5	0.699±0.004	CAA 73.4±0.8	CAG 66.2±0.7				
**R**	5	0.85	72386	35.2±0.6	0.651±0.007	CGT 30.1±0.7	CGC 33.6±0.7	CGA 45.4±1.4	CGG 46.5±2.4	AGA 49.7±2.1	AGG 54.8±4.6
**S**	5	0.85	75493	37.1±0.5	0.622±0.007	TCT 35.7±1.5	TCC 44.4±1.2	TCA 34.9±2.2	TCG 48.0±1.7	AGT 35.2±1.6	AGC 29.8±0.9
**T**	5	0.85	70883	48.0±0.5	0.659±0.003	ACT 50.1±1.8	ACC 45.6±0.9	ACA 40.8±1.9	ACG 54.0±1.1		
**V**	5	0.85	93466	45.8±0.5	0.634±0.004	GTT 41.4±1.0	GTC 48.7±1.1	GTA 49.9±1.9	GTG 45.5±0.8		
**Y**	5	0.85	37372	67.8±0.7	0.677±0.007	TAT 68.4±1.0	TAC 66.9±1.0				
**A**	5	0.9	125440	49.9±0.2	0.663±0.003	GCT 48.3±1.2	GCC 55.0±0.7	GCA 46.0±1.0	GCG 49.2±0.9		
**C**	5	0.9	15200	79.5±0.8	0.796±0.008	TGT 80.5±2.1	TGC 78.7±1.6				
**D**	5	0.9	67903	71.2±0.3	0.710±0.003	GAT 71.8±0.6	GAC 70.2±0.7				
**E**	5	0.9	75884	72.1±0.6	0.724±0.008	GAA 71.6±0.5	GAG 73.2±1.3				

**F**	5	0.9	51191	74.2±0.4	0.742±0.004	TTT 74.3±0.9	TTC 74.0±0.9				
**G**	5	0.9	97247	51.2±0.4	0.685±0.004	GGT 45.2±1.2	GGC 51.5±1.5	GGA 54.8±1.1	GGG 61.2±0.8		
**H**	5	0.9	29691	76.8±0.9	0.768±0.009	CAT 76.6±1.0	CAC 77.0±1.2				
**I**	5	0.9	79143	58.3±0.5	0.703±0.009	ATT 58.2±0.7	ATC 57.1±0.6	ATA 67.1±2.3			
**K**	5	0.9	56859	72.7±0.6	0.736±0.006	AAA 71.9±0.7	AAG 75.2±0.9				
**L**	5	0.9	140843	40.9±0.2	0.624±0.002	TTA 36.4±1.4	TTG 44.6±1.3	CTT 44.5±1.3	CTC 55.8±1.3	CTA 63.0±2.3	CTG 35.7±0.5
**N**	5	0.9	51223	70.7±0.5	0.708±0.005	AAT 71.3±1.2	AAC 70.3±1.0				
**P**	5	0.9	58470	54.3±0.8	0.695±0.005	CCT 57.2±1.6	CCC 60.8±2.5	CCA 55.4±1.6	CCG 51.5±1.4		
**Q**	5	0.9	58340	73.7±0.7	0.746±0.007	CAA 77.4±0.8	CAG 71.7±0.9				
**R**	5	0.9	72386	43.5±0.6	0.700±0.006	CGT 38.9±1.4	CGC 41.1±0.5	CGA 57.0±1.9	CGG 53.5±1.7	AGA 58.5±3.1	AGG 64.9±2.7
**S**	5	0.9	75493	45.2±0.6	0.665±0.004	TCT 44.7±2.1	TCC 50.5±1.5	TCA 43.9±2.4	TCG 53.4±1.2	AGT 44.4±2.1	AGC 39.0±1.0
**T**	5	0.9	70883	54.0±0.6	0.695±0.005	ACT 56.2±0.8	ACC 51.5±1.0	ACA 51.2±1.6	ACG 58.2±1.1		
**V**	5	0.9	93466	51.5±0.7	0.667±0.005	GTT 47.0±1.0	GTC 55.4±1.1	GTA 56.9±1.4	GTG 50.1±0.9		

**Y**	5	0.9	37372	74.6±0.6	0.743±0.006	TAT 76.2±0.8	TAC 72.4±0.8				
**A**	5	0.95	125438	58.9±0.5	0.719±0.004	GCT 61.9±1.1	GCC 62.0±1.1	GCA 56.9±1.1	GCG 56.3±1.3		
**C**	5	0.95	15197	87.4±1.2	0.875±0.012	TGT 88.3±1.7	TGC 86.7±1.2				
**D**	5	0.95	67903	78.8±0.4	0.789±0.003	GAT 78.3±0.7	GAC 79.5±0.7				
**E**	5	0.95	75881	78.7±0.2	0.797±0.002	GAA 77.1±0.4	GAG 82.2±0.7				
**F**	5	0.95	51189	81.8±0.4	0.819±0.004	TTT 81.1±0.8	TTC 82.7±0.7				
**G**	5	0.95	97246	62.4±0.6	0.757±0.005	GGT 58.3±1.2	GGC 60.7±0.8	GGA 68.6±1.6	GGG 72.0±1.4		
**H**	5	0.95	29691	85.8±0.7	0.858±0.006	CAT 85.7±1.2	CAC 85.9±0.3				
**I**	5	0.95	79141	69.2±0.8	0.788±0.009	ATT 67.5±1.2	ATC 69.3±1.2	ATA 80.2±2.3			
**K**	5	0.95	56858	80.1±0.5	0.814±0.006	AAA 78.9±0.6	AAG 84.0±0.8				
**L**	5	0.95	140843	52.6±0.4	0.705±0.005	TTA 50.8±1.0	TTG 58.1±1.4	CTT 59.3±1.2	CTC 68.4±1.1	CTA 77.2±1.9	CTG 45.2±0.6
**N**	5	0.95	51221	78.7±0.6	0.788±0.006	AAT 79.9±0.9	AAC 77.7±0.4				
**P**	5	0.95	58468	66.9±0.5	0.776±0.003	CCT 68.5±1.5	CCC 73.1±1.4	CCA 66.3±1.3	CCG 65.2±1.1		

**Q**	5	0.95	58336	80.8±0.3	0.817±0.004	CAA 84.4±0.9	CAG 78.9±0.6				
**R**	5	0.95	72386	58.9±0.4	0.791±0.007	CGT 54.7±0.6	CGC 56.5±0.8	CGA 72.6±2.1	CGG 68.3±2.2	AGA 71.4±2.7	AGG 78.7±2.7
**S**	5	0.95	75492	57.9±0.6	0.736±0.005	TCT 58.1±1.6	TCC 61.2±1.0	TCA 58.1±1.9	TCG 64.6±2.2	AGT 57.6±1.6	AGC 52.5±1.2
**T**	5	0.95	70881	64.5±0.6	0.762±0.004	ACT 66.4±0.7	ACC 60.9±0.8	ACA 68.1±1.4	ACG 67.6±0.8		
**V**	5	0.95	93465	61.9±0.5	0.731±0.005	GTT 59.8±0.9	GTC 65.4±1.1	GTA 69.0±1.5	GTG 58.2±1.3		
**Y**	5	0.95	37372	83.4±0.4	0.834±0.004	TAT 83.7±0.8	TAC 83.0±1.0				
**A**	5	1	125437	65.5±0.4	0.768±0.004	GCT 71.3±1.3	GCC 68.3±2.0	GCA 65.6±1.1	GCG 60.9±1.3		
**C**	5	1	15195	92.2±0.8	0.923±0.007	TGT 93.1±1.1	TGC 91.5±1.0				
**D**	5	1	67903	84.1±0.4	0.848±0.004	GAT 82.2±0.7	GAC 87.4±0.9				
**E**	5	1	75879	83.9±0.4	0.851±0.004	GAA 82.0±0.5	GAG 88.3±0.7				
**F**	5	1	51188	86.5±0.5	0.867±0.006	TTT 85.6±0.8	TTC 87.8±1.4				
**G**	5	1	97244	70.9±0.5	0.821±0.005	GGT 67.5±1.0	GGC 67.9±0.9	GGA 80.5±1.6	GGG 80.0±1.0		
**H**	5	1	29691	90.3±0.7	0.906±0.007	CAT 88.7±0.8	CAC 92.5±1.0				

**I**	5	1	79139	77.5±0.5	0.862±0.006	ATT 74.2±0.8	ATC 79.4±0.5	ATA 89.6±1.3			
**K**	5	1	56857	86.3±0.5	0.881±0.006	AAA 84.7±0.6	AAG 91.4±1.2				
**L**	5	1	140840	61.5±0.3	0.764±0.006	TTA 61.8±0.7	TTG 67.4±1.0	CTT 70.2±1.3	CTC 75.9±0.9	CTA 84.6±1.7	CTG 53.5±0.6
**N**	5	1	51221	84.7±0.4	0.849±0.004	AAT 86.2±0.8	AAC 83.5±0.4				
**P**	5	1	58468	75.9±0.4	0.844±0.004	CCT 78.7±1.2	CCC 85.0±1.2	CCA 76.9±0.9	CCG 72.6±0.6		
**Q**	5	1	58335	86.5±0.6	0.874±0.006	CAA 90.6±1.0	CAG 84.3±0.6				
**R**	5	1	72385	72.3±0.9	0.875±0.006	CGT 69.5±1.2	CGC 69.4±1.1	CGA 84.1±1.6	CGG 80.6±1.2	AGA 84.6±1.6	AGG 88.0±2.2
**S**	5	1	75491	66.9±0.4	0.790±0.004	TCT 67.3±2.5	TCC 69.7±1.4	TCA 70.9±1.6	TCG 69.8±0.7	AGT 67.1±1.5	AGC 61.6±1.3
**T**	5	1	70881	72.9±0.4	0.822±0.003	ACT 76.4±1.5	ACC 69.2±0.5	ACA 79.2±1.5	ACG 73.8±0.7		
**V**	5	1	93464	69.6±0.5	0.789±0.002	GTT 67.7±1.2	GTC 74.2±0.9	GTA 78.7±1.7	GTG 64.6±1.3		
**Y**	5	1	37371	88.0±0.5	0.882±0.005	TAT 86.5±0.9	TAC 89.9±0.7				
**A**	7	0.7	124939	50.9±0.5	0.671±0.005	GCT 53.2±1.2	GCC 53.3±0.9	GCA 48.1±1.0	GCG 49.8±0.7		
**C**	7	0.7	15139	75.9±1.4	0.758±0.014	TGT 75.5±1.3	TGC 76.2±1.9				

**D**	7	0.7	67518	70.3±0.6	0.701±0.006	GAT 70.7±0.7	GAC 69.5±1.0				
**E**	7	0.7	75292	72.3±0.9	0.729±0.010	GAA 71.4±1.0	GAG 74.4±1.5				
**F**	7	0.7	50929	73.2±0.5	0.731±0.005	TTT 73.8±0.9	TTC 72.5±0.8				
**G**	7	0.7	96905	50.8±0.5	0.682±0.005	GGT 43.5±0.7	GGC 52.3±1.0	GGA 53.9±1.0	GGG 60.6±1.3		
**H**	7	0.7	29476	74.9±0.8	0.749±0.008	CAT 75.4±1.0	CAC 74.4±1.5				
**I**	7	0.7	78589	59.3±0.7	0.714±0.006	ATT 58.6±1.0	ATC 58.5±0.8	ATA 69.5±1.5			
**K**	7	0.7	56045	72.6±0.9	0.737±0.008	AAA 71.7±1.1	AAG 75.7±1.4				
**L**	7	0.7	140106	42.5±0.4	0.637±0.004	TTA 39.9±0.7	TTG 47.7±1.0	CTT 47.1±1.1	CTC 58.2±0.7	CTA 63.7±1.5	CTG 36.1±0.5
**N**	7	0.7	50769	70.9±0.7	0.709±0.007	AAT 71.2±1.1	AAC 70.8±0.8				
**P**	7	0.7	58169	51.7±0.6	0.684±0.004	CCT 55.4±1.9	CCC 61.5±2.0	CCA 54.5±1.7	CCG 47.3±0.9		
**Q**	7	0.7	57880	74.0±0.6	0.746±0.005	CAA 76.2±1.0	CAG 72.9±0.9				
**R**	7	0.7	71795	45.4±0.4	0.715±0.011	CGT 40.7±0.5	CGC 43.0±0.6	CGA 57.2±2.6	CGG 56.4±2.0	AGA 63.4±3.6	AGG 62.4±5.7
**S**	7	0.7	75000	48.2±0.4	0.682±0.004	TCT 47.3±1.9	TCC 52.0±1.8	TCA 47.8±1.6	TCG 56.4±1.7	AGT 47.7±1.4	AGC 42.6±0.9

**T**	7	0.7	70325	55.5±0.6	0.709±0.006	ACT 57.9±1.7	ACC 52.3±0.6	ACA 55.1±1.3	ACG 59.4±1.1		
**V**	7	0.7	92999	52.9±0.5	0.676±0.005	GTT 49.0±1.2	GTC 57.6±1.4	GTA 59.2±1.0	GTG 50.2±1.0		
**Y**	7	0.7	37117	73.1±0.6	0.731±0.006	TAT 73.4±0.6	TAC 72.7±0.9				
**A**	7	0.75	124939	61.1±0.5	0.734±0.003	GCT 64.4±1.1	GCC 62.3±0.8	GCA 59.7±0.9	GCG 59.5±0.8		
**C**	7	0.75	15139	84.6±1.1	0.846±0.010	TGT 84.6±1.5	TGC 84.6±2.0				
**D**	7	0.75	67518	77.9±0.6	0.779±0.006	GAT 77.8±0.8	GAC 78.0±0.7				
**E**	7	0.75	75290	79.1±0.5	0.796±0.007	GAA 78.2±0.7	GAG 81.1±1.4				
**F**	7	0.75	50929	81.2±0.4	0.812±0.004	TTT 81.4±0.7	TTC 80.9±0.8				
**G**	7	0.75	96905	60.9±0.6	0.746±0.004	GGT 56.1±1.0	GGC 60.4±1.0	GGA 67.2±1.5	GGG 68.9±1.8		
**H**	7	0.75	29476	83.5±0.6	0.836±0.006	CAT 83.0±0.9	CAC 84.2±0.8				
**I**	7	0.75	78589	70.8±0.3	0.800±0.005	ATT 69.7±0.9	ATC 70.8±1.2	ATA 79.6±1.5			
**K**	7	0.75	56045	80.8±0.5	0.815±0.006	AAA 80.1±0.6	AAG 82.9±1.0				
**L**	7	0.75	140106	54.4±0.5	0.716±0.004	TTA 56.1±0.9	TTG 60.4±1.0	CTT 61.3±0.9	CTC 69.0±0.9	CTA 73.4±1.4	CTG 46.6±0.7

**N**	7	0.75	50769	79.3±0.7	0.793±0.008	AAT 79.5±1.5	AAC 79.1±0.7				
**P**	7	0.75	58166	63.9±0.6	0.761±0.005	CCT 67.5±1.5	CCC 72.6±1.8	CCA 67.0±1.4	CCG 59.8±1.2		
**Q**	7	0.75	57880	81.4±0.4	0.823±0.005	CAA 85.2±1.1	CAG 79.5±0.7				
**R**	7	0.75	71794	60.7±0.5	0.799±0.007	CGT 56.6±1.2	CGC 58.6±0.6	CGA 72.1±2.8	CGG 70.3±1.5	AGA 75.6±3.1	AGG 76.7±4.3
**S**	7	0.75	75000	62.8±0.7	0.766±0.006	TCT 63.1±1.9	TCC 66.1±1.6	TCA 64.1±0.6	TCG 67.9±0.9	AGT 62.8±2.1	AGC 57.4±1.0
**T**	7	0.75	70324	67.2±0.5	0.781±0.004	ACT 68.8±1.1	ACC 64.3±0.9	ACA 69.6±1.6	ACG 70.0±1.3		
**V**	7	0.75	92999	64.8±0.4	0.749±0.003	GTT 61.8±1.4	GTC 69.0±1.0	GTA 70.0±1.0	GTG 62.4±1.0		
**Y**	7	0.75	37117	81.6±0.8	0.814±0.008	TAT 82.5±1.3	TAC 80.4±0.8				
**A**	7	0.8	124936	73.4±0.4	0.816±0.004	GCT 75.3±1.0	GCC 75.4±0.8	GCA 72.4±1.0	GCG 71.6±0.5		
**C**	7	0.8	15138	91.3±0.5	0.913±0.005	TGT 90.7±0.6	TGC 91.8±1.0				
**D**	7	0.8	67516	86.5±0.3	0.865±0.004	GAT 86.2±0.7	GAC 86.9±1.1				
**E**	7	0.8	75288	86.3±0.4	0.867±0.003	GAA 85.5±0.5	GAG 88.0±0.5				
**F**	7	0.8	50927	89.3±0.3	0.891±0.004	TTT 89.9±0.2	TTC 88.4±0.9				

**G**	7	0.8	96904	73.5±0.3	0.826±0.004	GGT 70.0±0.6	GGC 72.6±0.8	GGA 79.3±1.2	GGG 79.8±1.2		
**H**	7	0.8	29476	91.2±0.5	0.912±0.005	CAT 91.3±0.8	CAC 91.1±0.7				
**I**	7	0.8	78589	82.4±0.3	0.877±0.005	ATT 82.4±0.7	ATC 81.6±0.5	ATA 87.4±1.2			
**K**	7	0.8	56043	88.4±0.5	0.891±0.005	AAA 87.7±0.6	AAG 90.5±1.1				
**L**	7	0.8	140103	69.1±0.4	0.807±0.003	TTA 72.7±0.5	TTG 74.5±1.4	CTT 75.5±1.2	CTC 79.9±0.6	CTA 82.0±1.1	CTG 62.2±0.7
**N**	7	0.8	50767	87.6±0.5	0.876±0.005	AAT 87.5±0.9	AAC 87.7±0.7				
**P**	7	0.8	58164	77.2±0.5	0.847±0.003	CCT 79.2±1.6	CCC 84.3±2.1	CCA 78.6±1.6	CCG 74.5±0.6		
**Q**	7	0.8	57880	88.7±0.3	0.890±0.004	CAA 89.6±0.9	CAG 88.3±0.5				
**R**	7	0.8	71791	76.4±0.3	0.883±0.007	CGT 73.9±1.0	CGC 75.1±0.9	CGA 83.2±1.4	CGG 81.8±1.8	AGA 86.1±2.1	AGG 86.7±2.9
**S**	7	0.8	74998	77.4±0.4	0.855±0.004	TCT 77.8±1.1	TCC 79.9±1.4	TCA 78.2±1.3	TCG 79.9±1.2	AGT 77.1±1.2	AGC 74.2±1.1
**T**	7	0.8	70323	79.2±0.4	0.862±0.003	ACT 80.1±1.3	ACC 77.9±0.8	ACA 80.9±1.0	ACG 80.0±0.5		
**V**	7	0.8	92998	77.5±0.4	0.836±0.003	GTT 76.1±0.9	GTC 80.1±0.9	GTA 80.7±0.6	GTG 75.7±0.6		
**Y**	7	0.8	37116	89.8±0.4	0.897±0.004	TAT 90.0±0.9	TAC 89.5±0.8				

**A**	7	0.85	124930	83.9±0.3	0.887±0.003	GCT 84.3±1.0	GCC 85.7±0.8	GCA 83.1±0.7	GCG 83.0±0.4		
**C**	7	0.85	15135	94.8±0.6	0.947±0.006	TGT 94.2±1.1	TGC 95.2±0.7				
**D**	7	0.85	67515	92.8±0.3	0.926±0.004	GAT 93.2±0.5	GAC 92.0±0.7				
**E**	7	0.85	75285	92.6±0.2	0.926±0.002	GAA 92.7±0.4	GAG 92.5±0.4				
**F**	7	0.85	50924	94.2±0.3	0.941±0.003	TTT 94.6±0.2	TTC 93.6±0.5				
**G**	7	0.85	96904	83.8±0.3	0.893±0.003	GGT 81.5±0.5	GGC 83.4±0.3	GGA 86.9±1.6	GGG 87.9±0.6		
**H**	7	0.85	29476	95.4±0.4	0.953±0.004	CAT 95.7±0.6	CAC 94.9±1.0				
**I**	7	0.85	78581	90.4±0.3	0.931±0.005	ATT 90.7±0.3	ATC 89.8±0.5	ATA 92.6±1.3			
**K**	7	0.85	56039	93.7±0.3	0.943±0.003	AAA 93.2±0.4	AAG 95.3±0.5				
**L**	7	0.85	140097	81.2±0.5	0.884±0.005	TTA 84.6±1.4	TTG 85.1±0.7	CTT 85.5±1.8	CTC 88.8±0.3	CTA 88.8±1.2	CTG 76.3±0.7
**N**	7	0.85	50766	93.3±0.4	0.932±0.004	AAT 92.7±0.6	AAC 93.7±0.4				
**P**	7	0.85	58163	86.5±0.3	0.910±0.002	CCT 87.4±0.8	CCC 90.9±1.2	CCA 87.0±1.5	CCG 85.0±0.4		
**Q**	7	0.85	57878	94.4±0.3	0.942±0.002	CAA 93.6±0.3	CAG 94.9±0.4				

**R**	7	0.85	71789	87.1±0.6	0.936±0.006	CGT 85.9±0.5	CGC 86.8±0.8	CGA 89.2±1.9	CGG 89.3±1.3	AGA 91.8±1.8	AGG 91.7±2.4
**S**	7	0.85	74993	87.7±0.2	0.919±0.004	TCT 87.2±0.9	TCC 88.6±1.1	TCA 87.1±1.3	TCG 88.9±0.9	AGT 87.4±0.7	AGC 87.3±0.8
**T**	7	0.85	70319	87.6±0.3	0.918±0.003	ACT 87.9±1.1	ACC 87.2±0.4	ACA 88.1±0.8	ACG 88.0±1.0		
**V**	7	0.85	92992	87.2±0.4	0.906±0.003	GTT 86.6±0.8	GTC 88.7±0.5	GTA 88.1±0.9	GTG 86.3±0.5		
**Y**	7	0.85	37115	94.6±0.3	0.945±0.002	TAT 95.1±0.4	TAC 93.8±0.3				
**A**	7	0.9	124925	91.1±0.3	0.937±0.002	GCT 90.2±0.6	GCC 92.4±0.7	GCA 90.0±0.5	GCG 91.2±0.5		
**C**	7	0.9	15135	96.4±0.4	0.963±0.004	TGT 96.1±0.8	TGC 96.6±0.5				
**D**	7	0.9	67514	96.2±0.1	0.961±0.002	GAT 96.2±0.3	GAC 96.0±0.5				
**E**	7	0.9	75276	96.2±0.2	0.962±0.002	GAA 96.3±0.3	GAG 96.2±0.4				
**F**	7	0.9	50923	96.9±0.2	0.969±0.002	TTT 97.0±0.3	TTC 96.8±0.3				
**G**	7	0.9	96902	91.0±0.3	0.940±0.003	GGT 89.4±0.5	GGC 91.3±0.6	GGA 92.3±1.2	GGG 92.5±0.4		
**H**	7	0.9	29473	97.2±0.4	0.973±0.004	CAT 97.2±0.6	CAC 97.3±0.3				
**I**	7	0.9	78578	94.8±0.2	0.963±0.002	ATT 94.7±0.3	ATC 94.7±0.3	ATA 96.1±0.9			

**K**	7	0.9	56036	96.8±0.2	0.970±0.002	AAA 96.5±0.3	AAG 97.6±0.4				
**L**	7	0.9	140087	89.7±0.3	0.937±0.004	TTA 92.2±1.3	TTG 91.5±0.8	CTT 92.4±0.7	CTC 94.4±0.7	CTA 94.3±1.1	CTG 86.7±0.5
**N**	7	0.9	50764	96.4±0.3	0.963±0.003	AAT 95.8±0.5	AAC 96.9±0.3				
**P**	7	0.9	58159	92.7±0.4	0.952±0.004	CCT 92.5±0.9	CCC 94.8±0.8	CCA 91.7±0.6	CCG 92.6±0.5		
**Q**	7	0.9	57874	97.0±0.2	0.968±0.002	CAA 96.2±0.3	CAG 97.4±0.3				
**R**	7	0.9	71785	93.0±0.3	0.968±0.003	CGT 92.2±0.4	CGC 93.2±0.4	CGA 93.5±1.4	CGG 93.9±0.7	AGA 95.4±1.4	AGG 95.4±1.2
**S**	7	0.9	74985	93.6±0.2	0.958±0.002	TCT 92.8±0.7	TCC 94.1±0.6	TCA 92.4±0.9	TCG 94.1±0.5	AGT 93.3±0.5	AGC 94.1±0.4
**T**	7	0.9	70317	92.8±0.3	0.954±0.002	ACT 91.9±1.1	ACC 92.9±0.4	ACA 92.7±0.9	ACG 93.4±0.5		
**V**	7	0.9	92988	92.9±0.4	0.950±0.002	GTT 92.5±0.8	GTC 93.7±0.7	GTA 92.6±0.4	GTG 92.8±0.6		
**Y**	7	0.9	37112	96.9±0.1	0.969±0.002	TAT 96.8±0.2	TAC 96.9±0.3				
**A**	7	0.95	124920	94.7±0.2	0.965±0.002	GCT 94.3±0.5	GCC 95.6±0.4	GCA 94.3±0.4	GCG 94.5±0.4		
**C**	7	0.95	15133	97.2±0.2	0.972±0.002	TGT 97.0±0.5	TGC 97.3±0.4				
**D**	7	0.95	67509	97.7±0.1	0.977±0.002	GAT 97.6±0.2	GAC 97.8±0.4				

**E**	7	0.95	75273	97.8±0.2	0.979±0.001	GAA 97.6±0.3	GAG 98.3±0.2				
**F**	7	0.95	50921	98.1±0.2	0.981±0.002	TTT 98.0±0.2	TTC 98.2±0.2				
**G**	7	0.95	96897	94.7±0.2	0.967±0.003	GGT 93.6±0.5	GGC 95.1±0.3	GGA 95.4±0.8	GGG 95.7±0.8		
**H**	7	0.95	29472	97.9±0.4	0.980±0.004	CAT 97.8±0.5	CAC 98.2±0.4				
**I**	7	0.95	78575	96.8±0.2	0.979±0.003	ATT 96.5±0.3	ATC 96.9±0.3	ATA 98.2±0.8			
**K**	7	0.95	56028	98.3±0.1	0.984±0.002	AAA 98.3±0.2	AAG 98.5±0.4				
**L**	7	0.95	140073	94.5±0.2	0.965±0.002	TTA 95.7±0.8	TTG 94.9±0.6	CTT 95.6±0.4	CTC 96.9±0.5	CTA 97.0±0.7	CTG 93.2±0.2
**N**	7	0.95	50762	97.6±0.2	0.976±0.002	AAT 97.3±0.4	AAC 97.9±0.3				
**P**	7	0.95	58156	95.5±0.3	0.970±0.002	CCT 94.6±0.6	CCC 96.9±0.8	CCA 93.9±0.6	CCG 96.0±0.4		
**Q**	7	0.95	57868	98.2±0.1	0.982±0.001	CAA 98.0±0.2	CAG 98.3±0.2				
**R**	7	0.95	71781	96.1±0.1	0.984±0.002	CGT 95.7±0.3	CGC 96.4±0.4	CGA 95.6±1.1	CGG 96.0±0.9	AGA 97.4±1.0	AGG 97.3±1.0
**S**	7	0.95	74984	96.1±0.2	0.975±0.002	TCT 95.1±0.5	TCC 96.4±0.5	TCA 95.5±0.9	TCG 96.2±0.6	AGT 95.8±0.7	AGC 96.7±0.3
**T**	7	0.95	70311	95.4±0.4	0.971±0.003	ACT 93.8±1.2	ACC 95.6±0.5	ACA 95.0±0.5	ACG 96.2±0.4		

**V**	7	0.95	92987	95.5±0.2	0.970±0.002	GTT 95.3±0.6	GTC 96.5±0.6	GTA 95.2±0.5	GTG 95.3±0.4		
**Y**	7	0.95	37109	97.7±0.2	0.978±0.002	TAT 97.5±0.3	TAC 98.0±0.4				
**A**	7	1	124918	96.0±0.1	0.974±0.001	GCT 95.5±0.4	GCC 96.9±0.4	GCA 95.4±0.4	GCG 95.9±0.3		
**C**	7	1	15131	97.7±0.3	0.977±0.003	TGT 97.6±0.5	TGC 97.7±0.5				
**D**	7	1	67507	98.1±0.2	0.981±0.002	GAT 97.8±0.3	GAC 98.5±0.3				
**E**	7	1	75272	98.3±0.1	0.984±0.001	GAA 98.1±0.1	GAG 98.6±0.2				
**F**	7	1	50921	98.3±0.1	0.984±0.001	TTT 98.1±0.2	TTC 98.6±0.2				
**G**	7	1	96895	95.7±0.1	0.974±0.002	GGT 95.0±0.6	GGC 95.8±0.3	GGA 96.1±0.7	GGG 96.7±0.7		
**H**	7	1	29472	98.2±0.3	0.982±0.003	CAT 97.9±0.5	CAC 98.5±0.2				
**I**	7	1	78572	97.4±0.2	0.985±0.001	ATT 97.0±0.3	ATC 97.7±0.3	ATA 99.2±0.5			
**K**	7	1	56028	98.7±0.2	0.987±0.002	AAA 98.6±0.2	AAG 98.7±0.4				
**L**	7	1	140073	95.8±0.2	0.973±0.002	TTA 97.0±0.5	TTG 96.1±0.7	CTT 96.6±0.5	CTC 97.7±0.6	CTA 97.7±0.7	CTG 94.8±0.3
**N**	7	1	50762	97.9±0.2	0.979±0.002	AAT 97.7±0.3	AAC 98.1±0.3				

**P**	7	1	58156	96.1±0.3	0.975±0.002	CCT 95.1±0.7	CCC 97.3±0.8	CCA 94.5±0.7	CCG 96.7±0.4		
**Q**	7	1	57867	98.5±0.2	0.984±0.002	CAA 98.3±0.3	CAG 98.6±0.2				
**R**	7	1	71780	96.6±0.1	0.988±0.001	CGT 96.4±0.2	CGC 96.7±0.4	CGA 96.4±1.0	CGG 96.8±0.7	AGA 97.7±1.1	AGG 98.0±1.0
**S**	7	1	74984	96.8±0.2	0.980±0.001	TCT 96.1±0.6	TCC 97.0±0.3	TCA 96.2±0.8	TCG 96.8±0.7	AGT 96.6±0.5	AGC 97.5±0.3
**T**	7	1	70310	96.1±0.3	0.976±0.002	ACT 94.6±1.0	ACC 96.4±0.4	ACA 95.7±0.6	ACG 96.8±0.5		
**V**	7	1	92985	96.4±0.2	0.978±0.001	GTT 96.3±0.4	GTC 97.2±0.5	GTA 95.8±0.6	GTG 96.3±0.4		
**Y**	7	1	37109	98.0±0.1	0.981±0.001	TAT 97.7±0.2	TAC 98.5±0.3				

Note:
ACC: overall accurancy
w: window size
p:percent of the matched score (s) to the expected maximal score (m)
All of the results were calculated based on a ten-fold cross validation.

Table S3. The codon usage in the reporter genes.

Codon	Amino Acid	Codon usage[a]	egfp-codon[b]	egfp-genscript[b]	mApple-codon[b]	mApple-genscript[b]
**GCA**	A	20.59	2	0	4	0
**GCC**	A	25.55	2	0	3	0
**GCG**	A	33	0	8	3	12
**GCT**	A	15.5	4	0	2	0
**TGC**	C	6.36	1	2	0	0
**TGT**	C	5.18	1	0	0	0
**GAC**	D	19.23	8	9	8	7
**GAT**	D	32.39	10	9	5	6
**GAA**	E	39.48	11	9	20	12
**GAG**	E	18.29	5	7	4	12
**TTC**	F	16.23	7	7	6	6
**TTT**	F	22.22	5	5	6	6
**GGA**	G	8.43	3	0	3	0
**GGC**	G	28.84	9	10	10	11
**GGG**	G	11.34	4	0	3	0
**GGT**	G	24.55	6	12	7	12
**CAC**	H	9.52	2	9	4	7
**CAT**	H	12.85	7	0	3	0
**ATA**	I	4.73	0	0	2	0
**ATC**	I	24.6	5	7	8	7
**ATT**	I	30.01	7	5	4	7
**AAA**	K	33.68	14	9	16	12
**AAG**	K	10.65	6	11	8	12

**CTA**	L	3.87	4	0	2	0
**CTC**	L	10.92	3	0	0	0
**CTG**	L	52.31	8	21	5	12
**CTT**	L	11.32	1	0	1	0
**TTA**	L	13.67	2	0	4	0
**TTG**	L	13.33	3	0	0	0
**ATG**	M	27.51	6	6	10	10
**AAC**	N	21.49	4	13	2	7
**AAT**	N	18.06	9	0	5	0
**CCA**	P	8.4	3	0	3	0
**CCC**	P	5.56	5	0	2	0
**CCG**	P	22.85	2	10	3	12
**CCT**	P	7.17	0	0	4	0
**CAA**	Q	15.03	6	4	4	3
**CAG**	Q	29.38	2	4	3	4
**AGA**	R	2.37	2	0	2	0
**AGG**	R	1.47	0	0	0	0
**CGA**	R	3.68	0	0	2	0
**CGC**	R	21.58	2	0	3	0
**CGG**	R	5.81	1	0	1	0
**CGT**	R	20.73	1	6	1	9
**AGC**	S	15.95	1	10	0	12
**AGT**	S	9.05	1	0	4	0
**TCA**	S	7.56	3	0	2	0
**TCC**	S	8.8	4	0	3	0
**TCG**	S	8.84	0	0	1	0

**TCT**	S	8.55	1	0	2	0
**ACA**	T	7.55	4	0	2	0
**ACC**	T	23	7	16	4	8
**ACG**	T	14.66	4	0	1	0
**ACT**	T	8.97	1	0	1	0
**GTA**	V	10.92	1	0	0	0
**GTC**	V	15.06	5	0	2	0
**GTG**	V	26.1	5	9	5	7
**GTT**	V	18.32	7	9	8	8
**TGG**	W	15.27	1	1	3	3
**TAC**	Y	12.21	5	6	7	6
**TAT**	Y	16.29	6	5	5	6

[a] The codon usage in the *E.coli*. [b] The number of the codon usage in the gene.
